# Conditional lethality profiling reveals anticancer mechanisms of action and drug-nutrient interactions

**DOI:** 10.1126/sciadv.adq3591

**Published:** 2024-10-04

**Authors:** Kyle M. Flickinger, Kelli M. Wilson, Nicholas J. Rossiter, Andrea L. Hunger, Paresh V. Vishwasrao, Tobie D. Lee, Carlos A. Mellado Fritz, Rebecca M. Richards, Matthew D. Hall, Jason R. Cantor

**Affiliations:** ^1^ Morgridge Institute for Research, Madison, WI 53715, USA.; ^2^ Department of Biochemistry, University of Wisconsin–Madison, Madison, WI 53706, USA.; ^3^ Division of Preclinical Innovation, National Center for Advancing Translational Sciences, National Institutes of Health, Rockville, MD, 20850, USA.; ^4^ Cellular and Molecular Biology Program, University of Michigan, Ann Arbor, MI 48109, USA.; ^5^ Division of Hematology, Oncology, and Bone Marrow Transplant, University of Wisconsin–Madison, Madison, WI 53706, USA.; ^6^ Early Translation Branch, National Center for Advancing Translational Sciences, National Institutes of Health, Rockville, MD, 20850, USA.; ^7^ Department of Biomedical Engineering, University of Wisconsin–Madison, Madison, WI 53706, USA.; ^8^ Carbone Cancer Center, University of Wisconsin–Madison, Madison, WI 53792, USA.

## Abstract

Chemical screens across hundreds of cell lines have shown that the drug sensitivities of human cancers can vary by genotype or lineage. However, most drug discovery studies have relied on culture media that poorly reflect metabolite levels in human blood. Here, we perform drug screens in traditional and Human Plasma–Like Medium (HPLM). Sets of compounds that show conditional anticancer activity span different phases of global development and include non-oncology drugs. Comparisons of the synthetic and serum-derived components that comprise typical media trace sets of conditional phenotypes to nucleotide synthesis substrates. We also characterize a unique dual mechanism for brivudine, a compound approved for antiviral use. Brivudine selectively impairs cell growth in low folate conditions by targeting two enzymes involved in one-carbon metabolism. Cataloged gene essentiality data further suggest that conditional phenotypes for other compounds are linked to off-target effects. Our findings establish general strategies for identifying drug-nutrient interactions and mechanisms of action by exploiting conditional lethality in cancer cells.

## Teaser

Chemical screens identify drug sensitivities that vary in conventional versus Human Plasma–Like Medium (HPLM).

## INTRODUCTION

Cell-based in vitro models are invaluable tools for drug discovery because they enable high-throughput screening based on phenotypes or defined molecular targets (*1*, *2*). Such models are also useful for investigating mechanisms of drug action and assessing how treatment responses might vary with cell-intrinsic factors (*3*). Despite a large increase in the catalog of reported drug sensitivities across hundreds of cell lines, most experimental therapeutics fail to translate—an issue attributed in part to limitations associated with traditional in vitro and in vivo models (*4*–*6*). Most in vitro studies rely on growth media that poorly resemble metabolite levels found in human blood (*7*, *8*), incubators that expose cells to oxygen levels far greater than those in different tissues (*9*), and plating formats that restrict many cell types to grow as two-dimensional (2D) monolayers (*10*). Direct in vivo screens for anticancer drugs are impractical, and cell growth conditions in animal models are more difficult to control and manipulate, limiting opportunities to interrogate cell-extrinsic contributions to treatment effects (*11*, *12*). Notably, there are also several differences in the nutrient composition of human versus mouse blood that may influence the behavior of engrafted human cells (*13*). Together, there is a central challenge to identify drug candidates in conditions more relevant to those in the human body and to understand how environmental factors affect drug activity.

Phenotypic screens enable drug discovery without the need for prior knowledge of a specific target or mechanism of action (*1*, *2*). Projects such as the Genomics of Drug Sensitivity in Cancer (GDSC) (*14*, *15*), the Cancer Target Discovery and Development (CTD2) (*16*, *17*), and the DepMap (*18*) have identified cancer therapeutic candidates using such screens across hundreds of cell lines, revealing intrinsic determinants of drug sensitivity. In addition, growing evidence has shown that treatment responses can vary with extrinsic conditions, including oxygen tension (*19*), 2D versus 3D culture (*20*–*22*), and the presence of stromal cells (*23*, *24*). Nonetheless, there has been limited consideration into how medium composition affects anticancer drug activity (*25*, *26*).

We previously developed Human Plasma–Like Medium (HPLM), a synthetic medium that contains more than 60 components at concentrations reflective of those in human blood (*13*). To create complete HPLM, we supplement with 10% dialyzed fetal bovine serum (FBS) (HPLM^+dS^), which contributes various biomolecules needed to support cell growth while minimizing the addition of undefined polar metabolites. Guided by unforeseen findings in metabolic regulation and conditional gene essentiality, we previously showed that culture in HPLM^+dS^ versus traditional media could affect cellular responses to an approved chemotherapeutic [5-fluorouracil (5-FU)] (*13*) and to a drug that has been tested in patients with cancer (CB-839) (*27*). Therefore, we reasoned that unbiased phenotypic screens could be used to reveal additional drugs that elicit medium-dependent anticancer effects. By comparing lethality profiles in HPLM and traditional media, it may be possible to identify drugs that offer larger therapeutic windows based on enhanced relative activity in nutrient conditions with greater relevance to human physiology. Moreover, drug-nutrient interactions could be exploited to nominate extrinsic biomarkers for treatment efficacy and may motivate future therapies that couple specific compounds with nutrient transport inhibitors or systemic metabolite manipulation.

Here, we perform chemical screens across a panel of leukemia cell lines grown in HPLM^+dS^ and conventional media. Analysis of these data reveals that compounds with conditional anticancer activity span different phases of drug development and include non-oncology drugs. Follow-up work traces a number of conditional phenotypes to the availability of nucleotide synthesis substrates and also uncovers a unique dual-targeting mechanism for brivudine (BVDU), an agent approved for antiviral use in some countries. Catalogued gene essentiality data further suggest that conditional phenotypes for other compounds may be linked to noncanonical targets. Together, these results demonstrate strategies to identify drug-nutrient interactions and mechanisms of action by systematically mapping and exploiting conditional lethality in human cancer cells.

## RESULTS

### High-throughput screens reveal compounds with conditional anticancer activity

**Fig. 1.**
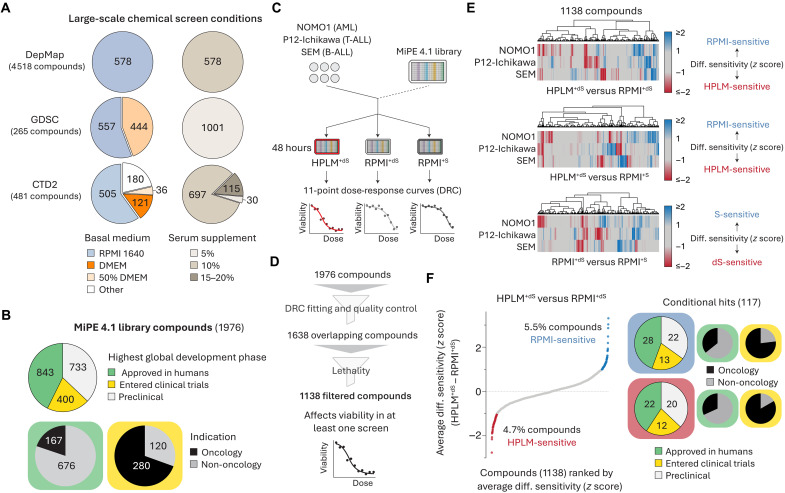
High-throughput screens reveal compounds with conditional anticancer activity. (**A**) Growth conditions across chemical screens from the DepMap, GDSC, and CTD2 projects. Fifty percent DMEM refers to basal media containing DMEM and another synthetic medium in a 1:1 mixture. (**B**) Highest global development phase and indication for compounds in the MiPE 4.1 library. (**C**) Schematic for chemical screens in blood cancer cell lines. AML, acute myeloid leukemia; B-ALL, B cell acute lymphoblastic leukemia; T-ALL, T cell ALL. (**D**) Schematic for dose-response curve quality control and filtering strategies to establish a set of overlapping compounds across chemical screen datasets. (**E**) Cluster maps showing conditional lethality phenotypes in three cell lines. (**F**) Compounds ranked by average HPLM^+dS^-RPMI^+dS^ phenotypes across three cell lines. Highest global development phase and indication for conditional hits.

Although phenotypic screens have uncovered drug candidates for a variety of diseases (*4*), they typically rely on traditional media composed of a synthetic reagent with limited relevance to nutrient levels in human blood and an FBS supplement that contributes a cocktail of undefined metabolites (*7*). This is well illustrated by cataloging the growth conditions used for chemical screens from the GDSC, CTD2, and DepMap projects. While these resources have produced useful drug sensitivity information for hundreds of cell lines, they are derived from screens mainly performed in RPMI- or Dulbecco’s Modified Eagle Medium (DMEM)–based media containing 5 to 20% FBS (Fig. 1A) (*14*–*18*).

To generate chemical lethality profiles in HPLM^+dS^ and RPMI-based media prepared with physiologic glucose (5 mM) and either standard (RPMI^+S^) or dialyzed FBS (RPMI^+dS^), we used the Mechanism Interrogation Plate (MiPE) 4.1 library—a collection of 1976 unique agents including preclinical compounds and drugs that have been either tested or approved in patients for cancer and various non-oncology indications (Fig. 1B, fig. S1A, and table S1) (*28*). We screened three human cell lines (NOMO1, P12-Ichikawa, and SEM) spanning different leukemia types against each MiPE compound over an 11-point concentration range (Fig. 1C). Next, we generated dose-response curves and then applied filtering strategies to minimize possible screening artifacts and to remove compounds that did not affect cell viability (Fig. 1D and fig. S1, B and C).

For each of the 1138 filtered compounds common across all screens, we defined a response score as the area under the dose-response curve. Of note, these scores were more strongly correlated by cell line than screening condition (fig. S1D). We then generated conditional lethality profiles by standardizing (*z* score) each set of differential response scores: (i) HPLM^+dS^-RPMI^+dS^; (ii) HPLM^+dS^-RPMI^+S^; and (iii) RPMI^+dS^-RPMI^+S^. Overall, these profiles showed variable degrees of overlap between cell lines (Fig. 1E). For each compound in each comparison, we averaged the *z* score across cell lines to improve detection of the strongest global hits. Using our averaged HPLM^+dS^-RPMI^+dS^ profile, we first assessed how treatment phenotypes were affected only by the differential availability of defined medium components. This analysis revealed 54 HPLM-sensitive and 63 RPMI-sensitive hits, of which just one-third were drugs either approved or tested for cancer therapy in humans (Fig. 1F).

### Conditional phenotypes for purine analogs are linked to hypoxanthine

**Fig. 2.**
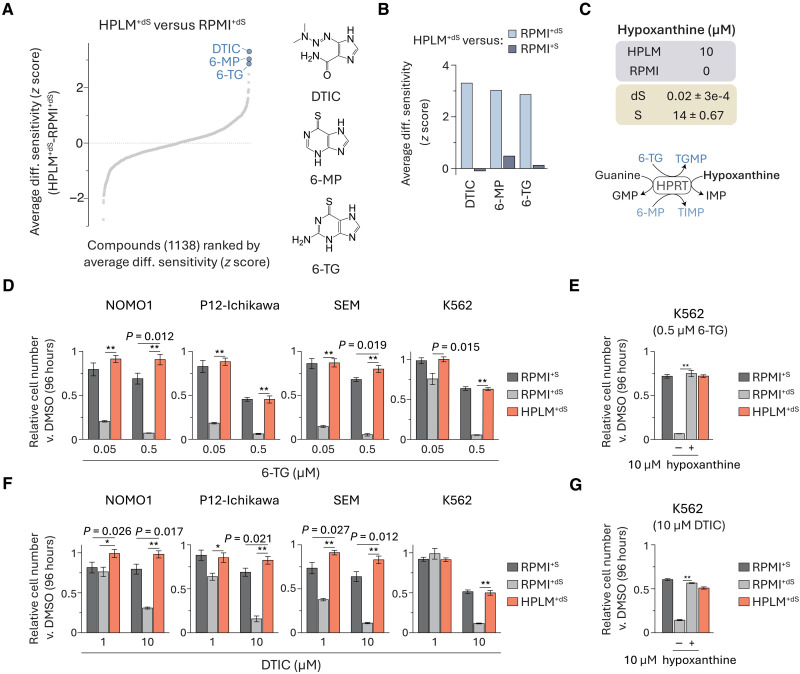
Conditional phenotypes for purine analogs are linked to hypoxanthine. (**A**) Compounds ranked by average HPLM^+dS^-RPMI^+dS^ phenotypes. (**B**) Conditional phenotypes for DTIC, 6-MP, and 6-TG from averaged HPLM^+dS^-RPMI^+dS^ and HPLM^+dS^-RPMI^+S^ profiles. (**C**) Defined hypoxanthine levels in HPLM and RPMI. Concentrations of hypoxanthine in 10% FBS (dS, dialyzed; S, untreated) (mean ± SD, *n* = 3). Schematic of reactions catalyzed by HPRT. (**D** to **G**) Relative growth of cells treated with 6-TG [(D) and (E)] or DTIC [(F) and (G)] versus DMSO (mean ± SD, *n* = 3, **P < 0.005 and * *P* < 0.01).

The top three RPMI-sensitive hits were purine analogs each approved for cancer therapy—dacarbazine (DTIC), 6-mercaptopurine (6-MP), and 6-thioguanine (6-TG) (Fig. 2A). Upon cell entry, 6-MP and 6-TG are enzymatically converted to effector metabolites that can inhibit de novo purine synthesis or be misincorporated into nucleic acids, whereas DTIC is often characterized as a DNA alkylating agent that first undergoes metabolic activation in the liver (fig. S2A) (*29*, *30*). Conditional phenotypes for these three drugs were not conserved in the HPLM^+dS^-RPMI^+S^ profile, suggesting that they may be linked to the availability of a defined component(s) that can also be differentially provided in standard versus dialyzed FBS (Fig. 2B). 6-MP and 6-TG are both non-endogenous substrates for hypoxanthine-guanine phosphoribosyltransferase (HPRT), which can convert guanine to guanosine monophosphate (GMP) and hypoxanthine to inosine monophosphate (IMP) in the purine salvage pathway (*31*). Although guanine is not defined in either medium, HPLM contains hypoxanthine at a concentration (10 μM) similar to levels contributed by 10% FBS before dialysis (Fig. 2C). Thus, we hypothesized that conditional phenotypes for 6-MP and 6-TG may be linked to hypoxanthine, which can compete with each drug for HPRT activity.

To assess drug sensitivity, we evaluated treatment responses at each of two doses spanning a 10-fold concentration range chosen to capture putative conditional effects. Given the potential to incorporate insights from our previous CRISPR screens in HPLM^+dS^ and traditional media (*27*), we extended these assays to the K562 chronic myeloid leukemia line, with dose-responses in these cells measured over an extended six-point concentration range.

Consistent with our screen results, 6-TG elicited 60 to 80% growth defects in RPMI^+dS^ versus both HPLM^+dS^ and RPMI^+S^ in all four cell lines (Fig. 2D and fig. S2B). As expected, the addition of HPLM-defined hypoxanthine to RPMI^+dS^ normalized this relative growth defect in K562 cells (Fig. 2E). We observed similar conditional responses to DTIC across cell lines, but higher doses were needed to impair cell growth versus those used for 6-TG (Fig. 2F and fig. S2C), and further, the addition of physiologic hypoxanthine also normalized DTIC-induced growth defects (Fig. 2G). These results suggest that DTIC can act as a weak purine analog beyond its canonical mechanism. Notably, our previous genome-wide CRISPR screens in HPLM^+dS^ or RPMI^+dS^ revealed that several genes with roles in the de novo purine synthesis pathway were RPMI-essential hits in K562 cells—conditional phenotypes that can also likely be linked to hypoxanthine availability (fig. S2D).

Of note, among the other RPMI-sensitive hits we identified was 5-azacytidine (5AzaC), a pyrimidine analog also approved for treating certain cancers (fig. S2E). Similar to the purine drugs described above, 5AzaC can be metabolized to derivatives that disrupt nucleic acid synthesis, and moreover, its conditional phenotype was not conserved to the HPLM^+dS^-RPMI^+S^ profile (fig. S2, F and G) (*29*). Metabolic activation of 5AzaC is initiated by uridine-cytidine kinase (UCK), which also catalyzes production of uridine monophosphate (UMP) from uridine in the pyrimidine salvage pathway. Similar to the case for hypoxanthine, uridine is uniquely defined in HPLM versus RPMI but provided at supraphysiologic levels by the standard FBS supplement (fig. S2H). Therefore, we reason that conditional phenotypes for 5AzaC are probably linked to the differential availability of uridine, which may compete with 5AzaC for UCK activity.

### Serum-derived thymidine alters cellular sensitivity to TYMS inhibitors

**Fig. 3.**
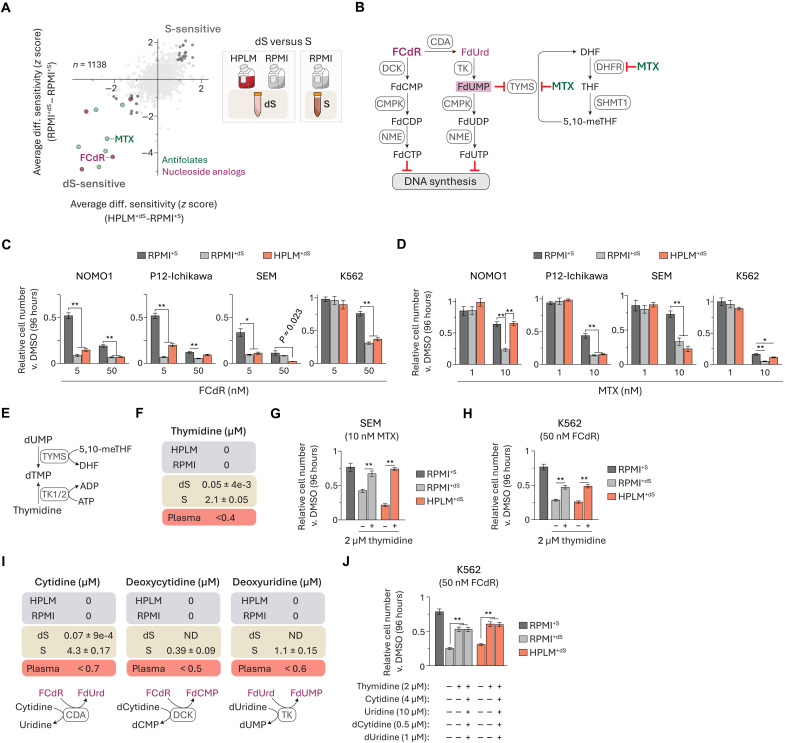
Serum-derived thymidine alters cellular sensitivity to TYMS inhibitors. (**A**) Comparison of averaged HPLM^+dS^-RPMI^+S^ and RPMI^+dS^-RPMI^+S^ phenotypes. dS-sensitive pyrimidine nucleoside analogs (purple) and antifolates (green). FCdR, fluorodeoxycytidine; MTX, methotrexate. (**B**) FCdR is converted to effector metabolites (left). MTX can inhibit TYMS and DHFR (right). (**C** and **D**) Relative growth of cells treated with FCdR (C) or MTX (D) versus DMSO (mean ± SD, *n* = 3, *P < 0.01, **P < 0.005). (**E**) Reactions catalyzed by thymidine kinase (TK) and TYMS generate dTMP. (**F** and **I**) Thymidine (F), cytidine, deoxycytidine, and deoxyuridine (I) are not defined in HPLM or RPMI. Levels of each in 10% FBS (dS, dialyzed; S, untreated) (mean ± SD, *n* = 3) and reported concentration ranges in human plasma (*100*, *101*). Reactions catalyzed by enzymes involved in FCdR metabolism that can also act on these pyrimidines (I). (**G** and **H**) Relative growth of cells treated with MTX (G) or FCdR (H) versus DMSO (mean ± SD, *n* = 3, **P < 0.005). (**J**) Relative growth of cells treated with FCdR versus DMSO (mean ± SD, *n* = 3, **P < 0.005).

Next, we asked how FBS dialysis influenced treatment phenotypes regardless of whether HPLM or RPMI served as the basal medium. We identified 19 “dS-sensitive” and 20 “S-sensitive” hits based on shared conditional phenotypes in the averaged HPLM^+dS^-RPMI^+S^ and RPMI^+dS^-RPMI^+S^ profiles (Fig. 3A). While most of the S-sensitive set consisted of investigational compounds, more than half of the dS-sensitive hits were antifolates and pyrimidine nucleoside analogs that in large part have been approved or tested for cancer therapy (fig. S3A). Antifolates inhibit critical enzymes involved in folate metabolism, including thymidylate synthase (TYMS) and dihydrofolate reductase (DHFR), while the pyrimidine nucleoside analogs are metabolically converted to fluoronucleotides that either interfere with nucleic acid synthesis or inhibit TYMS (Fig. 3B and fig. S3B) (*32*–*34*). Folate metabolism serves to activate and transfer one-carbon (1C) units that support several pathways. In mammalian cells, there are parallel 1C pathways in the cytosol and mitochondria that are often interlinked such that formate generated in the mitochondria is exported to the cytosol and used to support methionine regeneration and the de novo synthesis of both IMP and thymidylate (dTMP) (*35*, *36*). IMP is further metabolized to purine nucleotides that act as building blocks for nucleic acid synthesis and also support other cellular processes. dTMP is ultimately converted to deoxythymidine triphosphate (dTTP)—a critical substrate in DNA synthesis and DNA repair (*33*).

We investigated one hit from each of the two families enriched in the dS-sensitive subset. We first chose fluorodeoxycytidine (FCdR), a nucleoside analog with a variety of possible effector metabolites. As expected, FCdR induced 20 to 40% stronger growth defects across all four cell lines treated in the dS-supplemented conditions versus RPMI^+S^ (Fig. 3C and fig. S3C). We also selected methotrexate (MTX), a widely used antifolate (*31*, *37*). However, the differential responses to MTX were instead variable among the cell lines: 30 to 50% stronger in either (i) both dS-containing media (SEM and P12-Ichikawa) or (ii) RPMI^+dS^ alone (NOMO1) and (iii) more modest 5 to 10% relative defects versus RPMI^+S^ (K562) (Fig. 3D and fig. S3D). Notably, our previous CRISPR screens revealed that *TYMS* deletion in K562 cells caused differentially stronger effects in HPLM^+dS^ relative to RPMI^+S^ but not versus RPMI^+dS^, reflective of a comparable “dS-essential” phenotype (fig. S3E).

TYMS catalyzes the conversion of deoxyuridine monophosphate (dUMP) and 5,10-methylene-tetrahydrofolate (5,10-meTHF) to dTMP and dihydrofolate (DHF). Similar to the cases for other nucleotide salvage pathways, there are two human thymidine kinases (TK) that can each convert thymidine to dTMP but differ in subcellular localization (TK1, cytosolic; TK2, mitochondrial) (Fig. 3E) (*38*). Thymidine is not a defined component of RPMI and failed to meet inclusion criteria set in our design of HPLM given its submicromolar levels in human blood (*13*). Metabolite profiling analysis revealed that 10% FBS provides supraphysiologic thymidine (2 μM) that is depleted by more than 40-fold upon dialysis (Fig. 3F). Since TYMS inhibition is a mechanism shared between several antifolates and pyrimidine nucleoside analogs, we hypothesized that conditional phenotypes for FCdR and MTX could be linked to this overlooked source of thymidine. Consistent with prior evidence that thymidine availability can affect MTX efficacy (*39*–*41*), adding 2 μM thymidine to either HPLM^+dS^ or RPMI^+dS^ normalized MTX treatment responses in SEM cells (Fig. 3G). Notably, MTX is also a potent DHFR inhibitor, and previous work has shown that DHFR inhibition can be rescued by combined supplementation with thymidine and hypoxanthine since DHFR also supports de novo purine synthesis (*42*–*45*). Thus, we reason that conditional phenotypes for MTX are likely shaped by several factors, including cellular demands for purine versus thymidylate nucleotides, intrinsic nucleotide salvage pathway activities, and the availability of hypoxanthine.

Next, we examined how equivalent thymidine supplementation might affect FCdR-induced growth defects in K562 cells but observed just a 50% rescue versus RPMI^+S^ (Fig. 3H). Among the effector metabolites derived from FCdR is fluorodeoxyuridine monophosphate (FdUMP), which can form a covalent ternary complex with TYMS and 5,10-meTHF (*46*, *47*). Despite the incomplete rescue provided by thymidine, FdUMP was the only fluoronucleotide that we could detect in FCdR-treated K562 cells (fig. S3F). Given the possibility that other FCdR derivatives were below detection limits or had been incorporated into nucleic acids, we considered whether FBS might contribute other pyrimidines that can antagonize FCdR metabolism. Although we could detect three such examples in standard FBS (cytidine, deoxycytidine, and deoxyuridine), their combined addition did not provide any further rescue for FCdR-treated cells beyond thymidine alone (Fig. 3, I and J). These results suggest that FCdR cytotoxicity can be influenced by additional but currently unknown FBS-derived biomolecules.

Of note, similar to FCdR, 5-FU is a fluoropyrimidine that can be metabolized to FdUMP in a two-step pathway with floxuridine (FdUrd) acting as the intermediate product (fig. S3G) (*48*). FdUrd was also identified as a dS-sensitive hit, and TYMS inhibition is the primary mechanism of action most typically attributed to 5-FU (*33*, *34*). However, we previously identified a drug-nutrient interaction between 5-FU and uric acid that could be traced to a distinct branch of 5-FU metabolism (fig. S3H), with 5-FU cytotoxicity reduced in HPLM^+dS^ versus both RPMI-based media (*13*). When we further tested 5-FU against the four cell lines in our panel, we observed similar RPMI-sensitive phenotypes in each case (fig. S3, I and J). Together, these results suggest that relative 5-FU activity is also likely influenced by multiple factors, including uric acid availability and relative expression of thymidine phosphorylase, the enzyme responsible for converting 5-FU to FdUrd.

### Conditional BVDU sensitivity is linked to folic acid availability

**Fig. 4.**
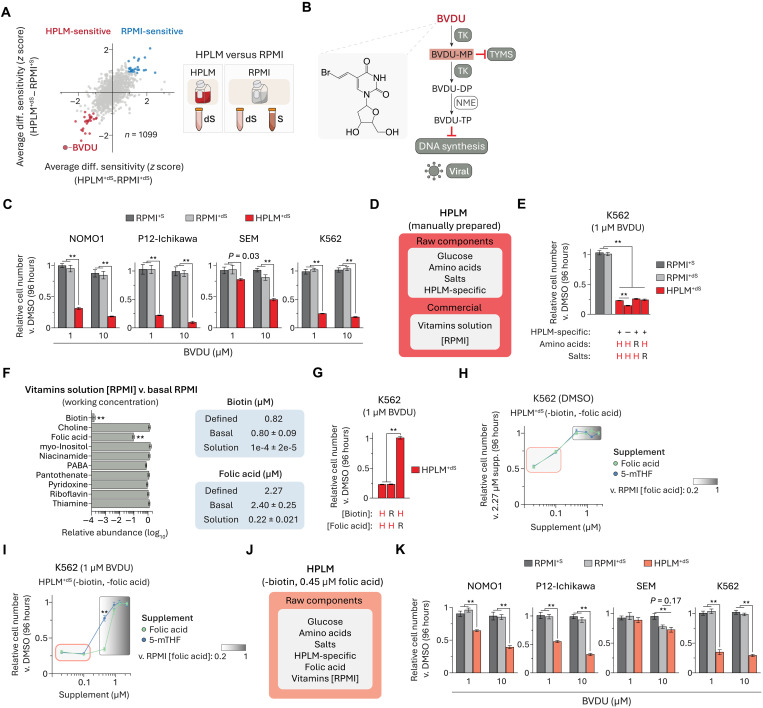
Conditional BVDU sensitivity is linked to folic acid availability. (**A**) Comparison of averaged HPLM^+dS^-RPMI^+dS^ and HPLM^+dS^-RPMI^+S^ phenotypes. (**B**) Schematic for the activation and canonical mechanism of BVDU in virally infected cells. Viral TK catalyzes reactions that convert BVDU to its mono (BVDU-MP) and diphosphate (BVDU-DP) forms. BVDU-MP can inhibit human and viral TYMS. BVDU-DP is metabolized to the active triphosphate derivative (BVDU-TP) that inhibits viral DNA polymerase. (**C** and **K**) Relative growth of cells treated with BVDU versus DMSO (mean ± SD, *n* = 3, **P < 0.005). (**D**) Components of manually prepared HPLM. (**E** and **G**) Relative growth of cells treated with BVDU versus DMSO (mean ± SD, *n* = 3, **P < 0.005). H, HPLM-defined concentrations. R, RPMI-defined concentrations. See table S2. (**F**) Relative working concentrations in commercial solution (Sigma-Aldrich, R7256, lot RNBB7627) versus basal RPMI (lot 2458379) (mean ± SD, *n* = 3, **P < 0.005). Defined and working concentrations of biotin and folic acid (mean ± SD, *n* = 3). PABA, para-aminobenzoic acid. (**H**) Relative growth of K562 cells in biotin- and folic acid–deficient HPLM^+dS^ supplemented with folic acid or 5-methyltetrahydrofolate (5-mTHF) (mean ± SD, *n* = 3). Red-outlined box, reported range in human plasma (see fig. S4). Gradient-shaded box, range from 0.45 to 2.27 μM. (**I**) Relative growth of K562 cells treated with BVDU versus DMSO in biotin- and folic acid–deficient HPLM^+dS^ supplemented with folic acid or 5-mTHF (mean ± SD, *n* = 3, **P < 0.005). (**J**) Components of a modified HPLM that lacks biotin and contains 0.45 μM folic acid. HPLM-based media used for experiments are distinguished by the shading used in this panel and in (D).

Next, we sought to identify drugs that caused differentially stronger responses in HPLM^+dS^ regardless of the FBS supplement added to RPMI. After removing the S-/dS-sensitive hits from consideration, we identified 26 HPLM-sensitive hits based on shared conditional phenotypes in the averaged HPLM^+dS^-RPMI^+dS^ and HPLM^+dS^-RPMI^+S^ profiles (Fig. 4A). The top-scoring HPLM-sensitive hit was BVDU, a drug that did not otherwise lead to a differential treatment phenotype between the two RPMI-based media (fig. S4A). BVDU can act as an antiviral agent against herpes simplex virus type 1 and varicella-zoster virus (VSV) and is approved for the treatment of herpes zoster (shingles) in several European countries (*49*, *50*). Like the case for other pyrimidine nucleoside analogs, evidence indicates that BVDU can be metabolized to effector products upon cell entry (*50*–*52*). Canonical BVDU selectivity against infected cells is attributed to the putative requirement for a viral TK to catalyze the reactions that sequentially convert BVDU to its monophosphate (BVDU-MP) and diphosphate (BVDU-DP) forms (*51*, *53*). While BVDU-DP can be further metabolized to the triphosphate form (BVDU-TP) that may disrupt viral DNA synthesis, prior work also suggests that BVDU-MP can inhibit human and viral homologs of TYMS (Fig. 4B) (*51*, *54*, *55*).

Consistent with our chemical screen results, BVDU elicited remarkably HPLM-specific 70 to 90% growth defects in three cell lines and more modest (30 to 40%) yet HPLM-dependent responses in the fourth line (SEM) (Fig. 4C). Moreover, when we tested BVDU against K562 cells across an expanded eight-point concentration range, we observed that even the lowest dose (10 nM) impaired growth by 30% in HPLM^+dS^, while the highest one (50 μM) induced only minor responses (<10%) in the two RPMI-based media (fig. S4B).

To determine why BVDU induced near-selective conditional sensitivity, we first considered possible links to the reported effector metabolites of canonical BVDU antiviral activity. Notably, the two human TKs display marked differences in kinase activity across several natural and non-endogenous substrates, including BVDU—a TK2-specific substrate based on activity assays using purified enzymes or human cell extracts (*56*–*58*). Although the TK2-mediated synthesis of BVDU-MP could lead to TYMS inhibition, we reasoned that this mechanism alone would be inconsistent with the lack of BVDU-induced growth impairments seen in RPMI^+dS^ which, like HPLM^+dS^, provides negligible thymidine. Moreover, conditional phenotypes for BVDU were distinct from those identified for known TYMS inhibitors within the set of dS-sensitive drugs described above. Together, the drug-nutrient interaction and mechanism of action underlying conditional BVDU sensitivity were not immediately apparent.

Basal HPLM contains glucose, amino acids, salts, and more than 30 additional components not otherwise defined in conventional synthetic media (table S2). While several vitamins have been deemed essential for mammalian cell culture (*59*), most failed to meet inclusion criteria set in our design of HPLM (*13*). Thus, we chose to incorporate vitamins at RPMI-defined levels by using a commercial solution as described elsewhere (Fig. 4D) (*13*). On the basis that glucose and vitamin levels were normalized between conditions, we systematically tested BVDU against K562 cells in HPLM derivatives with the remaining component subsets adjusted to match RPMI. However, each of these failed to rescue BVDU-induced growth defects, which were also modestly exacerbated in a derivative lacking the set of HPLM-specific components (Fig. 4E). Since we manually normalize the glucose levels between HPLM and RPMI, we then hypothesized that conditional BVDU effects might be linked to an unanticipated difference(s) in vitamin availability. To test this idea, we first measured vitamin levels in both basal RPMI and the commercial solution (Sigma-Aldrich, R7256) and then calculated the respective working concentrations. Although such concentrations were comparable for most cases, those for biotin and folic acid in the commercial solution were 10,000- and 10-fold lower, respectively, relative to expected values (Fig. 4F). In turn, when we treated K562 cells with BVDU in HPLM containing either biotin or folic acid at defined RPMI levels, we observed that the folic acid adjustment rescued the growth defect (Fig. 4G). Of note, we previously reported vitamin discrepancies between HPLM and RPMI based instead on metabolite profiling analysis of complete media (*13*, *27*).

Folic acid and 5-methyl-THF (5-mTHF) are the two most abundant folates in human blood; however, each is found at levels up to 100-fold lower than RPMI-defined folic acid (fig. S4C) (*60*–*62*). Therefore, to assess how the availability of these folates might affect BVDU sensitivity, we prepared biotin- and folic acid–deficient HPLM that otherwise contained eight remaining RPMI vitamins from manually prepared stocks and then systematically added either folic acid or 5-mTHF at concentrations spanning those reported in human plasma (20 to 100 nM) or over a fivefold range relative to RPMI-defined folic acid (0.45 to 2.27 μM). Across all concentrations, we first found that the growth of control K562 cells was unaffected by supplementing with 5-mTHF versus folic acid but that, in both cases, relative growth was reduced by 25 to 50% in the derivatives with plasma-like folate levels (Fig. 4H). By contrast, while BVDU markedly impaired cell growth in all derivatives with physiologic folate regardless of which species was added, 5-mTHF began to offer protection at a concentration (0.45 μM) twofold lower than was required for folic acid—possibly given that the major folate importer (SLC19A1) has a stronger affinity for 5-mTHF (Fig. 4I) (*63*). To preserve the HPLM-sensitive phenotype for BVDU while eliminating the baseline growth defects seen in plasma-like folate conditions, we used the HPLM derivative with 0.45 μM folic acid for follow-up experiments (Fig. 4J), whereby HPLM-based media are distinguished by the shaded colors depicted in Fig. 4 (D and J) (see the “Cell culture conditions” section).

Given the corresponding twofold boost in folic acid relative to HPLM^+dS^ prepared with the commercial vitamin solution, BVDU cytotoxicity across our cell line panel was partially reduced in this modified HPLM^+dS^ as expected (Fig. 4K and fig. S4D). Of note, K562 cells did not show a net exchange of folic acid regardless of BVDU treatment or initial folic acid availability in HPLM, suggesting that conditional BVDU sensitivity could not be attributed to a possible limiting depletion of exogenous folic acid (fig. S4E).

### TK2 expression is an intrinsic determinant of BVDU sensitivity

**Fig. 5.**
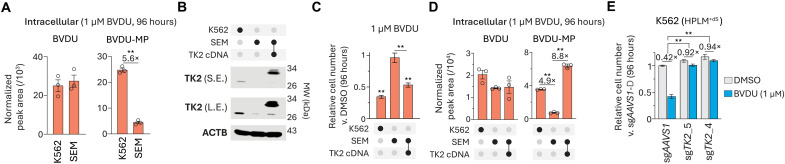
TK2 expression is an intrinsic determinant of BVDU sensitivity. (**A** and **D**) Cellular abundances of BVDU (left) and BVDU-MP (right) following BVDU treatment (mean ± SEM, *n* = 3, **P < 0.005). (**B**) Immunoblot for expression of TK2. Molecular weight (MW) standards are annotated. ACTB served as the loading control. S.E., short exposure; L.E., long exposure. TK2 cDNA was fused to 3xFLAG. (**C**) Relative growth of cells treated with BVDU versus DMSO (mean ± SD, *n* = 3, **P < 0.005). (**E**) Relative growth of *TK2*-knockout and control cells treated with BVDU versus vehicle-treated control cells in HPLM^+dS^ (mean ± SD, *n* = 3, **P < 0.005).

Since SEM cells showed markedly weaker BVDU sensitivity than the other three cell lines, we next hypothesized that either the uptake or putative metabolic activation of BVDU in these cells might be reduced. To test this idea, we sought to measure BVDU and its phosphorylated forms in SEM and K562 cells following treatment in HPLM^+dS^. Metabolite profiling revealed that BVDU levels were comparable between the two cell lines and that neither BVDU-DP nor BVDU-TP could be detected in either case, whereas BVDU-MP levels were fivefold lower in SEM cells (Fig. 5A). Regardless of whether BVDU-DP/-TP were below our detection limits, these results suggested that BVDU sensitivity depends on the formation of BVDU-MP as perhaps mediated by human TK2 in cells lacking viral TK. Consistent with this notion, TK2 expression was threefold lower in SEM versus K562 cells, and further, the expression of a *TK2* cDNA markedly increased the BVDU-MP levels and relative growth defects in BVDU-treated SEM cells (Fig. 5, B to D). Notably, when we instead treated these lines in HPLM^+dS^ with RPMI-defined folic acid, we observed little impact on cellular levels of both BVDU and BVDU-MP (fig. S5, A and B). Next, to confirm that TK2 expression is necessary to facilitate BVDU activity, we engineered *TK2*-knockout K562 cells, which showed no growth defect versus control cells but nearly complete resistance to BVDU in HPLM^+dS^ as expected (Fig. 5E). Collectively, these results suggested that relative TK2 levels influence BVDU sensitivity under low folate conditions, and further, that the drug-nutrient interaction between BVDU and folic acid was not linked to conditional differences in the metabolic activation of BVDU.

### BVDU-MP interferes with folate-dependent nucleotide synthesis

**Fig. 6.**
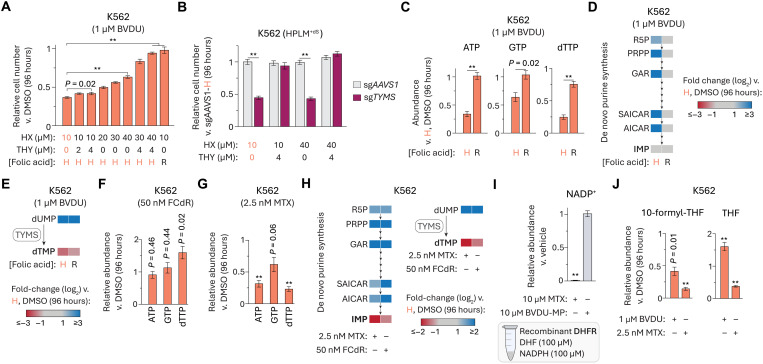
BVDU-MP interferes with folate-dependent nucleotide synthesis. (**A**) Relative growth of cells treated with BVDU versus DMSO (mean ± SD, *n* = 3, **P < 0.005). H, HPLM-defined concentration (0.45 μM); R, RPMI-defined concentration (2.27 μM). (**B**) Relative growth of *TYMS*-knockout and control cells versus control cells in HPLM^+dS^ (mean ± SD, *n* = 3, **P < 0.005). (**C**) Relative abundances of ATP, GTP, and dTTP in BVDU-treated and control cells versus control cells in HPLM^+dS^ (mean ± SEM, *n* = 3, **P < 0.005). (**D** and **E**) Heatmap of cellular abundances for indicated metabolites in BVDU-treated and control cells versus control cells in HPLM^+dS^ (*n* = 3). (**F** and **G**) Relative abundances of ATP, GTP, and dTTP in FCdR- (F) and MTX-treated (G) versus control cells in HPLM^+dS^ (mean ± SEM, *n* = 3, **P < 0.005). Drug doses selected to elicit growth defects comparable to those for BVDU-treated K562 cells in HPLM^+dS^. (**H**) Heatmap of cellular abundances for indicated metabolites in FCdR- and MTX-treated versus control cells in HPLM^+dS^ (*n* = 3). (**I**) Relative NADP^+^ levels measured from DHFR activity assays following addition of MTX or BVDU-MP (mean ± SEM, *n* = 3, **P < 0.005). (**J**) Relative abundances of indicated folate species in BVDU- and MTX-treated versus control cells in HPLM^+dS^ (mean ± SEM, *n* = 3, **P < 0.005).

Since our HPLM derivative with RPMI-defined amino acids (including methionine) did not provide any rescue effect to BVDU-treated K562 cells, we considered whether differential BVDU sensitivity may be linked to folate-dependent nucleotide synthesis. Nonetheless, given our rationale that a possible inhibition of TYMS alone could not explain the conditional phenotype for BVDU, we first sought to confirm that impaired growth specific to loss of TYMS activity could be restored with thymidine supplementation. Consistent with this notion, thymidine supplementation alone (up to 8 μM) had no impact on BVDU sensitivity, whereas elevating the HPLM levels of hypoxanthine (up to 80 μM) partially rescued the growth of BVDU-treated cells, and, in turn, combined addition of both supraphysiologic substrates enabled a complete rescue (Fig. 6A and fig. S6A). By contrast, engineered *TYMS*-knockout cells showed a marked growth defect that could be completely reversed by the addition of thymidine but was otherwise unaffected by increasing hypoxanthine availability (Fig. 6B and fig. S6B), thus confirming that thymidine salvage alone can complement for growth defects specific to a loss of TYMS activity.

Consistent with the dual supplementation approach necessary to reverse BVDU sensitivity, BVDU treatment markedly reduced cellular levels of adenosine triphosphate (ATP), guanosine triphosphate (GTP), and dTTP but had little impact on those of cytidine triphosphate (CTP) and uridine triphosphate (UTP)—effects that were largely reversed by adjusting the folic acid availability in HPLM to match RPMI (Fig. 6C and fig. S6C). Unbiased metabolite profiling revealed that BVDU treatment induced additional changes in the cellular metabolome (fig. S6D and table S3). Among these were a 40% reduction in IMP and elevated levels (10- to 20-fold) of several intermediates in the de novo purine synthesis pathway—effects resembling those attributed to a depletion of 10-formyl-THF the folate species that feeds into this pathway (Fig. 6D) (*64*). We also observed a marked 90-fold increase in dUMP levels and a 5-fold reduction in dTMP—the two pyrimidine components of the TYMS reaction (Fig. 6E). Overall, these BVDU-induced changes across the metabolome were largely reversed when cells were instead treated in HPLM^+dS^ with RPMI-defined folic acid, although less so for dUMP and the thymidine nucleotides. These results suggested that the increased folic acid availability relieved differential thresholds of impaired de novo purine and thymidylate synthesis.

Given that FCdR is also a pyrimidine nucleoside analog and that MTX can similarly inhibit folate-dependent nucleotide synthesis, we also asked how these two drugs affect cellular nucleotide pools at doses chosen to elicit comparable growth defects in HPLM^+dS^ (fig. S6E). FCdR treatment had little impact on the ATP and GTP levels but markedly increased (60 to 90%) those of dTTP, CTP, and UTP—changes that may be attributed to disrupted incorporation of pyrimidine nucleotides into nucleic acids (Fig. 6F and fig. S6F). Given that such changes were specific to treatment with FCdR versus BVDU, we reasoned that disrupted nucleic acid synthesis was unlikely a mechanism relevant to BVDU sensitivity. Across the same set of nucleoside triphosphates (NTPs), MTX treatment caused effects that more closely resembled those in BVDU-treated cells (Fig. 6G and fig. S6G). Extending our analysis to de novo purine synthesis intermediates and TYMS reaction components further revealed that treatment with FCdR or MTX led to changes largely reflective of those observed in BVDU-treated cells (Fig. 6H).

Collectively, these results suggested that conditional BVDU sensitivity could be traced to a hierarchical inhibition of the de novo IMP and dTMP synthesis pathways, whereby the demand for purine nucleotides must be met before the thymidylate limitation can be addressed in BVDU-treated cells. Of note, these results also likely explain why BVDU treatment in the HPLM derivative that lacked hypoxanthine led to modestly stronger responses (Fig. 4E).

### DHFR is not the molecular target of BVDU-MP

Since the conditional phenotypes for MTX and BVDU were considerably different despite inducing largely similar changes to the nucleotide metabolome, we reasoned that MTX and BVDU-MP act at least in part against distinct targets. Given the evidence suggesting that TYMS inhibition is an activity common to both compounds, we hypothesized that DHFR was instead a target specific to MTX versus BVDU-MP. To begin to test this idea, we developed a method to isolate high-purity BVDU-MP from reactions containing recombinant TK2 and, in turn, estimated the purified product concentration by referencing a panel of nucleoside monophosphate (NMP) standards (fig. S6, H to K). We then designed a mass spectrometry (MS)–based assay to evaluate DHFR activity based on measuring the production of nicotinamide adenine dinucleotide phosphate (NADP^+^) from reactions containing recombinant DHFR (fig. S6, L and M). As expected, the addition of MTX to these reactions abolished DHFR activity, whereas equivalent addition of BVDU-MP had a negligible impact (Fig. 6I).

We next asked whether cellular folate levels were differentially affected by treatment with MTX versus BVDU at doses again chosen to elicit comparable growth defects in HPLM^+dS^. In both cases, the pools of multiple folate species (including 10-formyl-THF) were markedly depleted, but while THF levels were also reduced (40%) upon MTX treatment, they were instead elevated (60%) in BVDU-treated cells (Fig. 6J and fig. S6N). Together, these results suggested that BVDU-MP does not act against DHFR. In addition, similar analysis following BVDU treatment in HPLM^+dS^ with RPMI-defined folic acid revealed minimal effects on these cellular folate pools (fig. S6O). Interestingly, the set of BVDU-induced changes across the folate profile instead resembled those reported for cells harboring either disruptions in the mitochondrial 1C pathway or loss of *MTHFD1*, a gene involved in the cytosolic 1C pathway (*45*).

### CRISPR screens uncover genetic contributions to BVDU sensitivity

**Fig. 7.**
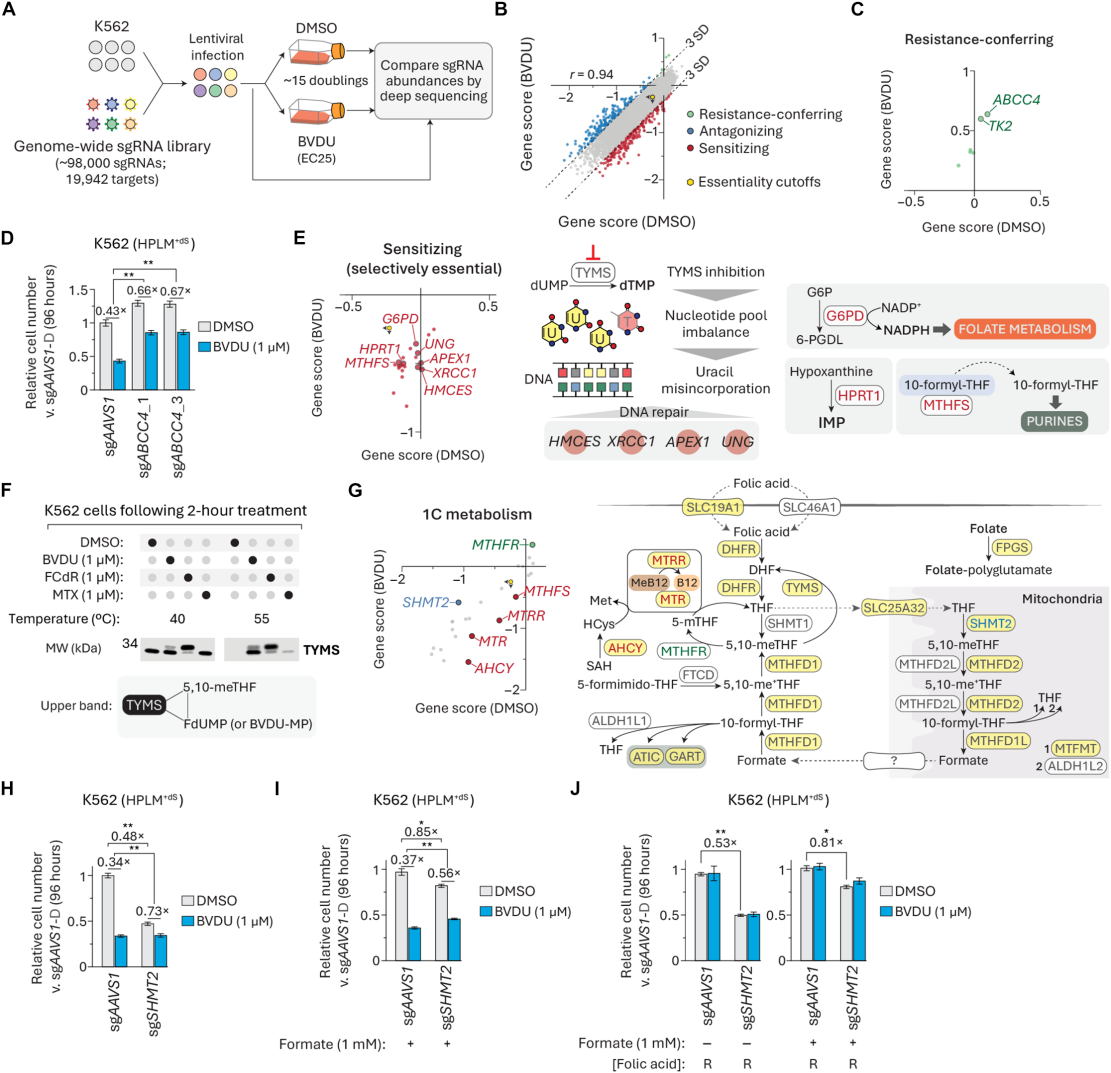
CRISPR screens uncover genetic contributions to BVDU sensitivity. (**A**) Schematic for genome-wide CRISPR screens in DMSO- and BVDU-treated K562 cells. (**B**) Comparison between DMSO- and BVDU-treated gene scores. Three types of screen hits are highlighted on the basis of differential gene score and essentiality. Essentiality cutoffs in each screen (origin, yellow hexagon). *r*, Pearson’s correlation coefficient. (**C**) Resistance-conferring hits. (**D**) Relative growth of *ABCC4*-knockout and control cells treated with BVDU versus vehicle-treated control cells in HPLM^+dS^ (mean ± SD, *n* = 3, **P < 0.005). (**E**) Selectively essential BVDU-sensitizing hits. Dashed arrow indicates noncatalytic delivery of 10-formyl-THF to the purinosome. (**F**) Immunoblot for expression of TYMS in cells after treatment with DMSO, BVDU, FCdR, or MTX in HPLM^+dS^ at indicated CETSA temperatures (top). Schematic depicting that the upper band corresponds to covalent ternary complex involving TYMS (bottom). (**G**) Comparison between DMSO- and BVDU-treated gene scores for a set of 23 genes involved in 1C metabolism (see table S4). Schematic of 1C metabolism. Yellow shading, encoding gene scored as essential in both screens. (**H** to **J**) Relative growth of *SHMT2*-knockout and control cells treated with BVDU versus vehicle-treated control cells in HPLM^+dS^ (mean ± SD, *n* = 3, **P < 0.005 and *P < 0.01).

To generate additional insights into the mechanism of conditional BVDU activity, we next sought to identify genetic modifiers of BVDU sensitivity. Using a genome-wide single guide RNA (sgRNA) library, we performed CRISPR screens on K562 cells in HPLM^+dS^ containing either vehicle or BVDU at a dose that caused a 25% growth defect in the relevant culture format (Fig. 7A and fig. S7A). For each gene, we calculated a scaled gene score and probability of dependency in each condition (table S4). The scores from each screen could discriminate core essential genes from a reference set of nonessential genes, and the two respective datasets also contained a comparable number of essential genes (probability of dependency > 0.5) (fig. S7, B and C) (*65*). We defined three types of screen hits based on differential gene score and essentiality: (i) resistance-conferring (positive and non-essential in both screens), (ii) antagonizing (positive and essential in at least one screen), and (iii) sensitizing (negative) (Fig. 7B).

Consistent with our BVDU treatment results in *TK2*-knockout K562 cells, *TK2* was among the two strongest resistance-conferring hits, along with *ABCC4*—a member of the ATP-binding cassette transporter family (Fig. 7C). By contrast to the case for *TK2*-knockout, *ABCC4* deletion led to a more modest 20 to 25% rescue of BVDU-induced growth defects (Fig. 7D). We considered whether ABCC4 might help mediate BVDU uptake but found that loss of *ABCC4* had little impact on cellular BVDU levels (fig. S7D). Prior work indicates that ABCC4 can act as an efflux pump for folic acid (*66*), suggesting that *ABCC4* deletion might perhaps confer partial BVDU resistance by effectively increasing the cellular folate pools. We also identified a small number of selectively essential antagonizing hits, but possible links between these genes and BVDU sensitivity are not immediately apparent (fig. S6E).

Our analysis also revealed a set of sensitizing hits that only scored as essential in the BVDU-treated cells, including genes involved in DNA repair (*HMCES*, *XRCC1*, *APEX1*, and *UNG*) (Fig. 7E). We reasoned that each of these knockouts could amplify the lethal effects of TYMS inhibition, which causes deoxynucleoside triphosphate (dNTP) pool imbalances and uracil misincorporation into DNA, resulting in DNA damage and thymineless cell death (*33*, *34*). Uracil-DNA glycosylase (UNG) is involved in the base-excision repair (BER) pathway and catalyzes the removal of misincorporated uracil bases from DNA, leaving apurinic-apyrimidinic (AP) sites that can be cleaved by AP endonuclease 1 (APEX1). x-ray repair cross complementing 1 (XRCC1) serves as a scaffold protein in BER (*67*) and 5-hydroxymethylcytosine binding, ES cell specific (HMCES) was recently characterized as a sensor/shield of AP sites (*68*). Notably, *TYMS* was not a BVDU modifier hit but instead scored as an essential gene in both screens. This analysis also identified *HPRT1*, suggesting greater dependence on the purine salvage pathway, as could be expected upon BVDU-mediated disruption of de novo purine synthesis. Other genes in this set of hits encoded enzymes linked to folate metabolism (*G6PD* and *MTHFS*). Previous work in colon cancer cells reported that *G6PD* deletion caused defects in folate-dependent biosynthesis (*69*). In addition, others have proposed that 5,10-methynyl-THF synthetase (MTHFS) may enhance de novo purine synthesis by delivering 10-formyl-THF to the purinosome (*70*). Thus, we reason that the loss of either *G6PD* or *MTHFS* could also exacerbate a BVDU-induced impairment of folate-dependent nucleotide synthesis.

Given the set of identified hits involved in BER and that thymidine addition was necessary to completely reverse BVDU sensitivity in HPLM^+dS^, we reasoned that BVDU treatment could at least in part lead to TYMS inhibition in K562 cells. To test this idea, we performed a one-dose cellular thermal shift assay (CETSA) following acute treatment with either BVDU, MTX, or FCdR. In each case, TYMS was stabilized at an otherwise denaturing temperature, indicative of ligand binding (Fig. 7F and fig. S7F). Consistent with the notion that TYMS can form a covalent ternary complex with FdUMP and 5,10-meTHF, FCdR treatment resulted in two bands reflecting TYMS in its free form and as part of this complex. We detected a similar pair of bands from BVDU-treated cells, suggesting that TYMS can form a similar although perhaps weaker ternary complex involving BVDU-MP, as these band intensities instead favored free versus bound TYMS. When we performed CETSA instead following acute treatments in HPLM^+dS^ with RPMI-defined folic acid, we observed little impact on the formation of the ternary complexes (fig. S7G). Together, these data suggest that conditional BVDU activity can be partially attributed to TYMS inhibition.

Next, we extended our screen analysis to a set of more than 20 genes that participate in 1C metabolism, with many scored as essential in both screens (Fig. 7G). Nonetheless, we identified a small number of modifier hits from this set. In addition to *MTHFS*, other BVDU-sensitizing genes encode enzymes that promote the methionine synthase (MTR) reaction, which generates THF and methionine from 5-mTHF and homocysteine (*AHCY*, *MTR*, and *MTRR*). By trapping folates as 5-mTHF, these knockouts likely synergize with BVDU to impair nucleotide synthesis by depleting a major source of THF (*71*, *72*). The only gene within this panel that nearly scored as a resistance-conferring hit was *MTHFR*, whose loss could likely result in elevated levels of 5,10-meTHF, the cosubstrate for TYMS. We also identified just one BVDU-antagonizing hit from this set: *SHMT2*, which encodes the enzyme that initiates mitochondrial catabolism of serine to formate.

Given that BVDU induced a set of changes to the folate profile similar to those reported for cells with a mitochondrial 1C pathway disruption, we considered whether serine hydroxymethyltransferase 2 (SHMT2) might be a target of BVDU-MP. To investigate this idea, we engineered *SHMT2*-knockout K562 clonal cells, which showed a 50% relative growth defect and reduced BVDU sensitivity, confirming both the *SHMT2* essentiality and gene-drug interaction suggested by our screens (Fig. 7H and fig. S7H). However, this apparently weaker treatment response could be largely attributed to the severe growth defect caused by *SHMT2* deletion alone, whereas those observed upon loss of *TK2* or *ABCC4* instead reflected a reversal of BVDU lethality. In addition, *SHMT2*-knockout and BVDU treatment had similar effects on cellular levels of ATP, GTP, and de novo purine synthesis intermediates, but *SHMT2* deletion caused a more modest reduction in cellular dTTP and instead markedly depleted the dUMP pool (fig. S7, I and J). Prior work has shown that formate supplementation helps support the growth of cells that lack a complete mitochondrial 1C pathway (*64*, *73*, *74*). Consistent with this notion, the addition of high formate (1 mM) largely reversed the baseline growth defects in *SHMT2*-knockout cells but otherwise offered little protection against BVDU (Fig. 7I). By contrast, increasing folic acid availability did not restore the growth of *SHMT2*-knockout cells but did reverse BVDU sensitivity regardless of either *SHMT2* deletion or formate supplementation (Fig. 7J). Together, these results suggested that BVDU-MP likely interferes with de novo purine synthesis by acting against a cytosolic target downstream of the mitochondrial 1C pathway.

### BVDU-MP affects the 10-formyl-THF synthetase activity of MTHFD1

**Fig. 8.**
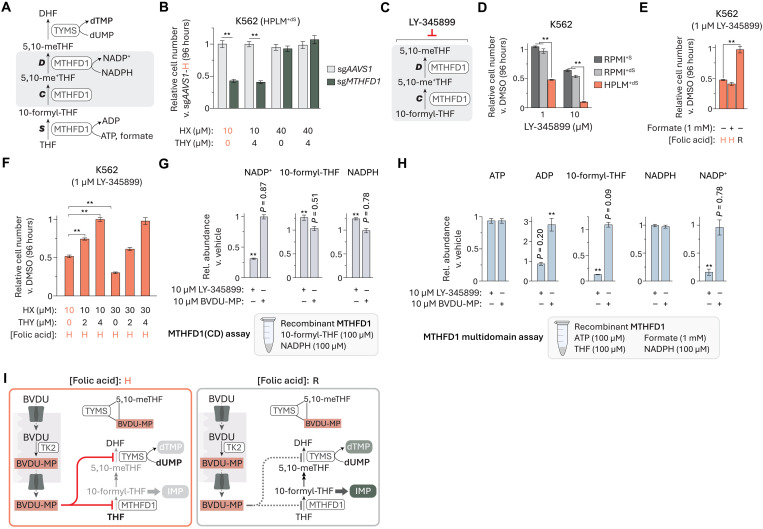
BVDU-MP affects the 10-formyl-THF synthetase activity of MTHFD1. (**A**) Schematic for reactions catalyzed by MTHFD1 in 1C metabolism. MTHFD1 activities: S, 10-formyl-THF synthetase; C, 5,10-methynyl-THF cyclohydrolase; D, 5,10-meTHF dehydrogenase. (**B**) Relative growth of *MTHFD1*-knockout and control cells versus control cells in HPLM^+dS^ (mean ± SD, *n* = 3, **P < 0.005). (**C**) LY-345899 can inhibit the CD activities of MTHFD1. (**D** to **F**) Relative growth of cells treated with LY-345899 versus DMSO (mean ± SD, *n* = 3, **P < 0.005). H, HPLM-defined concentration (0.45 μM); R, RPMI-defined concentration (2.27 μM). (**G** and **H**) Relative levels of indicated metabolites measured from MTHFD1(CD) (G) or multidomain MTHFD1 (H) activity assays following addition of LY-345899 or BVDU-MP (mean ± SEM, *n* = 3, **P < 0.005). (**I**) Proposed model for the dual-targeting mechanism of BVDU.

Since *MTHFD1* was the only cytosolic 1C pathway gene whose loss led to a set of changes in the cellular folate profile similar to those seen following BVDU treatment (*45*), we considered whether MTHFD1 might be the other relevant target of BVDU-MP. MTHFD1 is a tri-functional enzyme composed of two domains (*75*): an N-terminal 5,10-methynyl-THF cyclohydrolase and 5,10-meTHF dehydrogenase (CD) domain, and a C-terminal 10-formyl-THF synthetase (S) domain (Fig. 8A). Like the case for *TYMS*, *MTHFD1* was not identified as a hit gene in the CRISPR screens described above and also scored as essential regardless of BVDU treatment. MTHFD1(S) serves an important role in de novo purine synthesis by catalyzing the production of cytosolic 10-formyl-THF, as evidence indicates that loss of *MTHFD1* can result in purine auxotrophy (*45*, *76*). Consistent with this, engineered *MTHFD1*-knockout K562 cells showed a 60% growth defect in HPLM^+dS^ that could be rescued by increasing the availability of hypoxanthine (40 μM) but was otherwise unaffected by the addition of thymidine (4 μM) (Fig. 8B and fig. S8A). Similar to the case for *SHMT2* deletion, *MTHFD1*-knockout led to changes in the cellular levels of ATP, GTP, and de novo purine synthesis intermediates comparable to those observed in BVDU-treated cells but to a more modest reduction in dTTP levels and a marked depletion of the dUMP pool (fig. S8, B and C).

Prior studies have reported compounds that can inhibit the CD activities of MTHFD1 or the related mitochondrial MTHFD2, including LY-345899—a folate analog with sevenfold stronger affinity for MTHFD1 (Fig. 8C) (*77*, *78*). When we treated K562 cells with LY-345899 in HPLM^+dS^ versus RPMI-based media, we observed 40 to 50% stronger growth defects that could be normalized by adjusting folic acid availability but not by supplementing with high formate (1 mM) (Fig. 8, D and E). These results suggest that MTHFD1(CD) is likely the more relevant target of LY-345899 in K562 cells. Since MTHFD1(CD) is downstream of MTHFD1(S) in the cytosolic 1C pathway, we reasoned that this conditional phenotype could be attributed to a specific disruption of de novo dTMP synthesis. As anticipated, the addition of high thymidine (4 μM) fully rescued LY-345899–induced growth defects in HPLM^+dS^, whereas an elevation in hypoxanthine (40 μM) exacerbated the response—an effect consistent with prior work evaluating MTHFD1(CD) inhibitors (Fig. 8F) (*79*, *80*). Given the distinct complementation approach required to rescue BVDU-treated cells, these results suggested that BVDU-MP unlikely targets MTHFD1(CD) despite the similar drug-nutrient interaction revealed between LY-345899 and folic acid.

Using MS-based assays, we then sought to evaluate how LY-345899 and BVDU-MP might differentially affect the domain-specific activities of recombinant MTHFD1. When we added LY-345899 to reactions containing only the two substrates specific to MTHFD1(CD), 10-formyl-THF, and NADPH, we observed a 70% decrease in NADP^+^ production as expected, whereas equivalent addition of BVDU-MP had little impact (Fig. 8G and fig. S8, D and E). Next, we assessed product formation from reactions only containing the three substrates specific to MTHFD1(S)—ATP, THF, and formate—but detected negligible amounts of 10-formyl-THF and ADP. However, upon further addition of NADPH, the final substrate input across both MTHFD1 domains, we could detect both MTHFD1(S) reaction products and NADP^+^ (fig. S8, F to H). By using this multidomain assay, we found that LY-345899 decreased NADP^+^ production and markedly reduced 10-formyl-THF levels, whereas BVDU-MP caused a threefold increase in ADP but had little effect across the remaining components detected (Fig. 8H). Moreover, the equivalent addition of either BVDU or MTX did not affect the relative abundances across any of these reaction components (fig. S8I). Notably, previous work with prokaryotic MTHFD1(S) orthologs has suggested that the synthetase reaction proceeds according to a multistep mechanism, whereby production of a formyl phosphate intermediate is followed by ADP release and ensuing formylation of THF—effectively decoupling the synthesis of each reaction product (*81*). Given that only BVDU-MP affected the relative levels of one such putatively uncoupled product in our multidomain assay, we reason that these results offer further evidence that BVDU-MP likely acts against MTHFD1(S).

Collectively, we propose a dual-mechanism model to explain conditional BVDU sensitivity in cancer cells (Fig. 8I). TK2 catalyzes the phosphorylation of BVDU to BVDU-MP regardless of folate availability. Upon export to the cytosol, BVDU-MP can affect the activity of MTHFD1(S) and also bind TYMS, with a small fraction of this binding pair further interacting with 5,10-meTHF to form a covalent ternary complex. 10-formyl-THF acts as a cosubstrate for each of two steps in the de novo purine synthesis pathway and can also be metabolized along the cytosolic 1C pathway, resulting in the production of 5,10-meTHF, the cosubstrate of TYMS. Under low folate conditions, BVDU-MP can sufficiently inhibit MTHFD1(S) activity to reduce 10-formyl-THF levels below a critical threshold, leading to the depletion of downstream folates (including 5,10-meTHF) and a hierarchical impairment of de novo purine and thymidylate synthesis. By contrast, folate conditions have little impact on the formation of the ternary complex involving TYMS and BVDU-MP, likely explaining why increased folic acid availability led to a more complete reversal of BVDU-induced effects on cellular levels of folates and purine nucleotides than on the TYMS reaction components.

### BVDU treatment reduces in vivo tumor burden

Given the reported concentration ranges for folic acid (5 to 10 nM) and 5-mTHF (100 to 200 nM) found in mouse plasma (*71*, *72*), we also considered whether BVDU treatment affects leukemia xenograft growth. Since BVDU undergoes substantial first-pass metabolism to bromovinyluracil (*82*), we first sought to select a treatment dose based on the relative detection of BVDU in plasma collected 6 hours after drug injection. When we tested doses either comparable to or fivefold lower than those previously reported for intraperitoneal administration of BVDU in mice (*83*), we could only detect BVDU in plasma collected from mice treated at the higher dose (fig. S9A). Next, we injected Akaluciferase-expressing K562 cells into NOD scid gamma (NSG) mice and then treated daily with either vehicle or BVDU (fig. S9B). As anticipated, the tumor-bearing mice that received BVDU showed a 50% reduction in bioluminescent signal after 1 week, although this relative effect was partially diminished following an additional week of therapy using the same fixed dosage (fig. S9, C to F). In addition, we found that BVDU treatment caused little impact on plasma levels of creatinine and urea—traditional markers of kidney toxicity (fig. S9G) (*84*). Together, these results establish that BVDU treatment can reduce K562 tumor burden in vivo and further suggest that additional optimization of BVDU dosing, scheduling, and administration may lead to even more pronounced and sustained anticancer effects in vivo without enhancing toxicity.

### Conditional phenotypes for additional compounds are linked to folic acid

**Fig. 9.**
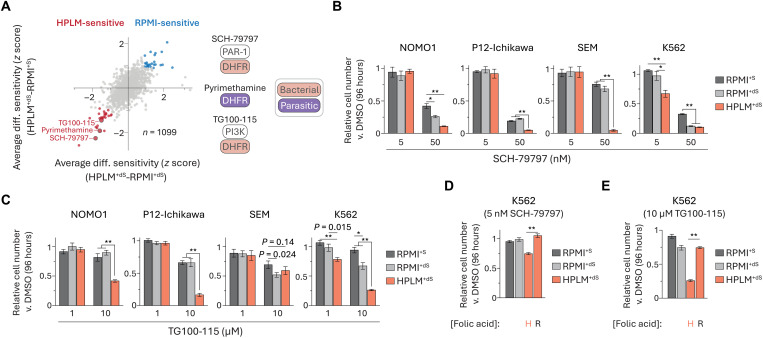
Conditional phenotypes for additional compounds are linked to folic acid. (**A**) Comparison of averaged HPLM^+dS^-RPMI^+dS^ and HPLM^+dS^-RPMI^+S^ phenotypes. Highlighted hits can act on orthologs of DHFR. Canonical targets of SCH-79797 and TG100-115 are unrelated to 1C metabolism. (**B** to **E**) Relative growth of cells treated with SCH-79797 [(B) and (D)] or TG100-115 [(C) and (E)] versus DMSO (mean ± SD, *n* = 3, **P < 0.005).

Next, we considered whether additional hits from our screens might be traced to the unanticipated difference in folic acid availability as well. For example, prior studies have shown that other HPLM-sensitive compounds can act on DHFR orthologs: (i) SCH-79797, a protease activated receptor-1 antagonist also characterized as a bacterial DHFR inhibitor (*85*); (ii) TG100-115, a PI3K inhibitor also recently identified as a stabilizer of bacterial DHFR (*86*); and (iii) pyrimethamine, a parasitic DHFR inhibitor (Fig. 9A). We chose to further investigate the two hits that share bacterial DHFR as a noncanonical target. As expected, SCH-79797 elicited stronger growth defects across all four cell lines treated in HPLM^+dS^ versus our RPMI-based media, although these differences ranged from 15 to 70% (Fig. 9B and fig. S10A). We observed similar medium-dependent responses to TG100-115, but much higher doses versus those used for SCH-79797 were required to impair cell growth and the conditional effects were shared among only three of the four cell lines (Fig. 9C and fig. S10B). Consistent with our rationale, adjusting the folic acid availability in HPLM to match RPMI normalized the growth defects induced by each drug in K562 cells (Fig. 9, D and E).

### Gene essentiality data suggest that other conditional phenotypes are linked to noncanonical mechanisms

**Fig. 10.**
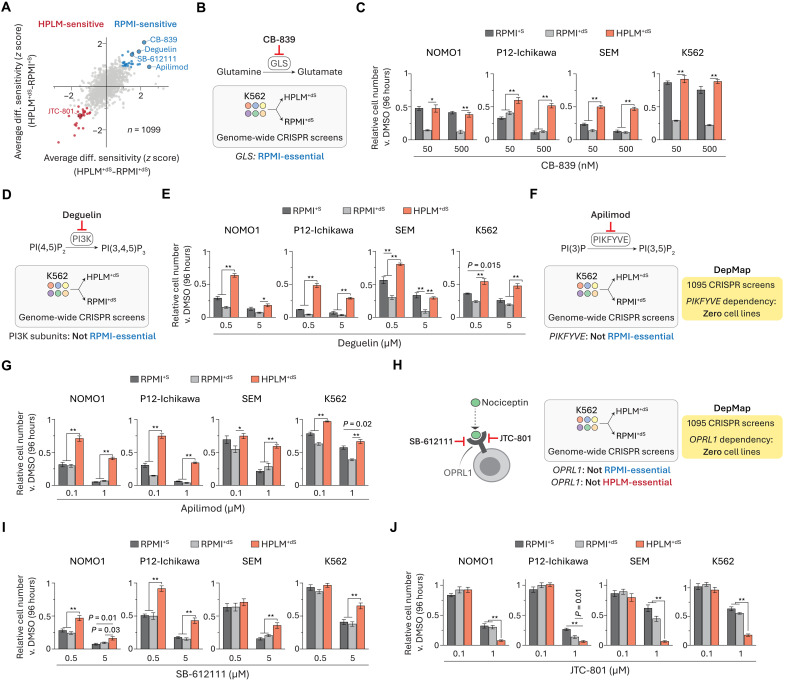
Gene essentiality data suggest that other conditional phenotypes are linked to noncanonical mechanisms. (**A**) Comparison of averaged HPLM^+dS^-RPMI^+dS^ and HPLM^+dS^-RPMI^+S^ phenotypes. (**B**, **D**, **F**, and **H**) Gene essentiality data for canonical targets of CB-839 (B), deguelin (D), apilimod (F), SB-612111 and JTC-801 (H). (**C**, **E**, **G**, **I**, and **J**) Relative growth of cells treated with CB-839 (C), deguelin (E), apilimod (G), SB-612111 (I), or JTC-801 (J) versus DMSO (mean ± SD, *n* = 3, **P < 0.005 and *P < 0.01).

We also used our chemical screen data to examine the set of compounds identified as RPMI-sensitive regardless of FBS supplement (Fig. 10A). The top hit was CB-839, a glutaminase (GLS) inhibitor that has been evaluated in clinical trials for cancer therapy (*87*). Our previously reported CRISPR screens revealed that *GLS* deletion in K562 cells caused differentially stronger effects in RPMI^+dS^ versus HPLM^+dS^ (Fig. 10B) (*27*), a conditional phenotype that we had traced to pyruvate availability and recapitulated as a drug-nutrient interaction by using CB-839. We had also unexpectedly found that this conditional *GLS* essentiality further depended on FBS dialysis, as *GLS*-knockout K562 cells instead showed comparable growth defects in RPMI^+S^ and HPLM^+dS^ despite the 5- to 10-fold lower pyruvate levels provided in 10% FBS relative to HPLM (fig. S11A). Thus, we sought to examine CB-839 sensitivity across our cell line panel. While responses to CB-839 in two cell lines resembled the conditional phenotypes for loss of *GLS* observed in K562 cells, the other two lines showed stronger growth defects in RPMI-based media regardless of FBS dialysis (Fig. 10C and fig. S11B).

The next strongest RPMI-sensitive hits were deguelin, a reported PI3K inhibitor shown to impair cell growth across several cancer types (*88*), and apilimod, a putative phosphatidylinositol 3-phosphate 5-kinase (PIKFYVE) inhibitor that has been tested in lymphoma patients (*89*, *90*). Notably, we did not identify any catalytic (*PIK3C*) or regulatory (*PIK3R*) subunits of PI3K among the nearly 430 RPMI-essential hits from our previous genome-wide CRISPR screens in K562 cells (Fig. 10D). Nonetheless, deguelin sensitivity was markedly stronger in the two RPMI conditions as expected, although the differential effects in RPMI^+S^ versus HPLM^+dS^ were weaker in SEM cells (Fig. 10E and fig. S11C). This suggests that the responses were unlikely linked to PI3K inhibition, although several other possible mechanisms have been proposed to explain deguelin activity as well (*88*). *PIKFYVE* was similarly not among the RPMI-essential hits identified from our prior genome-wide K562 screens (Fig. 10F). Furthermore, *PIKFYVE* has not been defined as a dependency in any of the 1100 cancer cell lines from the DepMap (*91*). When we tested apilimod against our cell line panel, we observed 30 to 60% stronger growth defects in the RPMI-based media as expected, although these effects also varied with FBS dialysis in some cases (Fig. 10G and fig. S11D). Thus, despite reported evidence that apilimod can bind PIKFYVE (*89*, *92*), these results suggest that the observed conditional apilimod sensitivity might be attributed to an alternative mechanism.

Among the remaining RPMI-sensitive hits from our analysis was SB-612111, an established antagonist of the opioid receptor for nociceptin (OPRL1) (*93*). We also identified a distinct OPRL1 antagonist (JTC-801) in the set of HPLM-sensitive hits (*94*). Regardless of these inverse conditional phenotypes, *OPRL1* is not defined as essential for any cell lines catalogued in DepMap, and further, our previous CRISPR screen results suggest that *OPRL1* is not essential for K562 cells grown in HPLM^+dS^ or RPMI^+dS^ (Fig. 10H). As anticipated, SB-612111 treatment more strongly impaired cell growth in the RPMI conditions, whereas JTC-801 elicited stronger growth defects in HPLM^+dS^, with the extent of these differential effects varying by cell line (Fig. 10, I and J, and fig. S11, E and F). These results offer additional evidence that non-oncology drugs can elicit anticancer effects and also demonstrate how conditional phenotypes for drugs that share an otherwise common canonical target can vary. Of note, increasing exogenous folic acid to RPMI-defined levels did not affect JTC-801 sensitivity in HPLM^+dS^ (fig. S11G). In addition, RNA sequencing data across nearly 1500 human cancer lines indicate that *OPRL1* shows low or negligible expression in 70% of the analysis set (*95*), including three cell lines in our panel (fig. S11, G and H).

## DISCUSSION

Here, we have demonstrated a profiling strategy to systematically identify compounds with differential anticancer activity in HPLM versus traditional media. Our screens revealed conditional phenotypes for preclinical compounds and several drugs that have been tested or approved for either cancer therapy or non-oncology uses. We found that conditional treatment responses can further vary with natural intrinsic factors even among a set of just four leukemia cell lines. This suggests that chemical screens in HPLM across expanded cell line panels should make it possible to uncover genomic determinants of drug sensitivity under nutrient conditions with greater relevance to human physiology. Target-based and phenotypic chemical screening in HPLM could also support unbiased drug discovery efforts in noncancer cell types. While we confirmed phenotypes for several hits revealed by our screens, there are many others that should be of interest for future studies.

Using hypothesis-driven rationale, we traced conditional phenotypes for several approved cancer therapies, including antifolates and various nucleoside or base analogs to the availability of nucleotide salvage pathway substrates (hypoxanthine or thymidine) uniquely defined in HPLM or contributed by a standard 10% FBS supplement. These results demonstrate that both the synthetic and serum components of complete media can affect drug activity.

Using an unbiased approach, we also identified a drug-nutrient interaction between BVDU and folic acid, an essential vitamin provided at highly supraphysiologic levels in RPMI and other traditional media. We further delineated BVDU-induced disruptions in folate-dependent nucleotide synthesis based on systematic complementation with hypoxanthine and thymidine. We reason that this approach could be applied to address whether other drugs that target 1C metabolism similarly cause epistatic or additive effects on the folate-dependent nucleotide synthesis pathways. Adjusting the availability of nucleotide salvage pathway substrates and formate led to the differential rescue of growth defects caused by BVDU versus specific genetic knockouts, indicating that comparisons of gene- and drug-nutrient interactions can also offer insights into mechanisms of action. Our results support evidence that TK2 can activate BVDU, suggesting that TK2 expression could perhaps serve as a biomarker for BVDU sensitivity. CRISPR screening revealed genetic contributions to BVDU activity, as we further demonstrated a general strategy to define distinct gene-drug interactions. By integrating our nutrient complementation approaches, CRISPR screens, polar metabolomics, folate profiling, and MS-based activity assays, we ultimately characterized a dual-targeting mechanism to explain the conditional lethality of BVDU. Notably, BVDU-MP shares no structural similarity with any reported MTHFD1/2 inhibitors, which are also instead characterized for activity against MTHFD1/2(CD). Moreover, the anticancer mechanism for BVDU is distinct versus those described for other pyrimidine nucleoside analogs, including FCdR and additional dS-sensitive hits revealed by our screens. These findings also suggest the potential to identify other compounds like BVDU-MP that act against human MTHFD1(S).

Conditional phenotypes that we identified for CB-839 and 5-FU in prior work were broadly replicated across our leukemia cell line panel. However, differential responses to CB-839 in two of the four cell lines further depended on dialysis of the FBS supplement used to prepare RPMI-based media, supporting the notion that a complex interplay of factors can affect CB-839 sensitivity (*26*). We also identified drug-nutrient interactions linked to folic acid for two additional HPLM-sensitive hits (SCH-79797 and TG100-115), each with reported activities in human cells unrelated to folate-dependent processes but that otherwise share bacterial DHFR as a noncanonical target. Thus, our results indicate that both drugs can also disrupt 1C metabolism in human cells, although their relevant molecular target(s) are currently unknown. Of note, metabolite profiling on a more recent lot of the commercial solution initially used for incorporating vitamins into HPLM revealed that working concentrations for biotin and folic acid were more comparable to—although still twofold lower than—respective RPMI-defined concentrations (fig. S12, A and B). Moreover, while the manual HPLM preparation approach that we described easily allows for controlled modifications, it is worth noting that commercial HPLM (Thermo Fisher Scientific) contains RPMI-defined vitamins as initially intended, including folic acid and biotin.

By leveraging CRISPR screen results from our previous work (*27*), we found that canonical targets of other validated hits (apilimod, deguelin, SB-612111, and JTC-801) are encoded by genes that were not identified as conditionally essential, whereas conditional phenotypes for CB-839 and *GLS* knockout were instead more equivalent. For multiple cases, these genes are also not defined as essential across any of the 1100 screens in the DepMap. SB-612111 and JTC-801 share the same canonical opioid receptor target yet induced inverse medium-dependent responses. Together, these data demonstrate the potential for integrating conditional gene essentiality and drug sensitivity profiles either to corroborate putative mechanisms of action or to suggest the possibility that compounds elicit off-target effects.

Conditional lethality, in principle, can be exploited for efforts in anticancer drug discovery and development. Nonetheless, it is important to distinguish the concept of conditional sensitivity from the question of whether a drug elicits anticancer effects. Conditional phenotypes found in our screen results were not—nor expected to be—predictive of either global development phase or reported indications. For example, we confirmed RPMI-sensitive phenotypes for purine analogs (6-TG and DTIC) that have been approved for cancer therapy but nonetheless induced conditional responses, which we traced to hypoxanthine availability. Moreover, in contrast to evaluating how a genetic knockout might affect cell growth, the effects of an otherwise “active” compound can be shaped in many cases by simply adjusting the treatment dose regardless of growth conditions. This critical distinction arguably precludes establishing general correlations between treatment responses found in vitro—regardless of relative modeling capacity—versus those observed in animal models. Collectively, we reason that our conditional profiling strategy could identify drugs that might offer larger therapeutic windows if they induce greater sensitivity in HPLM versus traditional media but not necessarily to uncover “selectively active” drugs based on medium composition–with BVDU the closest example of such a case from our screening datasets. Drug-nutrient interactions may also reveal plasma biomarkers for treatment efficacy and, perhaps, could be further exploited to enhance the effects of RPMI-sensitive drugs through combination therapies with nutrient transport inhibitors or the targeted modulation of systemic metabolites (*96*, *97*).

### Limitations of study

Since we chose to normalize glucose levels between growth conditions, it is possible that if we had used RPMI-based media with the conventional glucose concentration (11.1 mM), we might have also uncovered conditional phenotypes linked to glucose availability. Next, it is unclear why we could not detect product formation from in vitro enzyme assays containing only the substrates for MTHFD1(S). While the further addition of NADPH allowed us to detect both such products and to ultimately find that BVDU-MP affected relative ADP levels, drug-mediated effects on each metabolite evaluated in this multidomain activity assay are difficult to completely reconcile given the relative reversibility of the MTHFD1 reactions and how these may be otherwise influenced in the context of the cytosol. The molecular basis for how BVDU-MP affects MTHFD1(S) remains to be elucidated but could perhaps be addressed in future studies. Last, while our screens and follow-up work demonstrate that natural cell-intrinsic factors can affect conditional lethality, the number of cell lines in our panel does not match the scope of larger drug sensitivity catalogs. This could be addressed by screening expanded panels in future work.

## MATERIALS AND METHODS

Reagents and Resources can be found in the Supplementary Materials.

### Mouse studies

All animal studies were performed under a protocol approved by the Institutional Animal Care and Use Committee at the University of Wisconsin (UW)–Madison (M006660-A01). NSG mice were provided by C. Capitini (UW-Madison). All mice were maintained on a 12-hour light-dark cycle under monitoring by Research Animal Resources and Compliances at UW-Madison. Twelve- to 14-week-old male NSG mice were used for experiments. The remaining methods for mouse studies can be found in the Supplementary Materials.

### Cell lines

The following human cancer cell lines were provided by: K562 and NOMO1, J. Griffin (Dana Farber Cancer Institute); P12-Ichikawa, A. T. Look (Dana Farber Cancer Institute); and SEM, the Cancer Cell Line Encyclopedia (Broad Institute). Cell lines were verified to be free of mycoplasma contamination, and their identities were authenticated by short tandem repeat profiling.

### Cell culture conditions

The following culture media were used in this study (all contained 0.5% penicillin-streptomycin):

1) RPMI^+S^: RPMI 1640, no glucose (Thermo Fisher Scientific) with 5 mM glucose and 10% FBS.

2) RPMI^+dS^: RPMI 1640, no glucose (Thermo Fisher Scientific) with 5 mM glucose and 10% dialyzed FBS.

3) RPMI11^+S^: RPMI 1640 (Thermo Fisher Scientific) with 10% FBS.

4) RPMI11^+2S^: RPMI 1640 (Thermo Fisher Scientific) with 20% FBS.

5) DMEM^+S^: DMEM, high glucose, GlutaMAX (Thermo Fisher Scientific) with 10% FBS.

6) DMEM^+2S^: DMEM, high glucose, GlutaMAX (Thermo Fisher Scientific) with 20% FBS.

7) HPLM^+dS^: Manually prepared HPLM (see table S2) with 10% dialyzed FBS and using RPMI 1640 100× vitamins (Sigma-Aldrich R7256).

8) HPLM^+dS^: Manually prepared HPLM (see table S2) with 0.45 μM folic acid, eight other RPMI-defined vitamins, and 10% dialyzed FBS.

HPLM-based media using either (7) or (8) are distinguished with the shaded colors in Fig. 4, D and J. The basal HPLM formulation in (8) was used across all experiments except the chemical screens and those shown in Fig. 4, C, E, and G, and fig. S4B.

Using SnakeSkin tubing (Thermo Fisher Scientific, PI88244), FBS was dialyzed as previously described (*13*). Before use, all FBS-supplemented media were sterile-filtered using a bottle-top vacuum filter with cellulose acetate membrane, pore size of 0.2 μm (Corning, 430626; or Nalgene, 290-4520). Cultured cells were maintained at 37°C, atmospheric oxygen, and 5% CO_2_.

### High-throughput chemical screens

NOMO1, P12-Ichikawa, and SEM cell lines were each initially maintained in RPMI^+S^. After four to six passages, cells were split and cultured in either RPMI^+S^, RPMI^+dS^, or HPLM^+dS^ for at least four passages before screening in each respective condition. Seeding densities for each cell line were tested and in turn selected to minimize growth rate differences between conditions. For each cell line–medium combination, cells were plated on the same day for high-throughput screening. Cells were seeded in 1536-well white solid bottom polystyrene Greiner plates containing 5 μl of growth medium using a Multidrop Combi Dispenser with a small tube dispensing cassette—the NOMO and SEM lines were plated at 500 cells per well and the P12-Ichikawa line at 1000 cells per well. Within each individual plate, one column was not seeded with cells and served as a background control. After seeding the cells, 23 nl of MIPE 4.1 library compounds [in dimethyl sulfoxide (DMSO)] was added to each plate using a Kalypsys 1536 Pintool dispenser. Bortezomib (final concentration, 2 μM) and DMSO were similarly added to each plate as positive and negative controls for cytotoxicity, respectively. For each screen, MIPE 4.1 compounds were added over an 11-point concentration range from 47 μM to 0.79 nM in threefold dilutions immediately after plating cells. Each plate was covered with a stainless steel gasketed lid to prevent evaporation and then housed in a tissue culture incubator maintained at 37°C, 95% relative humidity, and 5% CO_2_.

Following 48-hour incubation with compound, plate lids were removed, and 3 μl of CellTiter-Glo One (Promega) was added to each well by using a solenoid valve dispenser. Plates were incubated with lids in place for 15 min at room temperature, and then luminescence readings were taken using a ViewLux ultra HTS Microplate Imager (PerkinElmer) with a 2-s exposure time per plate. Data for each compound were normalized as percent viability such that measurements for DMSO and the empty well controls in each plate were defined as 100 and 0%, respectively. Assay statistics were then calculated, and all screened plates had a *Z*-factor greater than 0.4.

### CRISPR modifier screens

#### BVDU dose-responses in flask format

Following at least two passages in RPMI^+S^, K562 cells were pelleted and used to seed a T-75 cell culture flask (Corning, 430641U) at a density of ~150,000 cells/ml in 20 ml of HPLM^+dS^. After 48-hour incubation, pools of 2 million cells were used to seed T-75 culture flasks at a density of 100,000 cells/ml in 20 ml of HPLM^+dS^. Following 1-hour incubation of the seeded flasks, BVDU (final concentrations, 1 μM, 500 nM, 100 nM, or 50 nM) was added to the cells. All flasks, including the untreated controls, contained 0.25% DMSO. After 72-hour treatment, cell density measurements were recorded using a Coulter Counter (Beckman Z2 or Multisizer 4e) with a diameter setting of 8 to 30 μm and used to determine the EC_25_ for a culture flask format relevant to the CRISPR screens. The stock solution of BVDU was prepared at 40 mM in DMSO.

#### Human sgRNA library amplification

pLentiCRISPRv2-Opti plasmid containing a human sgRNA library designed by E. M. Frenkel was provided by the Whitehead Institute Functional Genomics Platform. The genome-wide human sgRNA library contained 98,077 constructs targeting 19,734 protein-coding genes and 208 noncoding RNAs (~5 sgRNAs per target) and 499 total intergenic and nontargeting control sgRNAs. Library plasmid was transformed into *Escherichia coli* Endura electrocompetent cells (Lucigen), plated onto prewarmed LB medium/agar containing carbenicillin (100 μg/ml) in a 245-mm square bioassay dish (Corning, 431111), and incubated for 16 hours at 30°C, yielding ~10^8^ individual transformants—equivalent to ~1000-fold coverage of the theoretical library diversity. Colonies were scraped and pooled in LB medium, and plasmid DNA was extracted using an EndoFree Maxi Kit (QIAGEN).

#### Genome-wide CRISPR screens

To achieve at least 1000-fold coverage of the sgRNA library following antibiotic selection, 300 million K562 cells were seeded at a density of 2.5 × 10^6^ cells/ml in six-well plates containing 2 ml of RPMI11^+S^, polybrene (8 μg/ml), and the pLentiCRISPR-v2-Opti library virus. Spin infection was carried out by centrifugation at 2200 rpm for 45 min at 37°C. After 18-hour incubation, cells were pelleted to remove virus and then reseeded in fresh RPMI11^+S^ for 24 hours. Cells were then pelleted, reseeded to a density of 150,000 cells/ml in RPMI11^+S^ containing puromycin (2 μg/ml; Sigma-Aldrich), and cultured for 72 hours. Following selection, an initial pool of 100 million cells was pelleted and frozen, and another pool of 175 million cells was used to collectively seed each of 14 total 225-cm^2^ rectangular canted neck cell culture flasks (Corning, 431082) at a density of 100,000 cells/ml in 125 ml of HPLM^+dS^. BVDU (final concentration, 100 nM) or DMSO was then added to each of seven flasks such that all cultures contained 0.25% DMSO. Cells were passaged every 72 hours, with fresh BVDU or DMSO added at each passage, and population doublings were tracked by cell density measurements using a Coulter Counter with a diameter setting of 8 to 30 μm. After 15 population doublings, a pool of 100 million cells from each screen was harvested for genomic DNA (gDNA) extraction using the QIAamp DNA Blood Maxi Kit (QIAGEN). Using Ex Taq DNA Polymerase (Takara), sgRNA inserts from each initial and final pool were polymerase chain reaction (PCR)–amplified from 144 μg of gDNA to achieve ~400-fold coverage of the library. The PCR products were then purified and sequenced on a NextSeq 500 (Illumina) (primer sequences are annotated in table S5) to quantify sgRNA abundances in each sample.

### Plasmid construction

All oligonucleotides and gBlock Gene fragments used in this study are described in table S5.

#### Construction of gene knockout plasmids

For each of the following genes, sense and antisense oligonucleotides were annealed and then cloned into Bsm BI–digested pLentiCRISPR-v1: *TYMS*, *TK2*, *ABCC4*, *SHMT2*, and *MTHFD1*.

#### Construction of expression plasmids

The *TK2* and *DHFR* genes were amplified from codon-optimized gBlock gene Fragments [Integrated DNA Technologies (IDT)] using the primers TK2-F/TK2-R and DHFR-F/DHFR-R, respectively, digested with Pac I–Not I, and cloned into pLJC2-Rap2A-3xFLAG to generate pLJC2-TK2-3xFLAG and pLJC2-DHFR-3xFLAG. The *MTHFD1* gene was amplified from pcDNA3_N-DYK_MTHFD1-NES using primers MTHFD1-F/MTHFD1-R, digested with Pac I–Not I, and then cloned into pLJC2-Rap2A-3xFLAG to generate pLJC2-MTHFD1-3xFLAG.

### Lentivirus production

To produce lentivirus, human embryonic kidney (HEK) 293T cells in DMEM^+S^ were cotransfected with the VSV-G envelope plasmid, the Delta-VPR packaging plasmid, and transfer plasmid (either pLJC2, pLentiCRISPR-v1, or pLentiCRISPRv2-Opti backbone or pLenti-PGK-Venus-AkaLuc) using X-tremeGENE 9 Transfection Reagent (Sigma-Aldrich). The medium was exchanged with fresh DMEM^+2S^ 16 hours after transfection, and the virus-containing supernatant was collected at 48 hours posttransfection, passed through a 0.45-μm filter to eliminate cells, and then stored at −80°C.

### Cell line construction

#### Knockout cell lines

To establish *SHMT2*-knockout clonal cell lines, K562 cells were seeded at a density of 500,000 cells/ml in six-well plates containing 2 ml of RPMI11^+S^, polybrene (8 μg/ml), and pLentiCRISPR-v1 lentivirus. Spin infection was carried out by centrifugation at 2200 rpm for 45 min at 37°C. After 16- to 18-hour incubation, cells were pelleted to remove virus and then reseeded into fresh RPMI11^+S^ for 24 hours. Cells were then pelleted and reseeded into RPMI11^+S^ containing puromycin for 72 hours. Following selection, the population was single-cell fluorescence-activated cell sorting (FACS)–sorted into 96-well plates containing RPMI11^+2S^ (BD FACSMelody Cell Sorter). After 1.5 to 2 weeks, clones with the desired knockouts were identified by immunoblotting. To control for infection, a population of K562 cells was similarly selected following transduction with sg*AAVS1*-containing virus. The procedure to establish other knockout cell lines for short-term growth or drug treatment assays was similar except that cells were not FACS-sorted following puromycin selection (5 days postinfection) (see the “Short-term growth and drug treatment assays” section).

#### TK2 cDNA expression cell line

To establish the *TK2* expression cell line, SEM cells were seeded at a density of 500,000 cells/ml in six-well plates containing 2 ml of RPMI11^+S^, polybrene (8 μg/ml), and the pLJC2-TK2-3xFLAG lentivirus. Spin infection, culture medium exchange, and puromycin selection were carried out as described above for the knockout cell lines. Stable expression of *TK2* cDNA was confirmed by immunoblotting.

#### Akaluciferase cDNA expression cell line

To establish the Venus-AkaLuc expression cell line, K562 cells were seeded at a density of 500,000 cells/ml in six-well plates containing 2 ml of RPMI11^+S^, polybrene (8 μg/ml), and the pLenti-PGK-Venus-AkaLuc lentivirus. Spin infection and medium exchange were carried out as described above, and then cells were reseeded into RPMI11^+S^ containing G 418 for 72 hours.

### Cell lysis for immunoblotting

Cells were centrifuged at 250*g* for 5 min, resuspended in 1 ml ice-cold phosphate-buffered saline (PBS), and then centrifuged again at 250*g* for 5 min at 4°C. Cells were then immediately lysed with ice-cold lysis buffer [40 mM tris-HCl (pH 7.4), 1% Triton X-100, 100 mM NaCl, 5 mM MgCl_2_, 1 tablet of EDTA-free protease inhibitor (Roche 11580800; per 25-ml buffer), and 1 tablet of PhosStop phosphatase inhibitor (Roche 04906845001; per 10-ml buffer)]. Cell lysates were cleared by centrifugation at 21,130*g* for 10 min at 4°C and quantified for protein concentration using an albumin standard (Thermo Fisher Scientific, 23209) and Bradford reagent (Bio-Rad, 5000006). Cell lysate samples were normalized for protein content, denatured upon the addition of 5× sample buffer (Thermo Fisher Scientific, 39000), resolved by 12% SDS–polyacrylamide gel electrophoresis (SDS-PAGE), and transferred to a polyvinyl difluoride membrane (Millipore IPVH07850). Membranes were blocked with 5% nonfat dry milk in Tris Buffered Saline with Tween (TBST) for 1 hour at room temperature, and then incubated with primary antibodies in 5% nonfat dry milk in TBST overnight at 4°C. Primary antibodies to the following proteins were used at indicated dilutions: glyceraldehyde phosphate dehydrogenase (1:1000), RAPTOR (1:1000), TYMS (1:1000), SHMT2 (1:500), MTHFD1 (1:250), TK2 (1:100), and Beta-Actin (ACTB) (1:1000).

Membranes were washed with TBST three times for 5 min each and then incubated with species-specific horseradish peroxidase–conjugated secondary antibody (1:3000) in 5% nonfat dry milk for 1 hour at room temperature. Membranes were washed again with TBST three times for 5 min each and then visualized with chemiluminescent substrate (Thermo Fisher Scientific) on a LI-COR Odyssey FC.

### Cellular thermal shift assay

Following at least one passage in HPLM^+dS^, 4 million K562 cells were pelleted and seeded to a density of 1 million cells/ml in six-well plates containing 4 ml of media. After 5 min of incubation of seeded plates, BVDU, FCdR, or MTX (final concentration, 1 μM) was added to cells, and then plates were gently shaken for 2 min. All wells, including the untreated controls, contained 0.25% DMSO. Following 2-hour treatment, cells were washed once with ice-cold PBS, pelleted, and then resuspended in 100 μl of ice-cold PBS containing EDTA-free protease inhibitor (Roche). Cells were transferred to a PCR-strip tube and heated for 3 min using a Mastercycler Nexus X2 thermal cycler (Eppendorf). After heating, samples were snap-frozen with liquid nitrogen and then evenly thawed by transferring strip tubes to a CoolRack XT (Corning) placed in a dry block heater held at 25°C. Following two additional freeze-thaw cycles with gentle pulse vortexing after each thaw, cell lysates were cleared by centrifugation at 21,130 *g* for 20 min at 4°C. Cell lysate samples were normalized for protein content and denatured upon the addition of 5× sample buffer.

The procedure used to determine the melting temperature for TYMS was similar to that described above with the following modifications:

1) Only K562 cells in HPLM^+dS^ with 0.25% DMSO were tested.

2) Cells were heated over a gradient of temperatures, either 37.1° to 49°C or 49.1° to 61.1°C, set using the Nexus X2.

### Short-term growth and drug treatment assays

#### Cell line panel: Drug treatments

Following at least two passages in RPMI^+S^, cells were pelleted and used to seed T-25 cell culture flasks (Corning, 430639) containing 12 ml of RPMI^+S^, RPMI^+dS^, or HPLM^+dS^. K562 cells were seeded to a density of ~150,000 cells/ml, while the remaining cell lines (NOMO1, P12-Ichikawa, and SEM) were seeded at densities between 400,000 and 500,000 cells/ml. After 48-hour incubation, cells were pelleted and resuspended to densities of either 1 million cells/ml (K562, NOMO1, and P12-Ichikawa) or 2 million cells/ml (SEM) in the respective parent culture medium. From each resuspension, either 80,000 (K562, NOMO1, and P12-Ichikawa) or 160,000 (SEM) total cells were seeded in each of three replicate wells (per dosing concentration) in six-well plates containing 4 ml of the appropriate culture medium. Following 1-hour incubation of seeded plates, compounds were added at specified doses, and then plates were gently shaken for 2 min. All wells, including the untreated controls, contained 0.25% DMSO. After 96-hour treatment, cell density measurements were recorded using a Coulter Counter with a diameter setting of 8 to 30 μm. For each cell line compound combination, assays were performed across conditions in the same experiment.

#### Engineered K562 cell lines

The short-term growth and drug treatment assays with *SHMT2-*knockout K562 clonal cells were identical to those described above. For the *TYMS*-, *TK2-*, *ABCC4-*, and *MTHFD1*-knockout K562 cell lines, FACS sorting was not performed following puromycin selection. Instead, 3 million cells were pelleted and resuspended to a density of 250,000 cells/ml in 12 ml of RPMI^+S^. After 48-hour incubation (7 days postinfection), 3.5 million cells were pelleted and resuspended to a density of ~300,000 cells/ml in 12 ml of HPLM^+dS^. Following 48-hour incubation (9 days postinfection), pools of cells were pelleted and seeded for the 96-hour growth step—with drug treatment when appropriate—as described above, with cell density measurements ultimately recorded at 13 days postinfection. To control for infection and all ensuing steps over the course of these 2-week experiments, a population of K562 cells was transduced with sgAAVS1-containing virus, selected, passaged, and assayed parallel to unsorted genetic knockout populations.

The short-term treatment assay procedure using RPMI- or HPLM-based derivatives was identical to that above with minor modifications:

1) The following were added to the appropriate complete media only for the final 96-hour step of the assay: hypoxanthine, uridine, thymidine, cytidine, deoxyuridine, and deoxycytidine. Stock solutions of each individual component were prepared at 10 mM in either water or 0.2 M HCl (hypoxanthine and uridine).

2) Modified concentrations of amino acids, salt ions, and vitamins were incorporated at the preceding medium-specific 48-hour passage step. Stock solutions of individual vitamins were prepared as follows: biotin (8.2 mM in 20 mM NaOH), folic acid (450 μM in 20 mM NaOH), and 5-mTHF (1 mM in 50 mM NaOH).

#### K562 cells: For cellular folates

Following at least two passages in RPMI^+S^, K562 cells were pelleted and used to seed T-25 cell culture flasks at a density of ~150,000 cells/ml in 12 ml of the appropriate HPLM-based medium. After 48-hour incubation, cells were pelleted and resuspended to a density 1 million cells/ml in the respective passage medium. From each resuspension, 500,000 total cells were seeded in each of three T-75 cell culture flasks containing 25 ml of the appropriate HPLM-based medium. After 1- hour incubation of seeded flasks, compounds were added at specified doses and then treated for 96 hours. All flasks, including the untreated controls, contained 0.25% DMSO.

### Metabolite profiling and quantification of metabolite abundance

Liquid chromatography–mass spectrometry (LC-MS) analyses were performed on a QExactive HF benchtop orbitrap mass spectrometer equipped with an Ion Max API source and HESI II probe, coupled to a Vanquish Horizon UHPLC system (Thermo Fisher Scientific). External mass calibration was performed using positive and negative polarity standard calibration mixtures every 7 days. Acetonitrile was hypergrade for LC-MS (Millipore Sigma), and all other solvents were Optima LC-MS grade (Thermo Fisher Scientific).

#### Cells: Polar metabolites

At the conclusion of short-term growth or treatment assays, a 500-μl aliquot from each well was used to measure cell number and volume via Coulter Counter with a diameter setting of 8 to 30 μm, and the remaining cells were centrifuged at 250*g* for 5 min, resuspended in 1 ml of ice-cold 0.9% sterile NaCl (Growcells, MSDW1000), and again centrifuged at 250*g* for 5 min at 4°C. Metabolites were extracted in 1 ml of ice-cold 80% methanol containing 500 nM internal amino acid standards. Following a 10-min vortex and centrifugation at 21,130*g* for 3 min at 4°C, samples were dried under nitrogen gas. Dried samples were stored at −80°C and resuspended in 100 μl of water. After a 10-min vortex and centrifugation at 21,130*g* for 10 min at 4°C, 2.5 μl from each cell sample was injected onto a ZIC-pHILIC 2.1 mm by 150 mm analytical column equipped with a 2.1 mm by 20 mm guard column (both were 5-μm particle size, Millipore Sigma). Buffer A was 20 mM ammonium carbonate and 40 mM ammonium hydroxide; buffer B was acetonitrile. The chromatographic gradient was run at a flow rate of 0.15 ml/min as follows: 0 to 20 min: linear gradient from 80 to 20% B; 20 to 20.5 min: linear gradient from 20 to 80% B; 20.5 to 28 min: hold at 80% B. The mass spectrometer was operated in full-scan, polarity-switching mode with the spray voltage set to 3.0 kV, the heated capillary held at 275°C, and the HESI probe held at 350°C. The sheath gas flow rate was set to 40 units, the auxiliary gas flow was set to 15 units, and the sweep gas flow was set to 1 unit. The MS data acquisition in positive mode was performed in a range of 50 to 750 mass/charge ratio (*m/z*), with the resolution set to 120,000, the Automated Gain Control (AGC) target at 10^6^, and the maximum integration time at 20 ms. The settings in negative mode were the same except that the range was instead 70 to 1000 *m/z*.

For the highly targeted analysis of several metabolites, additional tSIM (targeted selected ion monitoring) scans were added with the following settings: resolution set to 120,000, an AGC target of 10^5^, maximum integration time of 200 ms, and isolation window of 1.0 *m/z*. The target masses, each in negative ionization mode, were 87.0088 (pyruvate), 321.0493 (dTMP), 347.0398 (IMP), and 480.982 (dTTP). For BVDU-treated samples, identical tSIM scans in negative ionization mode were added for the following target masses: 330.9935 (BVDU) and 410.9598 (BVDU-MP). For FCdR-treated samples, an identical tSIM scan in negative ionization mode was added for the following target mass: 325.0243 (FdUMP).

#### Media: Relative folic acid availability

Samples of HPLM-based media containing either 0.45 or 2.27 μM folic acid were snap-frozen in liquid nitrogen and stored at −80°C before inoculation of short-term BVDU treatment assays. At the conclusion of these assays, a 500-μl aliquot from each well was collected and centrifuged at 250*g* for 5 min. Metabolites were extracted from both the resulting supernatants and the initial samples by diluting 1:40 in a solution of 50:30:20 methanol:acetonitrile:water (MeOH:ACN:H_2_O) containing 500 nM internal amino acid standards. Following a 10-min vortex and centrifugation at 21,130*g* for 5 min at 4°C, 2 μl of each sample was injected for analysis as described above for profiling cell samples. For the highly targeted analysis of folic acid, an additional tSIM scan was added with the same settings described for cell samples, except that the scan was run in positive ionization mode and the target mass was 442.1470.

#### Comparison of vitamin abundances

To extract metabolites from basal RPMI (Thermo Fisher Scientific, 11879), samples were diluted 1:10 into 50:30:20 MeOH:ACN:H_2_O containing 500 nM internal amino acid standards. For RPMI 1640 100× vitamins solution (Sigma-Aldrich, R7256, lots RNBB7627 and RNBK1269), aliquots were first diluted 1:10 in water and vortexed for 1 min at 4°C, and then metabolites were extracted by further diluting 1:10 into 50:30:20 MeOH:ACN:H_2_O containing 500 nM internal amino acid standards. Following a 10-min vortex and centrifugation at 21,130*g* for 5 min at 4°C, 2 μl of each sample was injected for analysis as described above for profiling cell samples. For the highly targeted analysis of biotin, an additional tSIM scan was added with the same settings described for cell samples, except that the scan was run in positive ionization mode and the target mass was 245.0954. Of note, RPMI also contains 3.69 nM vitamin B12, which could not be detected by the profiling method.

#### Fetal bovine serum

To extract metabolites from untreated and dialyzed FBS, samples were diluted 1:40 into 50:30:20 MeOH:ACN:H_2_O containing 500 nM internal amino acid standards. Following a 10-min vortex and centrifugation at 21,130*g* for 3 min at 4°C, samples were dried under nitrogen gas. Dried samples were stored at −80°C and resuspended in 100 μl of water. After a 10-min vortex and centrifugation at 21,130*g* for 10 min at 4°C, 2 μl of each sample was injected for analysis as described above for profiling cell samples. For the highly targeted analysis of pyruvate, an additional tSIM scan was added with the same settings described for cell samples.

#### Plasma

To extract metabolites from mouse plasma, samples were thawed on ice and then diluted 1:10 into 50:30:20 MeOH:ACN:H_2_O containing 500 nM internal amino acid standards. Following a 10-min vortex and centrifugation at 21,130*g* for 5 min at 4°C, 2.5 μl of each sample was injected for analysis as described above for profiling cell samples. For the highly targeted analysis of BVDU, an additional tSIM scan was added with the same settings described for cell samples.

#### Cells: Folates

At the conclusion of short-term treatment assays, a 500-μl aliquot from each flask was used to measure cell number and volume via Coulter Counter with a diameter setting of 8 to 30 μm. From each flask, 3 million cells were then centrifuged at 250*g* for 5 min, resuspended in 1 ml of ice-cold 0.9% sterile NaCl, and again centrifuged at 250*g* for 5 min at 4°C. The ensuing protocol for folate extraction was adapted from others described elsewhere (*71*, *98*). Metabolites were extracted in 1 ml of ice-cold 80% methanol containing 2.5 mM sodium ascorbate, 25 mM ammonium acetate (pH 7), and 100 μm of aminopterin. Following a 10-min vortex and centrifugation at 21,130*g* for 10 min at 4°C, samples were dried under nitrogen gas. Dried samples were stored at −80°C and reconstituted in 400 μl of ice-cold resuspension buffer [50 mM K_2_HPO_4_ (pH 7), 30 mM ascorbic acid, and 0.5% 2-mercaptoethanol]. Following a 10-min vortex and centrifugation at 21,130*g* for 10 min at 4°C, resulting supernatants were transferred to fresh tubes with 25 μl of charcoal-treated rat serum and then gently shaken at 300 rpm for 2 hours at 37°C using a Thermomixer C (Eppendorf). Sample pH was then adjusted to 4 with 15 μl of 20% formic acid before loading onto conditioned SPE Bond-Elute pH columns (Agilent, 14102062) at 4°C. After washing with 1 ml of aqueous buffer [25 mM ammonium acetate (pH 4) and 30 mM ascorbic acid], samples were eluted in 400 μl of 50% methanol containing 30 mM ammonium acetate (pH 7) and 0.5% 2-mercaptoethanol and then dried under nitrogen gas. Dried samples were resuspended in 50 μl of water. After a 10-min vortex and centrifugation at 21,130*g* for 10 min at 4°C, 5 μl of each sample was injected for analysis as described above for profiling cell samples. For the highly targeted analysis of folate species, additional tSIM scans were added with the same settings described for cell samples, except that the scans were run in positive ionization mode and the target masses were 446.1783 (THF), 460.1939 (5-mTHF), and 474.1732 (10-formyl-THF). Peaks corresponding to 5-formyl-THF and 10-formyl-THF were distinguished on the basis of retention time (see table S3).

To generate charcoal-treated rat serum for cleaving polyglutamate tails from intracellular folates, 250 mg of activated charcoal (Sigma-Aldrich, C9157) was added to 5 ml of rat serum (Sigma-Aldrich, R9759) and then incubated head-over-head for 3 hours at 4°C. After centrifugation at 1500*g* for 10 min at 4°C, supernatants were collected and stored at −20°C. To condition SPE Bond-Elute columns, 1 ml of LC-MS–grade methanol and 1 ml of aqueous buffer were sequentially loaded.

#### Enzyme activity assay evaluation

For detection of metabolites from DHFR, MTHFD1(CD), MTHFD1(S), and multidomain MTHFD1 activity assays, reaction mixtures were extracted (see the “Enzyme activity assays” section), and 5 μl of each sample was injected for analysis as described above for profiling cell samples but using the following chromatographic gradient: 0 to 10 min: linear gradient from 80 to 20% B; 10 to 10.5 min: linear gradient from 20 to 80% B; 10.5 to 17.5 min: hold at 80% B. For the highly targeted analysis of reaction components in activity assays, additional tSIM scans were respectively added with the same settings described for cell samples, except that the target masses were 426.0221 (ADP), 474.1732 (10-formyl-THF, positive ionization mode), and 742.0682 (NADP^+^).

#### Synthesis of BVDU monophosphate

For isolation of BVDU-MP from in vitro TK2 reactions, the twice-dried samples were resuspended in 100 μl of water (see the “Enzyme activity assays” section). Following a 10-min vortex and centrifugation at 21,130*g* for 3 min at 4°C, 5-μl aliquots were injected onto the LC-MS with the same settings and chromatographic gradient described above for profiling cell samples. On the basis of an empirically determined retention time of ~6.6 min for BVDU-MP, fractions from eight successive sample injections were collected from 5 to 8 min by disconnecting the viper fitting that feeds into the MS. Fractions were dried under nitrogen gas and stored at −80°C. Dried samples were reconstituted in 100 μl of water. Following a 10-min vortex and centrifugation at 21,130*g* for 3 min at 4°C, the supernatants were pooled and then dried under nitrogen gas. The dried sample was resuspended in 100 μl of water and stored at −80°C. Purified BVDU-MP and a 100 μM stock solution of NMP chemical standards (AMP, CMP, GMP, IMP, dTMP, and UMP) were diluted 1:10 in water containing internal amino acid standards. Following a 10-min vortex and centrifugation at 21,130*g* for 3 min at 4°C, 5 μl of each sample was injected for analysis. The concentration of BVDU-MP was estimated as the average of normalized peak areas across the NMPs.

#### Identification and quantification

Metabolite identification and quantification were performed with XCalibur version 4.1 (Thermo Fisher Scientific) using a 10–parts per million mass accuracy window and 0.5-min retention time window. To confirm metabolite identities and to enable quantification when desired, a manually constructed library of chemical standards was used. Standards were validated by LC-MS to confirm that they generated robust peaks at the expected *m/z* ratio, and stock solutions were stored in pooled format at −80°C. On the day of a given queue, stock solutions were diluted 1:10 in either water (cell samples) or 50:30:20 MeOH:ACN:H_2_O (remaining samples) containing 500 nM internal amino acid standards, then vortexed, and centrifuged as described for biological samples. Aminopterin served as the internal standard for profiling cellular folates. For those metabolites lacking a standard, peak identification was restricted to high confidence peak assignments. See table S3.

Given that metabolite extraction protocols differed by sample type, the internal standard concentrations in processed samples for polar metabolites varied: chemical standards (450 nM), basal RPMI and RPMI 100X vitamins solution (450 nM), media and serum samples (487.5 nM), and cell samples (5 μM). Therefore, the raw peak areas of internal standards within each sample of a given batch were first normalized to account for these differences. Metabolite quantification was performed as described elsewhere. For the final concentrations of chemical standards used to quantitate specific metabolites, see table S3.

### Synthesis of 10-formyl-tetrahydrofolate

To synthesize 10-formyl-THF, a reported protocol was adapted (*99*). Reactions containing 20 mM 5,10-me^+^-THF (Schircks, 16.230) and 70 mM 2-mercaptoethanol were carried out in assay buffer [100 mM tris-HCl (pH 8.5)] in a total volume of 100 μl. After a 1-min vortex at 37°C, reactions were incubated for 1 hour at 25°C. Assuming complete substrate conversion, aliquots of 20 mM 10-formyl-THF were then stored at −80°C. Stock solution of 5,10-me^+^-THF was prepared at 100 mM in DMSO. Tris-HCl buffer was prepared in LC-MS–grade water and pH adjusted using KOH.

### Expression and immunoprecipitation of recombinant proteins

For isolation of recombinant proteins, 4 million HEK293T cells were plated in 15-cm culture dishes containing DMEM^+S^. After 24-hour incubation, cells were transfected with 15 μg of pLJC2 constructs harboring TK2-3xFLAG, DHFR-3xFLAG, or MTHFD1-3xFLAG as described elsewhere. Following an additional 48-hour incubation, cells were rinsed once with ice-cold PBS and then immediately lysed in ice-cold lysis buffer (see the “Cell lysis for immunoblotting” section). Cell lysates were cleared by centrifugation at 21,130*g* for 10 min at 4°C. For anti-FLAG immunoprecipitation, FLAG-M2 affinity gel (Sigma-Aldrich) was washed three times in lysis buffer, and then 400 μl of a 50:50 affinity gel slurry was added to a pool of clarified lysates collected from either 5 (MTHFD1-3xFLAG) or 10 (TK2-3xFLAG and DHFR-3xFLAG) individual 15-cm culture dishes and incubated with rotation for 3 hours at 4°C. Following immunoprecipitation, the beads were washed twice in lysis buffer and then four times with lysis buffer containing 500 mM NaCl. Recombinant protein was then eluted in lysis buffer containing 3x-FLAG peptide (500 μg/ml; Sigma-Aldrich) for 1 hour with rotation at 4°C. The eluent was isolated by centrifugation at 100*g* for 4 min at 4°C (Bio-Rad, 732-6204), buffer-exchanged (Amicon Ultra 10-kDa molecular weight cutoff (MWCO) UFC501024, TK2-3xFLAG, and DHFR-3xFLAG; 30-kDa MWCO UFC503024 and MTHFD1-3xFLAG) against 20 volumes of storage buffer [40 mM tris-HCl (pH 7.5), 100 mM NaCl, and 2 mM dithiothreitol (DTT)], mixed with glycerol [final concentration, 15% (v/v)], snap-frozen with liquid nitrogen, and stored at −80°C. Protein samples were quantified using an albumin standard and Bradford reagent. Purified proteins were denatured upon the addition of 5× sample buffer and resolved by 12% SDS-PAGE.

### Enzyme activity assays

For all enzyme reactions, the assay buffer was 40 mM tris-HCl (pH 7.4), 5 mM Na_2_HPO_4_, 5 mM MgCl_2_, 2 mM DTT, and 100 μM NaCl.

#### TK2: For synthesis of BVDU monophosphate

To synthesize BVDU-MP, 10 parallel reactions of purified recombinant TK2 (10 to 20 nM) with ATP (2 mM) and BVDU (2 mM) in assay buffer were carried out in PCR strip tubes (100 μl) for 12 hours at 37°C using a Nexus X2 thermal cycler. After pooling the reactions, metabolites were extracted by transferring 330-μl aliquots to fresh tubes containing 770 μl of 50:30:20 MeOH:ACN:H_2_O. After a 10-min vortex and centrifugation at 21,130*g* for 3 min at 4°C, supernatants were transferred to fresh tubes and dried under nitrogen gas. Dried samples were resuspended in 125 μl of water, and after a 10-min vortex and centrifugation at 21,130*g* for 3 min at 4°C, supernatants were pooled and dried under nitrogen gas. For isolation of purified BVDU-MP, see the “Metabolite profiling and quantification of metabolite abundance” section.

#### Dihydrofolate reductase

Reactions of recombinant DHFR (20 to 40 nM) with NADPH (100 μM), DHF (100 μM), and either MTX or BVDU-MP (10 μM) in assay buffer were carried out at 37°C in a total volume of 50 μl. Following 5-min incubation, a 30-μl aliquot of the reaction was removed and immediately added to 70 μl of ice-cold MeOH:ACN:H_2_O containing 500 nM internal amino acid standards for metabolite extraction. Samples were then vortexed for 5 min and centrifuged at 21,130*g* for 1 min at 4°C.

NADP^+^ levels generated in each reaction were evaluated by LC-MS analysis of extracted samples. An identically prepared extraction sample containing only the two DHFR substrates was used to correct for background NADP^+^. Using a NADP^+^ chemical standard (10 μM), we determined that reactions without MTX achieved ~40 to 45% turnover.

#### MTHFD1(CD)

Reactions of recombinant MTHFD1 (40 to 60 nM) with NADPH (100 μM), 10-formyl-THF (100 μM), and either LY-345899 or BVDU-MP (10 μM) in assay buffer were carried out at 37°C in a total volume of 50 μl. Following 15-min incubation, a 30-μl aliquot of the reaction was removed and immediately added to 70 μl of ice-cold MeOH:ACN:H_2_O containing 500 nM internal amino acid standards for metabolite extraction. After a 5-min vortex, samples were centrifuged at 21,130*g* for 1 min at 4°C.

NADPH, NADP^+^, and 10-formyl-THF levels from each reaction were evaluated by LC-MS analysis of extracted samples. An identically prepared sample containing only the MTHFD1(CD) substrates was used to correct for background NADP^+^. Using a NADP^+^ chemical standard (10 μM), we determined that reaction samples without LY-345899 achieved ~35 to 40% turnover.

#### MTHFD1(S)

Reactions of purified recombinant MTHFD1 (40 to 60 nM) with ATP (100 μM), THF (100 μM), and formate (1 mM) in assay buffer were carried out at 37°C in a total volume of 50 μl. Following 15-min incubation, a 30-μl aliquot of the reaction was removed and immediately added to 70 μl of ice-cold MeOH:ACN:H_2_O containing 500 nM internal amino acid standards for metabolite extraction. After a 5-min vortex, samples were centrifuged at 21,130*g* for 1 min at 4°C. ADP and 10-formyl-THF levels in each reaction were evaluated by LC-MS analysis of extracted samples. However, by evaluating an identically prepared sample containing only the MTHFD1(S) substrates to correct for background ADP and 10-formyl-THF, we found that synthesis of each product was negligible.

#### Multidomain MTHFD1

Reactions of recombinant MTHFD1 (40 to 60 nM) with ATP (100 μM), THF (100 μM), formate (1 mM), NADPH (100 μM), and a non-endogenous compound (10 μM; LY-345899, BVDU-MP, BVDU, or MTX) in assay buffer were carried out at 37°C in a total volume of 50 μl. Following 15-min incubation, a 30-μl aliquot of the reaction was removed and immediately added to 70 μl of ice-cold MeOH:ACN:H_2_O containing 500 nM internal amino acid standards for metabolite extraction. After a 5-min vortex, samples were centrifuged at 21,130*g* for 1 min at 4°C.

Abundances of the following reaction components were evaluated by LC-MS analysis of extracted samples: ATP, ADP, 10-formyl-THF, NADPH, and NADP^+^. An identically prepared sample containing only the four substrates was used to correct for background ADP and NADP^+^.

Stock solutions of compounds used in enzyme activity assays were prepared as follows: ATP (20 mM in water), THF (100 mM in water), ammonium formate (100 mM in water), NADPH (20 mM in 10 mM NaOH), DHF (20 mM in 500 mM NaOH), MTX (40 mM in DMSO), LY-345899 (40 mM in DMSO), and BVDU (40 mM in DMSO).

### Quantification and statistical analysis

*P* values to compare cell density measurements and relative metabolite levels were determined using a two-tailed Welch’s *t* test. *P* values to compare bioluminescence imaging (BLI) signal measurements were determined using a Kolmogorov-Smirnov test. The exact value of *n* and the definition of center and precision measures are provided in associated figure legends. Bar graphs were prepared in GraphPad Prism 9; remaining plots and heatmaps were prepared in R. All instances of reported replicates refer to *n* biological replicates. Quantification and statistical analysis used for high-throughput chemical screens and genome-wide CRISPR screens can be found in the Supplementary Materials.

## Supplementary Materials

### The PDF file includes:

Reagents and Resources Supplementary Methods Figs. S1 to S12 Legends for tables S1 to S5 References
