# Supplementary material for: Conditional lethality profiling reveals anticancer mechanisms of action and drug-nutrient interactions
Source: Sci Adv. 2024 Oct 4;10(40):eadq3591. doi: 10.1126/sciadv.adq3591 (PMC11451515; doi:10.1126/sciadv.adq3591)
Supplement: Supplementary file 1 — Reagents and Resources Supplementary Methods Figs. S1 to S12 Legends for tables S1 to S5 References [file sciadv.adq3591_sm.pdf]

Science Advances
AAAS

Supplementary Materials for

Conditional lethality profiling reveals anticancer mechanisms of action and drug-nutrient interactions

Kyle M. Flickinger *et al.*

Corresponding author: Jason R. Cantor, jcantor@morgridge.org

Sci. Adv. **10**, eadq3591 (2024)
DOI: 10.1126/sciadv.adq3591

The PDF file includes:

Reagents and Resources
Supplementary Methods
Figs. S1 to S12
Legends for tables S1 to S5
References

Other Supplementary Materials for this manuscript include the following:

Tables S1 to S5

Reagents and Resources

REAGENT or RESOURCE	SOURCE	IDENTIFIER
Antibodies		
Rabbit monoclonal anti-TYMS (D26G11)	Cell Signaling Technology	Cat #: 5449<br>RRID: AB_10694549
Rabbit monoclonal anti-SHMT2 (E7F4Q)	Cell Signaling Technology	Cat #: 33443<br>RRID: N/A
Mouse monoclonal anti-MTHFD1 (A-8)	Santa Cruz Biotechnology	Cat #: sc-271412<br>RRID: AB_10611082
Rabbit polyclonal anti-RAPTOR	EMD Millipore	Cat #: 09-217<br>RRID: AB_612103
Mouse monoclonal anti-GAPDH (0411)	Santa Cruz Biotechnology	Cat #: sc-47724<br>RRID: AB_627678
Rabbit polyclonal anti-TK2	Sigma-Aldrich	Cat #: HPA041162<br>RRID: AB_10961178
Mouse monoclonal anti-β-Actin	Cell Signaling Technology	Cat #: 3700<br>RRID: AB_2242334
Horse anti-Mouse IgG HRP	Cell Signaling Technology	Cat #: 7076<br>RRID: AB_330924
Goat anti-Rabbit IgG HRP	Cell Signaling Technology	Cat #: 7074<br>RRID: AB_2099233
Bacterial and Virus Strains		
Endura ElectroCompetent Cells	Lucigen	Cat #: 60242
XL10-Gold Ultracompetent Cells	Agilent	Cat #: 200315
Chemicals, Peptides, and Recombinant Proteins		
3X FLAG Peptide	Sigma-Aldrich	Cat #: F4799
Anti-FLAG M2 Affinity Gel	Sigma-Aldrich	Cat #: A2220
Mixture of amino acid standards for metabolomics	Cambridge Isotope Laboratories	Cat #: MSK-A2-1.2
Defined synthetic medium components	Multiple	See table S2
RPMI 1640 100X Vitamins	Sigma-Aldrich	Cat #: R7256

Dacarbazine	Sigma-Aldrich	Cat #: D2390
6-Thioguanine	Sigma-Aldrich	Cat #: A4882
Hypoxanthine	Sigma-Aldrich	Cat #: H9377
Uridine	Sigma-Aldrich	Cat #: U3003
Thymidine	Sigma-Aldrich	Cat #: T1895
Cytidine	Sigma-Aldrich	Cat #: C4654
Deoxycytidine	Sigma-Aldrich	Cat #: D3897
Deoxyuridine	Sigma-Aldrich	Cat #: D5412
Fluorodeoxycytidine (FCdR)	Sigma-Aldrich	Cat #: F5307
Methotrexate (MTX)	Sigma-Aldrich	Cat #: M8407
5-Fluorouracil (5-FU)	Sigma-Aldrich	Cat #: F6627
Brivudine (BVDU)	Santa Cruz Biotechnology	Cat #: sc-205607
Biotin	Sigma-Aldrich	Cat #: B4501
Folic acid	Sigma-Aldrich	Cat #: F8758
5-methyl-tetrahydrofolate (5-mTHF)	Schircks Laboratories	Cat #: 16.236
Adenosine triphosphate (ATP)	Sigma-Aldrich	Cat #: A0752
Adenosine diphosphate (ADP)	Sigma-Aldrich	Cat #: A2754
Ammonium formate	Sigma-Aldrich	Cat #: 70221
Brivudine monophosphate (BVDU-MP)	This paper	N/A
Dihydrofolate (DHF)	Sigma-Aldrich	Cat #: D7006
NADPH	Sigma-Aldrich	Cat #: N7505
$NADP^+$	Sigma-Aldrich	Cat #: N5755
5,10-methynyl-tetrahydrofolate (5,10-me^+THF)	Schircks Laboratories	Cat #: 16.230
10-formyl-tetrahydrofolate (10-formyl-THF)	This paper	N/A

Tetrahydrofolate (THF)	Schircks Laboratories	Cat #: 16.208
LY-345899	Sigma-Aldrich	Cat #: SML3497
Ammonium formate	Sigma-Aldrich	Cat #: 70221
SCH-79797	Santa Cruz Biotechnology	Cat #: sc-203693
TG100-115	Advanced ChemBlocks	Cat #: Q65794
CB-839	Sigma-Aldrich	Cat #: 5337170001
Deguelin	Selleck	Cat #: S8132
Apilimod	Advanced ChemBlocks	Cat #: O33822
SB-612111	Sigma-Aldrich	Cat #: SML0571
JTC-801	Selleck	Cat #: S2722
Sodium ascorbate	Sigma-Aldrich	Cat #: PHR1279
Aminopterin	Sigma-Aldrich	Cat #: A3411
Ascorbic acid	Sigma-Aldrich	Cat #: A4544
Rat Serum	Sigma-Aldrich	Cat #: R9759
Activated charcoal	Sigma-Aldrich	Cat #: C9157
5-formyl-tetrahydrofolate (5-formyl-THF)	Schircks Laboratories	Cat #: 16.220
RPMI 1640, no glucose	Thermo Fisher	Cat #: 11879020
RPMI 1640	Thermo Fisher	Cat #: 11875093
DMEM, high glucose, GlutaMAX	Thermo Fisher	Cat #: 10566024
Glucose	Thermo Fisher	Cat #: 15023021
Penicillin-Streptomycin	Thermo Fisher	Cat #: 15140122
Fetal Bovine Serum (FBS), Heat Inactivated	Thermo Fisher	Cat #: 16140071
X-tremeGENE 9 DNA Transfection Reagent	Sigma-Aldrich	Cat #: 6365779001
TaKaRa Ex Taq DNA Polymerase	TaKaRa	Cat #: RR001A

Puromycin dihydrochloride	Sigma-Aldrich	Cat #: P7255
G 418 disulfate salt solution	Sigma-Aldrich	Cat #: G8168
TokeOni (AkaLumine-HCl)	Sigma-Aldrich	Cat #: 808350
Critical Commercial Assays		
QIAamp DNA Blood Maxi Kit	QIAGEN	Cat #: 51194
EndoFree Plasmid Maxi Kit	QIAGEN	Cat #: 12362
Deposited Data		
DepMap chemical screens, Culture conditions	(*18*)	https://depmap.org/repurposing/
GDSC chemical screens, Culture conditions	(*14*, *15*)	Basal: *(15)* Table S1<br>Serum: *(14)* Methods
CTD2 chemical screens, Culture conditions	(*17*)	*(17)* Supplemental Data Set 1
Conditional gene essentiality data in K562 cells	(*27*)	*(27)* Tables S1 and S3
Probability of dependency data from genome-wide CRISPR screens in K562 cells	(*27*)	*(27)* Table S1
PIKFYVE dependency data	DepMap, Broad 2023<br>DepMap 23Q2 Public	https://depmap.org/portal/gene/PIKFYVE?tab=overview
OPRL1 dependency data	DepMap, Broad 2023<br>DepMap 23Q2 Public	https://depmap.org/portal/gene/OPRL1?tab=overview
OPRL1 expression data	DepMap, Broad 2023<br>DepMap 23Q4 Public	https://depmap.org/portal/gene/OPRL1?tab=characterization&characterization=expression
Experimental Models: Cell Lines		
K562	J.D. Griffin	ACC-10,<br>RRID_CVCL_0004
NOMO1	J.D. Griffin	ACC-542,<br>RRID_CVCL_1609

P12-Ichikawa	A.T. Look	ACC-34, RRID_CVCL_1630
SEM	CCLE	ACC-546, RRID_CVCL_0095
Oligonucleotides		
Primers for Illumina sequencing	Whitehead Institute FGP	table S5
Primers for sgRNA quantification	Whitehead Institute FGP	table S5
Primers for construction of expression plasmids	This paper	table S5
Individual sgRNA target sequences	This paper	table S5
DHFR gBlock	This paper	table S5
TK2 gBlock	This paper	table S5
Recombinant DNA		
pLJC2-Rap2A-3xFLAG	(*13*)	Addgene 87974
pLJC2-TK2-3xFLAG	This paper	Addgene 217427
pLJC2-DHFR-3xFLAG	This paper	Addgene 217428
pcDNA3_N-DYK_MTHFD1-NES	(*104)*	Addgene 133022
pLenti-PGK-Venus-AkaLuc (neo)	(*105*)	Addgene 124701
pLJC2-MTHFD1-3xFLAG	This paper	Addgene 217429
pLentiCRISPR-v1	Addgene	Addgene 49535
Genome-wide human sgRNA library	Whitehead Institute FGP	N/A
pLentiCRISPR-v1-sgAAVS1	(*106*)	Addgene 70661
pLentiCRISPR-v1-sgTYMS	This paper	Addgene 217430
pLentiCRISPR-v1-sgTK2_4	This paper	Addgene 217431
pLentiCRISPR-v1-sgTK2_5	This paper	Addgene 217432
pLentiCRISPR-v1-sgABCC4_1	This paper	Addgene 217433

pLentiCRISPR-v1-sgABCC4_3	This paper	Addgene 217434
pLentiCRISPR-v1-sgSHMT2	This paper	Addgene 217435
pLentiCRISPR-v1-sgMTHFD1	This paper	Addgene 217436
Software and Algorithms		
XCalibur version 4.1	Thermo Fisher	https://www.thermofisher.com/us/en/home.html
R studio version 2022.12.0	The R Project	https://www.r-project.org/
Prism version 9	GraphPad	https://www.graphpad.com/
Other		
SnakeSkin dialysis tubing, 3.5K MWCO, 35 mm	Thermo Fisher	Cat #: PI88244
Z2 Coulter Counter	Beckman	Cat #: 6605700
Multisizer 4e Coulter Counter	Beckman	Cat #: B23005

Supplementary Methods

Mouse studies

Pilot experiment for plasma brivudine detection

Mice were divided into three experimental groups: vehicle (*n* = 2), low-dose BVDU (*n* = 3), and high-dose BVDU (*n* = 3). Six hours after intraperitoneal (IP) injection of 200 µL vehicle (8.4% DMSO), low-dose BVDU (10 mg/kg; 1.7% DMSO), or high-dose BVDU (50 mg/kg; 8.4% DMSO), blood was collected into heparinized tubes from the facial vein, and in turn, plasma fractions were collected following centrifugation at 1,500 *g* for 5 min. at 4°C, snap-frozen with liquid nitrogen, and stored at -80°C.

Xenograft treatments

Mice were sublethally irradiated (2 Gy) on day -8 and then injected via tail vein with 2×10^6 Akaluciferase-expressing K562 cells (in 200 µL PBS) on day -7. Tumor burden was measured on day -1 via bioluminescent imaging (BLI) after IP injection of 100 µL TokeOni (25 mM in PBS) per mouse using the IVIS Spectrum in vivo imaging system and Living Image Software (Perkin Elmer). Mice were then divided into two experimental groups based on equivalent average total flux: treatment with vehicle (*n* = 5) or BVDU (*n* = 6). Starting on day 0, mice received twelve daily treatments over two weeks via IP injection of 200 µL vehicle (8.4% DMSO) or BVDU (50 mg/kg; 8.4% DMSO). Treatment was not administered on day 7 (concurrent BLI) or day 11 (unexpected issue with facility access). Tumor burden was assessed on days 7 and 13 using BLI. Eight hours after treatment was administered on day 6, blood was collected into heparinized tubes from the facial vein, and in turn, plasma fractions were collected following centrifugation at 1,500 *g* for 5 min. at 4°C, snap-frozen with liquid nitrogen, and stored at -80°C. Plasma samples were similarly collected and stored on day 14, approximately twenty-four hours after the final treatments were administered. Injections and imaging were done by P.V.V. and R.M.R. and were not done blindly. Mice were monitored daily for health status and euthanized if symptoms of low health status appeared: hunched posture, impaired mobility, rough coat, paralysis, or significant weight loss.

Quantification and Statistical Analysis

High-throughput chemical screens

Normalized viability data from the NCATS screening platform were fit to a 4-parameter log-logistic model using the *drc* package in R to generate dose-response curves (*102*). For two compounds duplicated in the MiPE 4.1 library (NCGC00160391 and NCGC00179501), viability values at each concentration were averaged prior to similarly fitting the data, resulting in 17,784 cell line-medium-compound combinations. A subset of 654 curves (3.6% of the total dataset) failed to converge when fit to the logistic model. These were largely associated with compounds that had minimal effects on viability but also showed high data variability that prevented model convergence. To obtain metrics for further downstream analysis, this subset was instead fit using linear regression.

Areas under the dose-response curve (AUC) were calculated using the trapezoidal rule at the eleven $\log_{10}$-transformed dosing concentrations. Most fitted curves showed maximum values greater than the untreated controls used for normalization. Therefore, to reduce potential false

positives in calculating differential AUC values between screens, curves with a maximum viability greater than 100% – and the corresponding curve metrics (AUC, minimum viability, and residual standard error) – were scaled by the maximum curve value. Moreover, a small subset of curves also exhibited a sharp decrease in viability over the two lowest dosing concentrations, in turn likely generating artifacts with this scaling method. Therefore, a subset of 73 curves that exhibited a maximum value greater than 100% and a 30% decrease in viability over the two lowest doses were refit to a linear model as well.

Residual standard error (RSE) distributions varied by cell line, with the SEM line exhibiting the largest median RSE. Thus, to again minimize potential false positives, curves with RSE values that were above cell line-dependent 98th percentiles following viability scaling were removed from further downstream analysis. Next, to minimize potential false positives due to variance in initial viability measurements rather than to compound activity, cell line-specific curves with greater than 15% differences in maximum viability between two or more conditions for a given compound were also removed from further downstream analysis. At this point, filtered compounds not represented over all three conditions across cell lines were removed as well. Our collective curve fitting and filtering strategies established cell line-specific sets of compounds remaining for all three media: NOMO1 (1,871), SEM (1,761), and P12-Ichikawa (1,894). From these cell line-specific sets, 1,638 total compounds were common across cell line-medium combinations. Lastly, a set of 500 pan-inactive compounds were defined on the basis of exhibiting minimum scaled values greater than respective median scaled values in each of the nine screen datasets. Response scores for the 1,138 filtered compounds were defined as corresponding AUCs. For each compound, differential response scores in each cell line were calculated between each pairwise set of conditions and then standardized (Z-score) relative to the entire set of 1,138 active compounds to assess differential sensitivity. For each compound, the differential sensitivity scores were then averaged across cell lines for each pairwise set of conditions.

Sets of RPMI-sensitive and HPLM-sensitive hits from the averaged HPLM^{+dS} – RPMI^{+dS} profile were defined by setting a Z-score cutoff of 1. Sets of dS-sensitive and S-sensitive based on shared conditional phenotypes in the averaged HPLM^{+dS} – RPMI^{+S} and RPMI^{+dS} – RPMI^{+S} profiles were defined by setting a Z-score cutoff of 0.9. After removing dS- and S-sensitive hits from consideration, sets of RPMI-sensitive and HPLM-sensitive hits based on shared conditional phenotypes in the averaged HPLM^{+dS} – RPMI^{+dS} and HPLM^{+dS} – RPMI^{+S} profiles were defined by setting a Z-score cutoff of 0.9.

<u>Genome-wide CRISPR screens</u>

CRISPR screen analysis was performed as previously described (*27*). Sequencing reads were aligned to the sgRNA library to generate read counts and only exact matches were allowed. sgRNAs with less than 50 counts in the initial population were removed from further downstream analysis. Genes targeted by less than four distinct sgRNAs following this filtering process were also removed. The relative abundances of all remaining sgRNAs were determined by adding a pseudocount of one and then normalizing to the total reads in the sample. Depletion scores were calculated as the $\log_2$ fold-change in sgRNA abundance between the initial population and each final population. Gene scores were defined as the average $\log_2$ fold-change in depletion scores of all sgRNAs targeting the gene.

Screens performed in different conditions may introduce discrepancies in aggregate gene selection that affect the dynamic range of gene scores (*103*). Therefore, to reduce potential bias in

calculating differential scores based on assuming that such distributions are equivalent between screens, we scaled all gene scores instead based on the assumption that the sets of nontargeting (NT) sgRNAs and core essential genes (CEGs) would exhibit the same selection across different screens. Gene scores were scaled such that the medians of post-filtering NT sgRNAs (449) and reference CEGs (682 genes) (*65*) included in the library were defined as 0 and -1, respectively (*27*). For each gene, a differential score was calculated between the two screening conditions and then standardized (Z-score) relative to the entire set of differential scores.

Probability of dependency for genome-wide CRISPR screens

For each genome-wide screen, probabilities of dependency (PODs) were calculated for all library targets (*27*). Briefly, the gene score dataset from each screen was treated as a mixture model comprised of two normal distributions – distinct sets of non-essential and essential genes, with the latter having the lower mean. Densities were generated using a standard E-M optimization procedure initialized with parameters (mean, standard deviation, proportional value) of (-1, 0.3, 0.1) and (-0.2, 0.15, 0.9) for the reference sets of essential and non-essential genes, respectively. These initial values were based on empirical observations of score distributions for CEGs and nonessential genes from previous screens (*27*, *65*). The POD for a given gene was then calculated as the ratio of CEG density to the sum of the two densities at the gene score of interest. Given that standard deviations of the two distributions differ, their estimated densities converge to zero at different rates in tail regions, which can cause erroneous inflation of estimated probabilities at large enough gene score values. Thus, we identified the minimum POD and its corresponding gene score in each screen, and in turn, assigned the minimum probability to all targets with a gene score greater than that value.

Receiver-operator analysis

For each CRISPR screen dataset, receiver-operator characteristic (ROC) curves were generated from relatively balanced reference sets of 682 CEG and 879 nonessential genes (*65*). Area under the ROC curve was used as the performance metric to assess how well gene scores in each dataset could discriminate for CEGs.

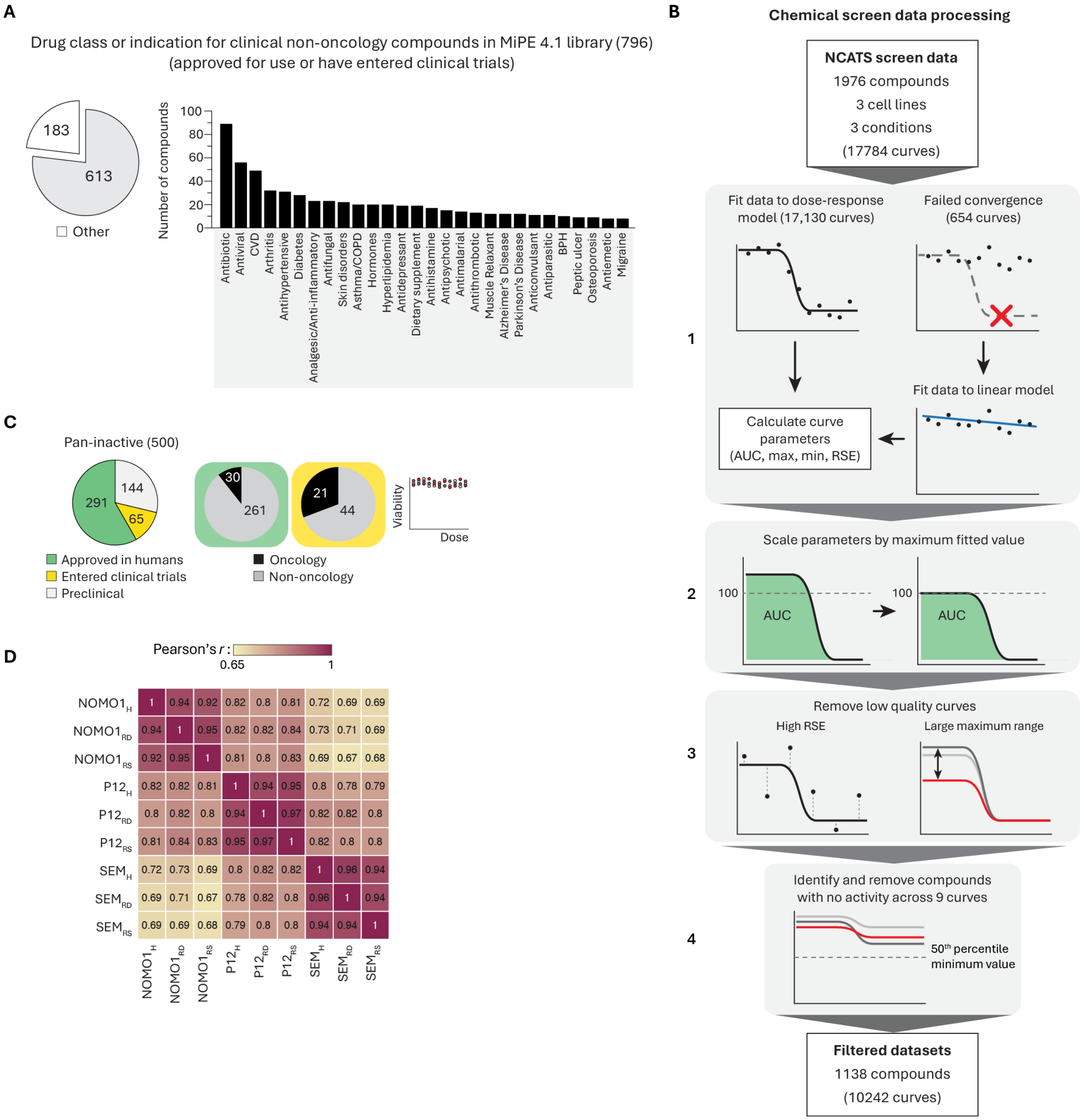

Fig. S1. Chemical screen analysis

(**A**) Manually curated drug class or indication for MiPE 4.1 library compounds either approved for use in humans or that have entered clinical trials. CVD, cardiovascular disease; COPD, chronic obstructive pulmonary disease; BPH, benign prostatic hyperplasia.

(**B**) Data processing workflow. See **Quantification and Statistical analyses** for additional detail. (1) Normalized viability data for 17,784 total dose-response curves were fit to a 4-parameter log-logistic model. 654 curves failed to converge and were fit using linear regression. (2) Curves with a maximum viability greater than 100% were scaled by the maximum fitted value. (3) Curves with residual standard error (RSE) values above cell line-dependent 98th percentiles were removed (left);

Cell line-specific curves with greater than a 15% difference in maximum viability between two or more conditions for a given compound were removed (right). (4) Compounds with minimum values greater than the respective median values in each of the datasets were removed. Ultimately, 1,138 filtered compounds were shared across all screens. AUC, area under the curve.
(**C**) Highest global development phase and indication for pan-inactive compounds.
(**D**) Response score correlations between nine chemical screens. H, $HPLM^{+dS}$; RD, $RPMI^{+dS}$; RS, $RPMI^{+S}$.

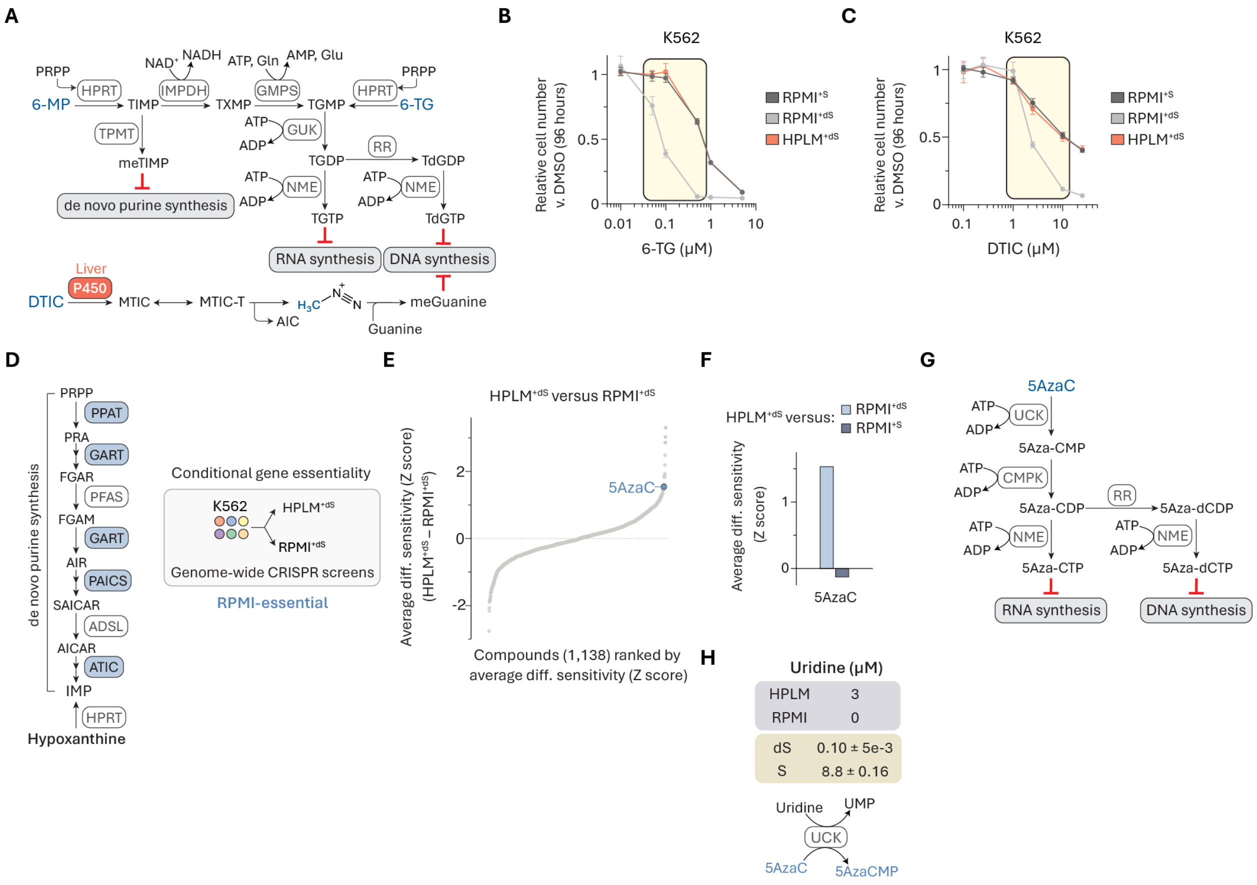

Fig. S2. Additional data related to conditional phenotypes for purine analogs are linked to hypoxanthine

(**A**) Cellular conversion of RPMI-sensitive purine analogs to effector metabolites. The canonical mechanism of dacarbazine (DTIC) activity involves an activation step catalyzed by liver-specific P450. 6-MP, 6-Mercaptopurine; 6-TG, 6-Thioguanine.

(**B–C**) Dose-responses of K562 cells to 6-TG (B) and DTIC (C) (mean ± SD, n = 3). Concentration range spanned for two dose-responses tested across the remaining three cell lines (yellow box).

(**D**) Schematic for the de novo purine synthesis pathway. Enzymes encoded by genes that were identified as RPMI-essential hits from previous genome-wide CRISPR screens in K562 cells (shaded blue) *(27)*. Hypoxanthine is a salvage pathway substrate that can be used to generate IMP.

(**E**) Compounds ranked by average HPLM^{+dS} – RPMI^{+dS} phenotypes. 5AzaC, 5-Azacytidine.

(**F**) Conditional phenotypes for 5AzaC from averaged HPLM^{+dS} – RPMI^{+dS} and HPLM^{+dS} – RPMI^{+S} profiles.

(**G**) Cellular conversion of 5AzaC to metabolites to effector metabolites.

(**H**) Defined uridine levels in HPLM and RPMI. Uridine levels in 10% FBS (dS, dialyzed; S, untreated) (mean ± SD, n = 3). Schematic of reaction catalyzed by UCK, uridine-cytidine kinase.

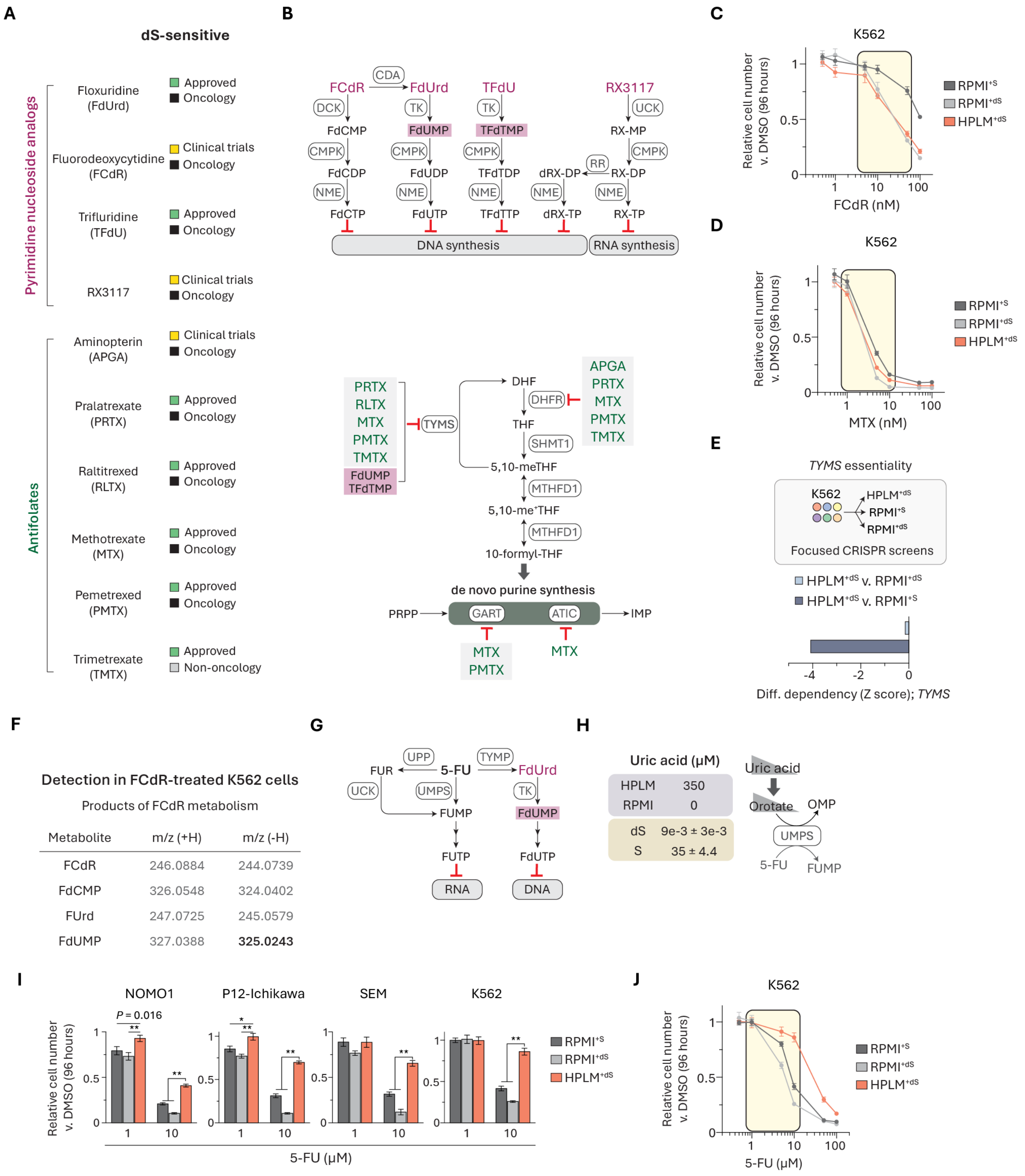

Fig. S3. Additional data related to serum-derived thymidine alters cellular sensitivity to TYMS inhibitors

(**A**) dS-sensitive pyrimidine nucleoside analogs (top) and antifolates (bottom).

(**B**) Pyrimidine nucleoside analogs are converted to effector metabolites (top). Antifolates act against targets in 1C metabolism (bottom). Fluorodeoxyuridine monophosphate (FdUMP) and trifluoromethyl deoxyuridine monophosphate (TFdTMP) can also each inhibit TYMS.

(**C–D**) Dose-responses of K562 cells to FCdR (C) and MTX (D) (mean ± SD, $n = 3$). Concentration range spanned for two dose-responses tested across the remaining three cell lines (yellow box).
(**E**) Conditional CRISPR phenotypes for *TYMS* from reported focused sgRNA library screens in K562 cells (*27*).
(**F**) Mass-to-charge ratios (m/z) for various products of cellular FCdR metabolism based on either addition (+H) or removal (-H) of a proton adduct. Only peaks corresponding to FdUMP in negative ionization mode (-H) could be detected in FCdR-treated K562 cells.
(**G**) Schematic of 5-fluorouracil (5-FU) metabolism. Floxuridine (FdUrd) was a dS-sensitive hit.
(**H**) Defined uric acid levels in HPLM and RPMI. Uric acid levels in 10% FBS (dS, dialyzed; S, untreated) (mean ± SD, $n = 3$). Uric acid is an endogenous inhibitor of UMP synthase (UMPS). Therefore, uric acid availability impacts cellular levels of orotate, which competes with 5-FU as a substrate for UMPS activity (*13)*.
(**I**) Relative growth of cells treated with 5-FU versus DMSO (mean ± SD, $n = 3$, $^{**}P < 0.005$, $^{*}P < 0.01$).
(**J**) Dose-responses of K562 cells to 5-FU (mean ± SD, $n = 3$). Concentration range spanned for two dose-responses tested across the remaining three cell lines (yellow box).

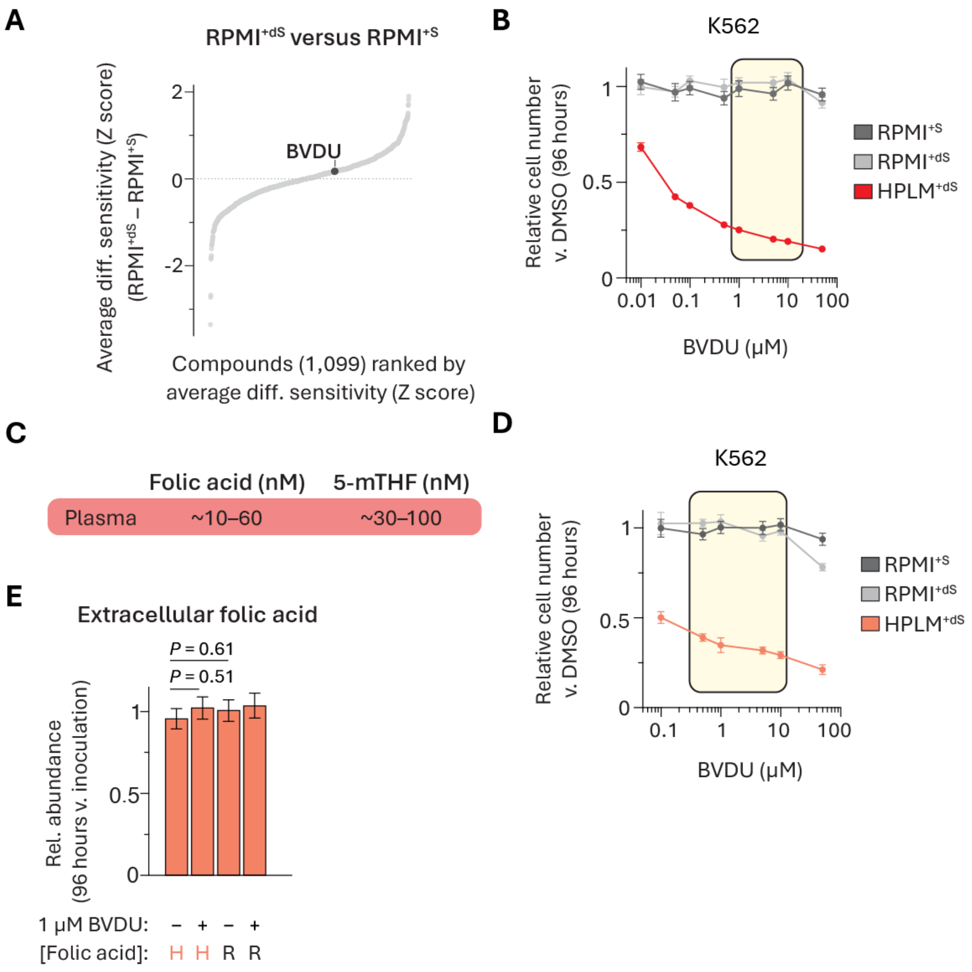

Fig. S4. Additional data related to conditional brivudine sensitivity is linked to folic acid availability

(**A**) Compounds ranked by average RPMI^{+dS} – RPMI^{+S} phenotypes. BVDU, brivudine.

(**B** and **D**) Dose-responses of K562 cells to BVDU (mean ± SD, *n* = 3). Concentration range spanned for two dose-responses tested across the remaining three cell lines (yellow box).

(**C**) Reported concentration ranges for folic acid and 5-methyl-THF (5-mTHF) in human plasma (*60*–*62*).

(**E**) Extracellular abundances of folic acid in HPLM^{+dS} following 96-hour treatment with BVDU versus those at inoculation (mean ± SEM, *n* = 3).

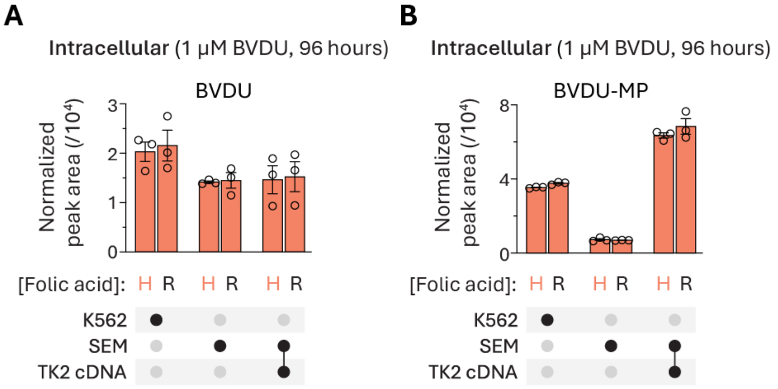

Fig. S5. Additional data related to TK2 expression is an intrinsic determinant of BVDU sensitivity

(**A** and **B**) Cellular abundances of BVDU (A) and BVDU-MP (B) following BVDU treatment in HPLM^{+dS} (mean ± SEM, n = 3). H, HPLM-defined concentration (0.45 µM). R, RPMI-defined concentration (2.27 µM).

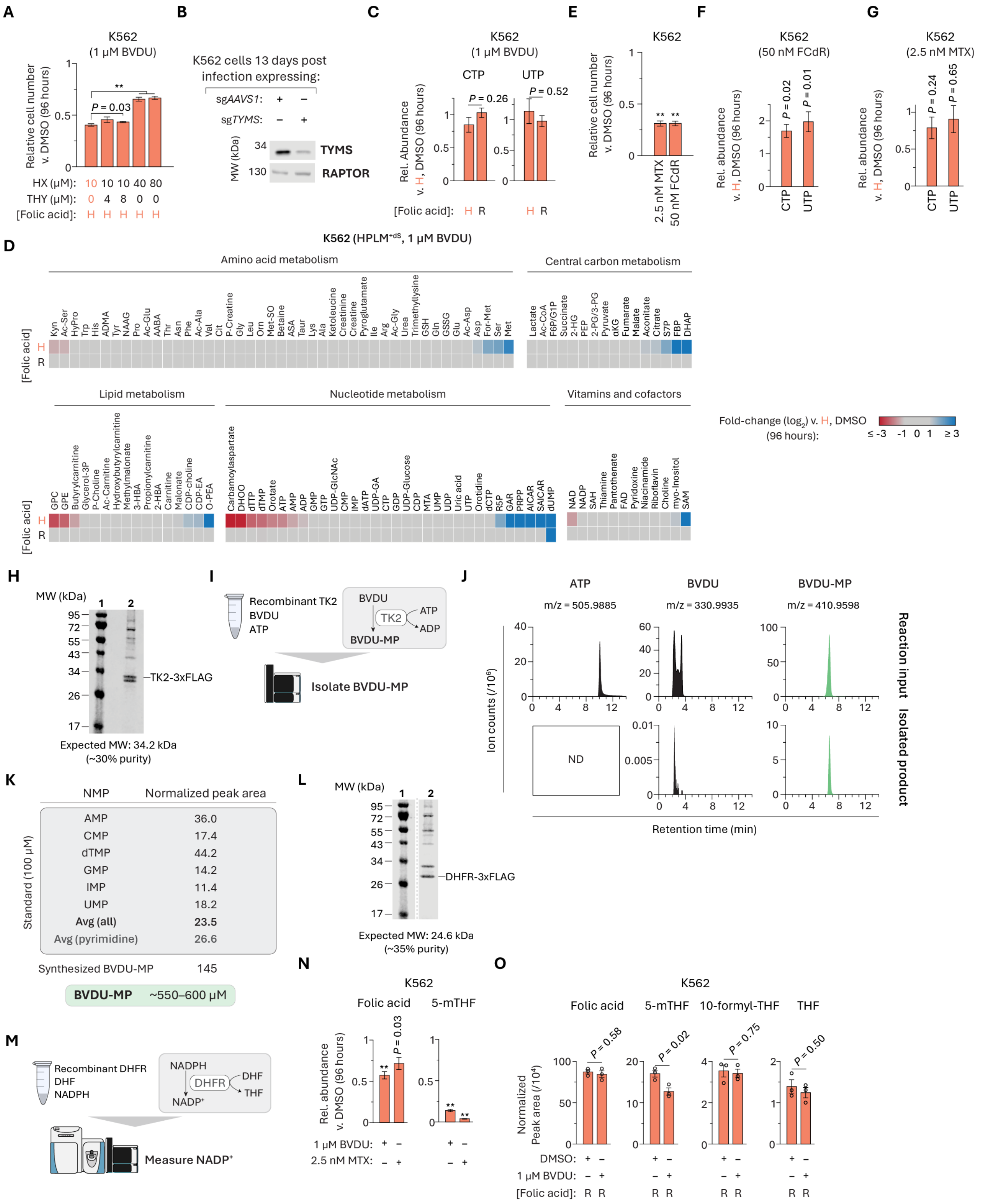

A
K562
(1 μM BVDU)
Relative cell number v. DMSO (96 hours)
P = 0.03
**
HX (μM): 10 10 10 40 80
THY (μM): 0 4 8 0 0
[Folic acid]: H H H H H
B
K562 cells 13 days post infection expressing:
sgAAVS1: + –
sgTYMS: – +
MW (kDa)
34
130
TYMS
RAPTOR
C
K562
(1 μM BVDU)
CTP
UTP
P = 0.26
P = 0.52
Rel. Abundance v. H, DMSO (96 hours)
[Folic acid]: H R H R
E
K562
Relative cell number v. DMSO (96 hours)
2.5 nM MTX
50 nM FCdR
F
K562
(50 nM FCdR)
Rel. abundance v. H, DMSO (96 hours)
P = 0.02
P = 0.01
CTP
UTP
G
K562
(2.5 nM MTX)
P = 0.24
P = 0.65
CTP
UTP
K562 (HPLM+dS, 1 μM BVDU)
D
Amino acid metabolism
Central carbon metabolism
[Folic acid]
Kyn Ac-Ser HyPro Trp His ADMA Tyr NAAG Pro Ac-Glu AABA Thr Asn Phe Ac-Ala Val Cit P-Creatine Gly Leu Orn Met-SO Betaine ASA Taur Lys Ala Ketoleucine Creatinine Creatine Pyroglutamate Ile Arg Ac-Gly Urea Trimethyllysine GSH Gln GSSG Glu Ac-Asp Asp For-Met Ser Met
Lactate Ac-CoA F6P/G1P Succinate 2-HG PEP 2-PG/3-PG Pyruvate αKG Fumarate Malate Aconitate Citrate S7P FBP DHAP
Lipid metabolism
Nucleotide metabolism
Vitamins and cofactors
GPC GPE Butyrylcarnitine Glycerol-3P P-Choline Ac-Carnitine Hydroxybutyrylcarnitine Methylmalonate 3-HBA Propionylcarnitine 2-HBA Carnitine Malonate CDP-choline CDP-EA O-PEA
Carbamoylaspartate DHOO dTTP dTMP Orotate ATP AMP ADP GMP GTP UDP-GlcNAc CMP IMP dATP UDP-GA CTP GDP UDP-Glucose CDP MTA UMP UDP Uric acid UTP Orotidine dCTP R5P GAR PRPP AICAR SAICAR dUMP
NAD NADP SAH Thiamine Pantothenate FAD Pyridoxine Niacinamide Riboflavin Choline myo-Inositol SAM
Fold-change (log2) v. H, DMSO (96 hours):
≤ -3 -1 0 1 ≥ 3
H
MW (kDa)
1 2
95 72 55 43 34 26 17
TK2-3xFLAG
Expected MW: 34.2 kDa (~30% purity)
I
Recombinant TK2
BVDU
ATP
TK2
ADP
BVDU-MP
Isolate BVDU-MP
J
ATP
m/z = 505.9885
BVDU
m/z = 330.9935
BVDU-MP
m/z = 410.9598
Ion counts (/10^6)
ND
Retention time (min)
Reaction input
Isolated product
K
NMP
Normalized peak area
Standard (100 μM)
AMP 36.0
CMP 17.4
dTMP 44.2
GMP 14.2
IMP 11.4
UMP 18.2
Avg (all) 23.5
Avg (pyrimidine) 26.6
Synthesized BVDU-MP 145
BVDU-MP ~550–600 μM
L
MW (kDa)
1 2
95 72 55 43 34 26 17
DHFR-3xFLAG
Expected MW: 24.6 kDa (~35% purity)
M
Recombinant DHFR
DHF
NADPH
DHFR
THF
NADP+
Measure NADP+
N
K562
Folic acid
5-mTHF
P = 0.03
Rel. abundance v. DMSO (96 hours)
1 μM BVDU: + – + –
2.5 nM MTX: – + – +
O
K562
Folic acid
5-mTHF
10-formyl-THF
THF
P = 0.58
P = 0.02
P = 0.75
P = 0.50
Normalized Peak area (/10^4)
DMSO: + – + – + – + –
1 μM BVDU: – + – + – + – +
[Folic acid]: R R R R R R R R

Fig. S6. Additional data related to BVDU-MP interferes with folate-dependent nucleotide synthesis and DHFR is not the molecular target of BVDU-MP

(**A**) Relative growth of cells treated with BVDU versus DMSO (mean ± SD, n = 3, $^{**}P < 0.005$). H, HPLM-defined concentration (0.45 μM).

(**B**) Immunoblot for expression of TYMS. M.W. standards are annotated. RAPTOR served as the loading control. TYMS band intensities differ by ~5-fold between the two samples.

(**C**) Relative abundances of CTP and UTP in BVDU-treated versus control cells in $HPLM^{+dS}$ (mean ± SEM, n = 3, $^{**}P < 0.005$).

(**D**) Heatmap of cellular metabolite abundances in cells treated with BVDU in $HPLM^{+dS}$ containing HPLM- (top) or RPMI-defined (bottom) folic acid versus control cells in $HPLM^{+dS}$. Metabolite clusters are sorted by $\log_2$-transformed fold change of the top row (n = 3). Metabolite abbreviations can be found in table S3.

(**E**) Relative growth of cells treated with FCdR or MTX versus DMSO (mean ± SD, n = 3, $^{**}P <$ 0.005).

(**F–G**) Relative abundances of CTP and UTP in FCdR- (F) and MTX-treated (G) versus control cells in $HPLM^{+dS}$ (mean ± SEM, n = 3, $^{**}P < 0.005$).

(**H**) Pseudocolor Coomassie-stained gel imaged using a LICOR Odyssey FC. 1: M.W. standards, 2: TK2-3xFLAG.

(**I**) Schematic for a method to isolate BVDU-MP from reactions catalyzed by human TK2.

(**J**) Extracted ion chromatograms at mass-to-charge (m/z) ratios, in negative ionization mode, for ATP, BVDU, and BVDU monophosphate (BVDU-MP) at indicated retention times for samples extracted from reactions containing purified recombinant TK2 with ATP and BVDU (top) and the isolated BVDU-MP (bottom). See **Methods**.

(**K**) Normalized peak areas across a panel of NMP chemical standards and in vitro synthesized BVDU-MP. A concentration for the stock BVDU-MP was estimated based on the average of these standard areas – with little effect on this average if considering only the pyrimidine NMPs.

(**L**) Pseudocolor Coomassie-stained gel imaged using a LICOR Odyssey FC. 1: M.W. standards, 2: DHFR-3xFLAG.

(**M**) Schematic for a method to evaluate DHFR activity based on measuring $NADP^+$ production from reactions containing recombinant DHFR.

(**N–O**) Relative abundances of indicated folate species in BVDU- and MTX-treated versus control cells in $HPLM^{+dS}$ containing RPMI-defined folic acid (mean ± SEM, n = 3).

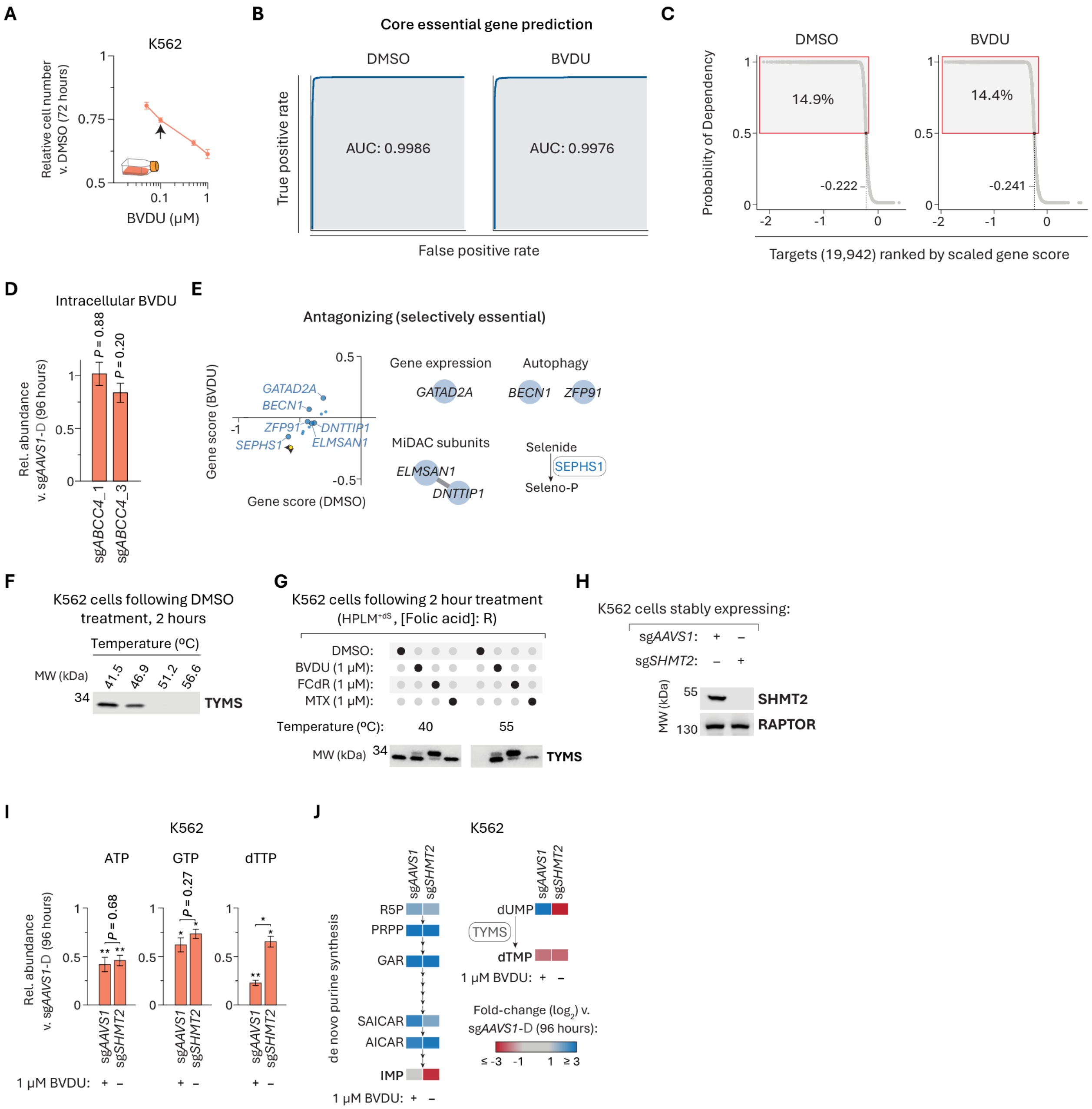

Fig. S7. Additional data related to CRISPR screens uncover genetic contributions to BVDU sensitivity

(**A**) Relative growth of K562 cells treated with BVDU versus DMSO in T-25 flasks (mean ± SD, n = 3). Arrow indicates the dose that elicited a ~25% growth defect.

(**B**) Receiver operator characteristic (ROC) curves for the prediction of core essential genes using datasets from CRISPR screens in DMSO- and BVDU-treated K562 cells.

(**C**) Plots of sgRNA library targets ranked by probability of dependency from genome-wide K562 screens in HPLM^{+dS} with DMSO vehicle (left) or BVDU treatment (right). Red box indicates probability > 0.5. Dashed lines mark gene scores at the cutoffs for gene essentiality in each screen.

(**D**) Relative abundance of BVDU in *ABCC4*-knockout versus control cells following treatment with BVDU in HPLM^{+dS} (mean ± SEM, n = 3).
(**E**) Selectively essential BVDU-antagonizing hits.
(**F**) Immunoblot for expression of TYMS in cells treated with vehicle in HPLM^{+dS} across indicated temperatures for cellular thermal shift assay (CETSA). M.W. standard is annotated.
(**G**) Immunoblot for expression of TYMS in cells after treatment with DMSO, BVDU, FCdR, or MTX in HPLM^{+dS} with RPMI-defined folic acid at indicated CETSA temperatures.
(**H**) Immunoblot for expression of SHMT2. M.W. standards are annotated. RAPTOR served as the loading control.
(**I**) Relative abundances of ATP, GTP, and dTTP in BVDU-treated control and *SHMT2*-knockout cells versus control cells in HPLM^{+dS} (mean ± SEM, n = 3, $^{**}P < 0.005$, $^{*}P < 0.01$).
(**J**) Heatmap of cellular abundances for indicated metabolites in BVDU-treated control and *SHMT2*-knockout cells versus control cells in HPLM^{+dS} (n = 3).

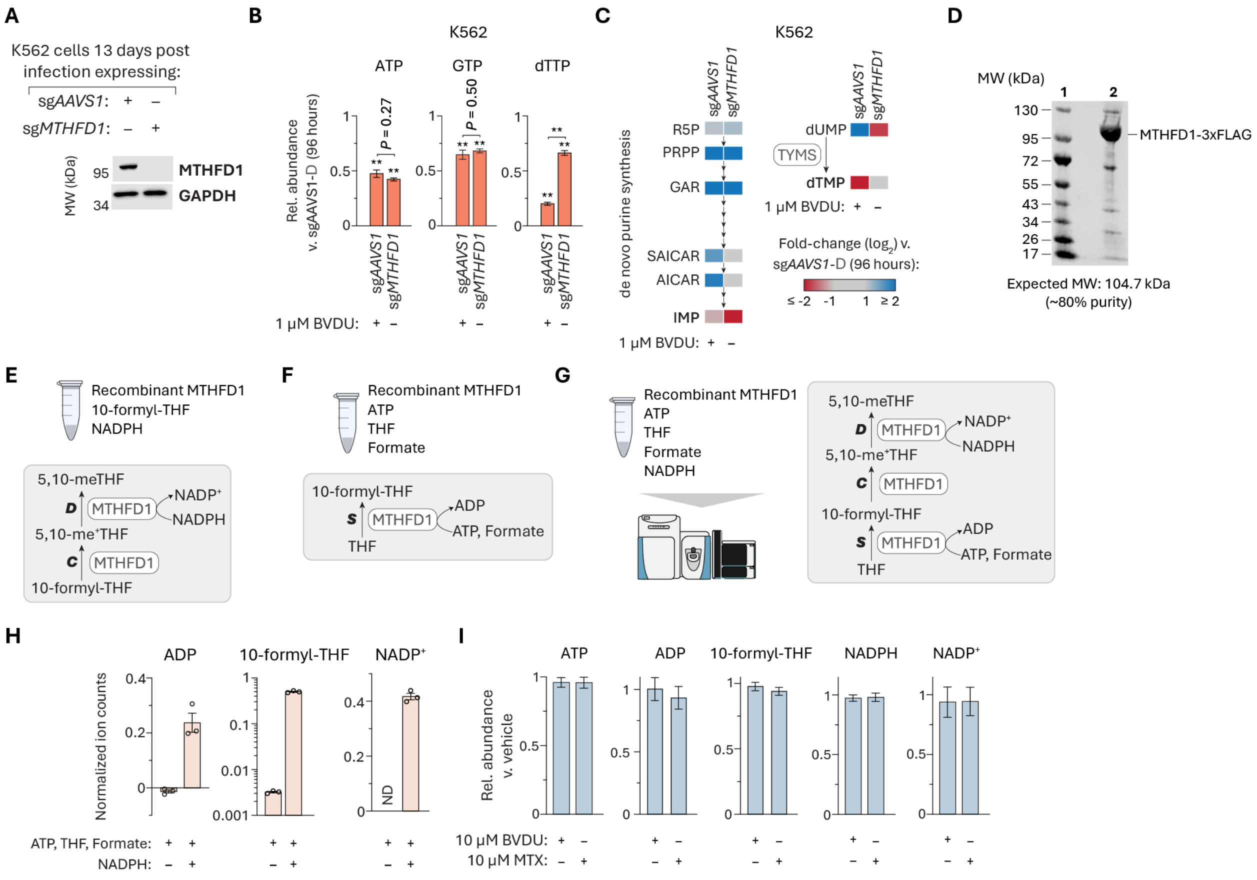

Fig. S8. Additional data related to BVDU-MP affects the 10-formyl-THF synthetase activity of MTHFD1

(**A**) Immunoblot for expression of MTHFD1. M.W. standards are annotated. GAPDH served as the loading control.

(**B**) Relative abundances of ATP, GTP, and dTTP in BVDU-treated control and *MTHFD1*-knockout cells versus control cells in HPLM^{+dS} (mean ± SD, n = 3, **P < 0.005).

(**C**) Heatmap of cellular abundances for indicated metabolites in BVDU-treated control and *MTHFD1*-knockout cells versus control cells in HPLM^{+dS} (n = 3).

(**D**) Pseudocolor Coomassie-stained gel imaged using a LICOR Odyssey FC. 1: M.W. standards, 2: MTHFD1-3xFLAG.

(**E**) Schematic for a method to evaluate MTHFD1(CD) domain activity.

(**F**) Schematic for a method to evaluate MTHFD1(S) activity.

(**G**) Schematic for a method to evaluate reaction components across both MTHFD1 domains.

(**H**) Normalized ion counts for ADP, 10-formyl-THF, and NADP$^+$ from reactions of recombinant MTHFD1 with ATP, THF, and formate that either lack (top) or further contain (bottom) NADPH (mean ± SD, n = 3). Correction for background ADP resulted in slightly negative ion counts from reactions in the absence of NADPH, reflective of noise.

(**I**) Relative levels of indicated metabolites measured from multi-domain MTHFD1 activity assays following addition of BVDU or MTX (mean ± SEM, n = 3).

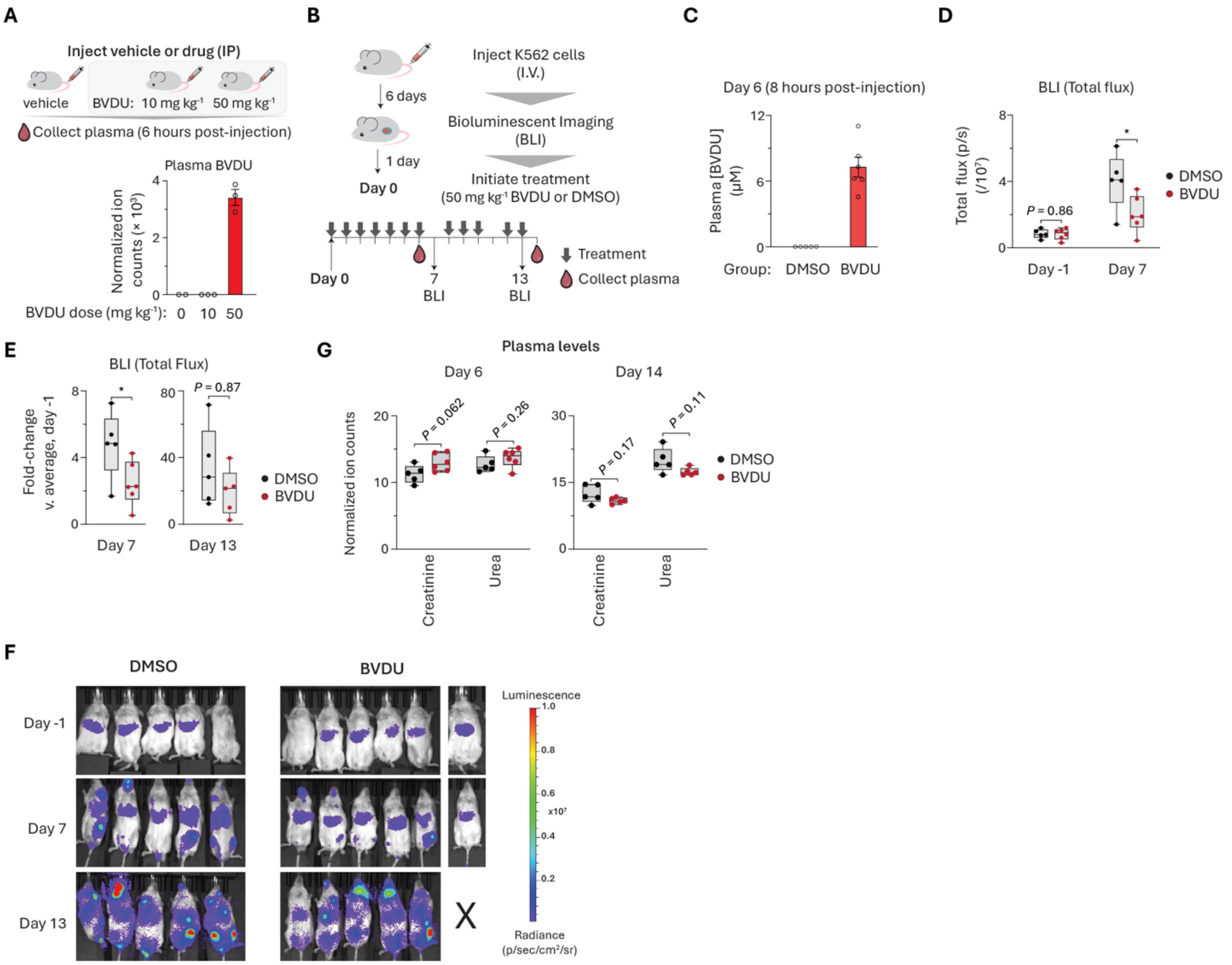

Fig. S9. BVDU treatment reduces in vivo tumor burden

(**A**) Schematic for assessing detection of BVDU in plasma samples collected from NSG mice 6 hours after intraperitoneal (IP) injection of vehicle or BVDU at one of two doses (top). Normalized ion counts for plasma BVDU (mean ± SEM, vehicle n = 2; 10 mg kg^{-1} BVDU n = 3; 50 mg kg^{-1} BVDU n = 3) (bottom).

(**B**) Schematic for assessing how BVDU affects the growth of K562 xenografts in NSG mice. K562 cells expressing Akaluciferase were injected intravenously via tail vein. After 6 days, tumor burden was measured with bioluminescence imaging (BLI). Mice were assigned to two groups: treatment with vehicle (n = 5) or BVDU (n = 6). Starting on day 0, twelve total daily doses of vehicle (8.4% DMSO) or 50 mg/kg BVDU (in 8.4% DMSO) were administered by IP injection. Tumor burden was measured on days 7 and 13 using BLI. Plasma was collected on day 6 (roughly 8 hours after treatment was administered) and on day 14.

(**C**) Plasma BVDU levels measured from vehicle- and BVDU-treated mice on day 6 (mean ± SEM, vehicle n = 5; BVDU n = 6).

(**D**) Quantification of BLI signals (total flux) at the indicated time points for each treatment group. In the box plots, the center line indicates the median, box limits mark the first and third quartiles, and the whiskers represent the minimum and maximum of all data points. *$P < 0.05$.

(**E**) Changes in BLI signal (total flux) at the indicated time points for each group versus at day -1. Box plot parameters as in panel L. *$P < 0.05$.

(**F**) BLI images depicting tumor burden of K562-engrafted mice treated with vehicle (left) or BVDU (right) at indicated time points. Data are represented colorimetrically (photons/s/cm^2/sr) with the scale bar depicted. X, death within the group prior to time point.

(**G**) Normalized ion counts for creatinine and urea in plasma collected from each treatment group at the indicated time points. Box plot parameters as in panel D.

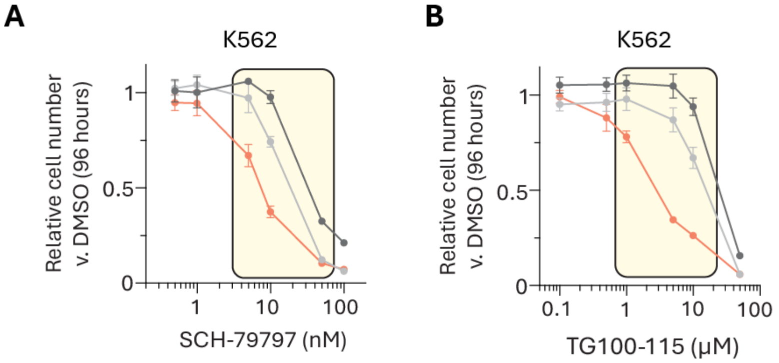

Fig. S10. Additional data related to conditional phenotypes for additional compounds are linked to folic acid

(**A** and **B**) Dose-responses of K562 cells to SCH-79797 (A) and TG100-115 (B) (mean ± SD, n = 3). Concentration range spanned for two dose-responses tested across the remaining three cell lines (yellow box).

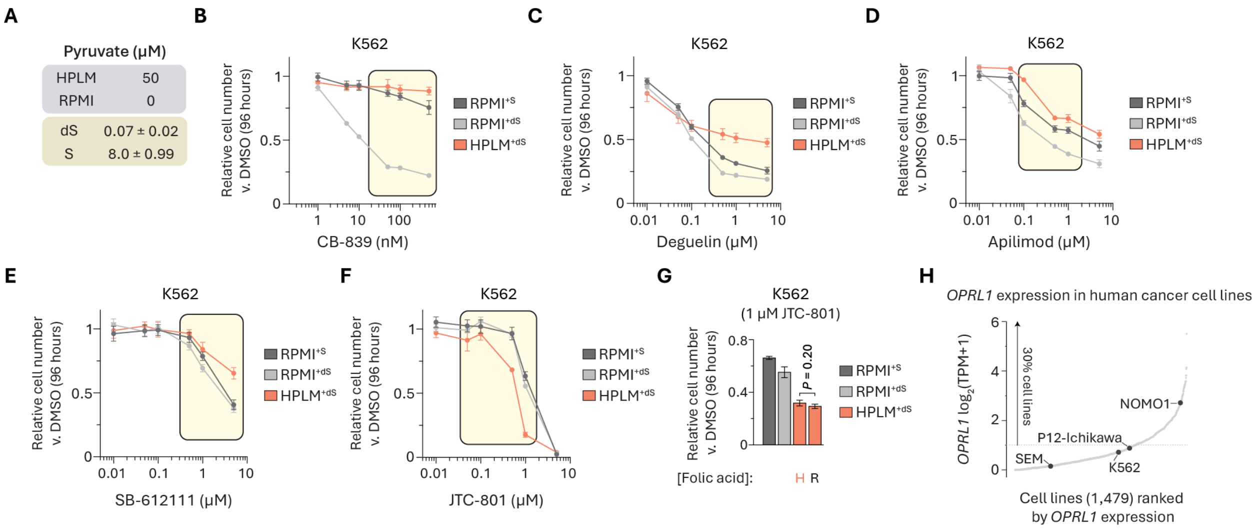

Fig. S11. Additional data related to gene essentiality data suggest that other conditional phenotypes are linked to non-canonical mechanisms

(**A**) Defined pyruvate levels in HPLM and RPMI. Pyruvate levels in 10% FBS (dS, dialyzed; S, untreated) (mean ± SD, n = 3).

(**B–F**) Dose-responses of K562 cells to CB-839 (B), deguelin (C), apilimod (D), SB-612111 (E), or JTC-801 (F) (mean ± SD, n = 3). Concentration range spanned for two dose-responses tested across the remaining three cell lines (yellow box).

(**G**) Relative growth of cells treated with JTC-801 versus DMSO (mean ± SD, n = 3, $^{**}P < 0.005$). H, HPLM-defined concentration (0.45 μM). R, RPMI-defined concentration (2.27 μM).

(**H**) Human cancer cell lines ranked by *OPRL1* expression from reported RNA-seq data (*95*). Labeled points indicate cell lines in this study. TPM, transcripts per million.

A

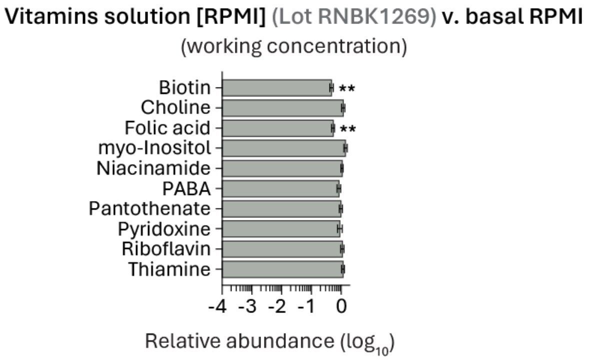

B

Biotin (μM)

Defined	0.82
Basal	0.80 ± 0.09
Solution	0.39 ± 0.02

Folic acid (μM)

Defined	2.27
Basal	2.40 ± 0.25
Solution	1.29 ± 0.01

Fig. S12. Comparative profiling analysis for a distinct lot of commercial vitamins solution
(**A**) Relative working concentrations in commercial solution (Sigma R7256, Lot RNBK1269) versus basal RPMI (Lot 2458379) (mean ± SD, n = 3, **P < 0.005).
(**B**) Defined and working concentrations of biotin and folic acid (mean ± SD, n = 3). RPMI also contains 3.69 nM vitamin B12, which could not be detected by the profiling method.

Supplementary Table Captions

table S1. Datasets related to chemical screens
table S2. Synthetic media construction
table S3. Datasets related to metabolite profiling
table S4. Datasets related to CRISPR screens
table S5. Oligonucleotides used in this study

REFERENCES AND NOTES

1. D. C. Swinney, J. Anthony, How were new medicines discovered? *Nat. Rev. Drug Discov.* **10**, 507–519 (2011).

2. J. G. Moffat, F. Vincent, J. A. Lee, J. Eder, M. Prunotto, Opportunities and challenges in phenotypic drug discovery: An industry perspective. *Nat. Rev. Drug Discov.* **16**, 531–543 (2017).

3. C. Yu, A. M. Mannan, G. M. Yvone, K. N. Ross, Y.-L. Zhang, M. A. Marton, B. R. Taylor, A. Crenshaw, J. Z. Gould, P. Tamayo, B. A. Weir, A. Tsherniak, B. Wong, L. A. Garraway, A. F. Shamji, M. A. Palmer, M. A. Foley, W. Winckler, S. L. Schreiber, A. L. Kung, T. R. Golub, High-throughput identification of genotype-specific cancer vulnerabilities in mixtures of barcoded tumor cell lines. *Nat. Biotechnol.* **34**, 419–423 (2016).

4. A. Loewa, J. J. Feng, S. Hedtrich, Human disease models in drug development. *Nat. Rev. Bioeng.* 1–15 (2023).

5. P. Horvath, N. Aulner, M. Bickle, A. M. Davies, E. D. Nery, D. Ebner, M. C. Montoya, P. Östling, V. Pietiäinen, L. S. Price, S. L. Shorte, G. Turcatti, C. von Schantz, N. O. Carragher, Screening out irrelevant cell-based models of disease. *Nat. Rev. Drug Discov.* **15**, 751–769 (2016).

6. K. V. Kitaeva, C. S. Rutland, A. A. Rizvanov, V. V. Solovyeva, Cell Culture Based in vitro Test Systems for Anticancer Drug Screening. *Front. Bioeng. Biotechnol.* **8**, 322 (2020).

7. J. R. Cantor, The rise of physiologic media. *Trends Cell Biol.* **29**, 854–861 (2019).

8. T. Ackermann, S. Tardito, Cell culture medium formulation and its implications in cancer metabolism. *Trends Cancer* **5**, 329–332 (2019).

9. T. Ast, V. K. Mootha, Oxygen and mammalian cell culture: Are we repeating the experiment of Dr. Ox? *Nat. Metab.* **1**, 858–860 (2019).

10. S. Breslin, L. O'Driscoll, Three-dimensional cell culture: The missing link in drug discovery. *Drug Discov. Today* **18**, 240–249 (2013).

11. B. A. Hassell, G. Goyal, E. Lee, A. Sontheimer-Phelps, O. Levy, C. S. Chen, D. E. Ingber, Human organ chip models recapitulate orthotopic lung cancer growth, therapeutic responses, and tumor dormancy in vitro. *Cell Rep.* **21**, 508–516 (2017).

12. K. Ronaldson-Bouchard, G. Vunjak-Novakovic, Organs-on-a-chip: A fast track for engineered human tissues in drug development. *Cell Stem Cell* **22**, 310–324 (2018).

13. J. R. Cantor, M. Abu-Remaileh, N. Kanarek, E. Freinkman, X. Gao, A. Louissaint, C. A. Lewis, D. M. Sabatini, Physiologic medium rewires cellular metabolism and reveals uric acid as an endogenous inhibitor of UMP synthase. *Cell* **169**, 258–272.e17 (2017).

14. M. J. Garnett, E. J. Edelman, S. J. Heidorn, C. D. Greenman, A. Dastur, K. W. Lau, P. Greninger, I. R. Thompson, X. Luo, J. Soares, Q. Liu, F. Iorio, D. Surdez, L. Chen, R. J. Milano, G. R. Bignell, A. T. Tam, H. Davies, J. A. Stevenson, S. Barthorpe, S. R. Lutz, F. Kogera, K. Lawrence, A. McLaren-Douglas, X. Mitropoulos, T. Mironenko, H. Thi, L. Richardson, W. Zhou, F. Jewitt, T. Zhang, P. O'Brien, J. L. Boisvert, S. Price, W. Hur, W. Yang, X. Deng, A. Butler, H. G. Choi, J. W. Chang, J. Baselga, I. Stamenkovic, J. A. Engelman, S. V. Sharma, O. Delattre, J. Saez-Rodriguez, N. S. Gray, J. Settleman, P. A. Futreal, D. A. Haber, M. R. Stratton, S. Ramaswamy, U. McDermott, C. H. Benes, Systematic identification of genomic markers of drug sensitivity in cancer cells. *Nature* **483**, 570–575 (2012).

15. F. Iorio, T. A. Knijnenburg, D. J. Vis, G. R. Bignell, M. P. Menden, M. Schubert, N. Aben, E. Gonçalves, S. Barthorpe, H. Lightfoot, T. Cokelaer, P. Greninger, E. van Dyk, H. Chang, H. de Silva, H. Heyn, X. Deng, R. K. Egan, Q. Liu, T. Mironenko, X. Mitropoulos, L. Richardson, J. Wang, T. Zhang, S. Moran, S. Sayols, M. Soleimani, D. Tamborero, N. Lopez-Bigas, P. Ross-Macdonald, M. Esteller, N. S. Gray, D. A. Haber, M. R. Stratton, C. H. Benes, L. F. A. Wessels, J. Saez-Rodriguez, U. McDermott, M. J. Garnett, A landscape of pharmacogenomic interactions in cancer. *Cell* **166**, 740–754 (2016).

16. A. Basu, N. E. Bodycombe, J. H. Cheah, E. V. Price, K. Liu, G. I. Schaefer, R. Y. Ebright, M. L. Stewart, D. Ito, S. Wang, A. L. Bracha, T. Liefeld, M. Wawer, J. C. Gilbert, A. J. Wilson, N. Stransky, G. V. Kryukov, V. Dancik, J. Barretina, L. A. Garraway, C. S.-Y. Hon, B. Munoz, J. A. Bittker, B. R. Stockwell, D. Khabele, A. M. Stern, P. A. Clemons, A. F. Shamji, S. L. Schreiber, An interactive resource to identify cancer genetic and lineage dependencies targeted by small molecules. *Cell* **154**, 1151–1161 (2013).

17. M. G. Rees, B. Seashore-Ludlow, J. H. Cheah, D. J. Adams, E. V. Price, S. Gill, S. Javaid, M. E. Coletti, V. L. Jones, N. E. Bodycombe, C. K. Soule, B. Alexander, A. Li, P. Montgomery, J. D. Kotz, C. S.-Y. Hon, B. Munoz, T. Liefeld, V. Dančík, D. A. Haber, C. B. Clish, J. A. Bittker, M. Palmer, B. K. Wagner, P. A. Clemons, A. F. Shamji, S. L. Schreiber, Correlating chemical sensitivity and basal gene expression reveals mechanism of action. *Nat. Chem. Biol.* **12**, 109–116 (2016).

18. S. M. Corsello, R. T. Nagari, R. D. Spangler, J. Rossen, M. Kocak, J. G. Bryan, R. Humeidi, D. Peck, X. Wu, A. A. Tang, V. M. Wang, S. A. Bender, E. Lemire, R. Narayan, P. Montgomery, U. Ben-David, C. W. Garvie, Y. Chen, M. G. Rees, N. J. Lyons, J. M. McFarland, B. T. Wong, L. Wang, N. Dumont, P. J. O'Hearn, E. Stefan, J. G. Doench, C. N. Harrington, H. Greulich, M. Meyerson, F. Vazquez, A. Subramanian, J. A. Roth, J. A. Bittker, J. S. Boehm, C. C. Mader, A. Tsherniak, T. R. Golub, Discovering the anticancer potential of non-oncology drugs by systematic viability profiling. *Nat. Cancer* **1**, 235–248 (2020).

19. B. Kumar, A. K. Adebayo, M. Prasad, M. L. Capitano, R. Wang, P. Bhat-Nakshatri, M. Anjanappa, E. Simpson, D. Chen, Y. Liu, J. M. Schilder, A. B. Colter, C. Maguire, C. J. Temm, G. Sandusky, E. H. Doud, A. B. Wijeratne, A. L. Mosley, H. E. Broxmeyer, H. Nakshatri, Tumor collection/processing under physioxia uncovers highly relevant signaling networks and drug sensitivity. *Sci. Adv.* **8**, eabh3375 (2022).

20. H. A. Kenny, M. Lal-Nag, E. A. White, M. Shen, C.-Y. Chiang, A. K. Mitra, Y. Zhang, M. Curtis, E. M. Schryver, S. Bettis, A. Jadhav, M. B. Boxer, Z. Li, M. Ferrer, E. Lengyel, Quantitative high throughput screening using a primary human three-dimensional organotypic culture predicts in vivo efficacy. *Nat. Commun.* **6**, 6220 (2015).

21. M. van de Wetering, H. E. Francies, J. M. Francis, G. Bounova, F. Iorio, A. Pronk, W. van Houdt, J. van Gorp, A. Taylor-Weiner, L. Kester, A. McLaren-Douglas, J. Blokker, S. Jaksani, S. Bartfeld, R. Volckman, P. van Sluis, V. S. W. Li, S. Seepo, C. Sekhar Pedamallu, K. Cibulskis, S. L. Carter, A. McKenna, M. S. Lawrence, L. Lichtenstein, C. Stewart, J. Koster, R. Versteeg, A. van Oudenaarden, J. Saez-Rodriguez, R. G. J. Vries, G. Getz, L. Wessels, M. R. Stratton, U. McDermott, M. Meyerson, M. J. Garnett, H. Clevers, Prospective derivation of a living organoid biobank of colorectal cancer patients. *Cell* **161**, 933–945 (2015).

22. F. Weeber, S. N. Ooft, K. K. Dijkstra, E. E. Voest, Tumor organoids as a pre-clinical cancer model for drug discovery. *Cell Chem. Biol.* **24**, 1092–1100 (2017).

23. D. W. McMillin, J. M. Negri, C. S. Mitsiades, The role of tumour–stromal interactions in modifying drug response: Challenges and opportunities. *Nat. Rev. Drug Discov.* **12**, 217–228 (2013).

24. S. A. Herbst, V. Kim, T. Roider, E. C. Schitter, P.-M. Bruch, N. Liebers, C. Kolb, M. Knoll, J. Lu, P. Dreger, C. Müller-Tidow, T. Zenz, W. Huber, S. Dietrich, Comparing the value of mono- versus coculture for high-throughput compound screening in hematological malignancies. *Blood Adv.* **7**, 5925–5936 (2023).

25. A. Muir, M. G. Vander Heiden, The nutrient environment affects therapy. *Science* **360**, 962–963 (2018).

26. K. L. Abbott, A. Ali, D. Casalena, B. T. Do, R. Ferreira, J. H. Cheah, C. K. Soule, A. Deik, T. Kunchok, D. R. Schmidt, S. Renner, S. E. Honeder, M. Wu, S. H. Chan, T. Tseyang, A. T. Stoltzfus, S. L. J. Michel, D. Greaves, P. P. Hsu, C. W. Ng, C. J. Zhang, A. Farsidjani, J. R. Kent, M. L. L. Madariaga, I. M. T. Gramatikov, N. J. Matheson, C. A. Lewis, C. B. Clish, M. G. Rees, J. A. Roth, L. M. Griner, A. Muir, D. S. Auld, M. G. V. Heiden, Screening in serum-derived medium reveals differential response to compounds targeting metabolism. *Cell Chem. Biol.* **30**, 1156–1168.e7 (2023).

27. N. J. Rossiter, K. S. Huggler, C. H. Adelmann, H. R. Keys, R. W. Soens, D. M. Sabatini, J. R. Cantor, CRISPR screens in physiologic medium reveal conditionally essential genes in human cells. *Cell Metab.* **33**, 1248–1263.e9 (2021).

28. L. A. M. Griner, R. Guha, P. Shinn, R. M. Young, J. M. Keller, D. Liu, I. S. Goldlust, A. Yasgar, C. McKnight, M. B. Boxer, D. Y. Duveau, J.-K. Jiang, S. Michael, T. Mierzwa, W. Huang, M. J. Walsh, B. T. Mott, P. Patel, W. Leister, D. J. Maloney, C. A. Leclair, G. Rai, A. Jadhav, B. D. Peyser, C. P. Austin, S. E. Martin, A. Simeonov, M. Ferrer, L. M. Staudt, C. J. Thomas, High-throughput combinatorial screening identifies drugs that cooperate with ibrutinib to kill activated B-cell–like diffuse large B-cell lymphoma cells. *Proc. Natl. Acad. Sci. U.S.A.* **111**, 2349–2354 (2014).

29. J. Shelton, X. Lu, J. A. Hollenbaugh, J. H. Cho, F. Amblard, R. F. Schinazi, Metabolism, biochemical actions, and chemical synthesis of anticancer nucleosides, nucleotides, and base analogs. *Chem. Rev.* **116**, 14379–14455 (2016).

30. C. Avendaño, J. C. Menéndez, "Chapter 5: DNA alkylating gents" in *Medicinal Chemistry of Anticancer Drugs (Second Edition)* (Elsevier Science, 2015), pp. 197–238.

31. E. S. Ali, I. Ben-Sahra, Regulation of nucleotide metabolism in cancers and immune disorders. *Trends Cell Biol.* **33**, 950–966 (2023).

32. M. Visentin, R. Zhao, I. D. Goldman, The antifolates. *Hematol. Oncol. Clin. North Am.* **26**, 629–648 (2012).

33. P. M. Wilson, P. V. Danenberg, P. G. Johnston, H.-J. Lenz, R. D. Ladner, Standing the test of time: Targeting thymidylate biosynthesis in cancer therapy. *Nat. Rev. Clin. Oncol.* **11**, 282–298 (2014).

34. N. J. Mullen, P. K. Singh, Nucleotide metabolism: A pan-cancer metabolic dependency. *Nat. Rev. Cancer* **23**, 275–294 (2023).

35. G. S. Ducker, J. D. Rabinowitz, One-carbon metabolism in health and disease. *Cell Metab.* **25**, 27–42 (2016).

36. Y. Zheng, L. C. Cantley, Toward a better understanding of folate metabolism in health and disease. *J. Exp. Med.* **216**, 253–266 (2019).

37. Z. E. Stine, Z. T. Schug, J. M. Salvino, C. V. Dang, Targeting cancer metabolism in the era of precision oncology. *Nat. Rev. Drug Discov.* **21**, 141–162 (2022).

38. E. S. J. Arnér, S. Eriksson, Mammalian deoxyribonucleoside kinases. *Pharmacol. Ther.* **67**, 155–186 (1995).

39. S. B. Howell, S. J. Mansfield, R. Taetle, Significance of variation in serum thymidine concentration for the marrow toxicity of methotrexate. *Cancer Chemother. Pharmacol.* **5**, 221–226 (1981).

40. M. H. N. Tattersall, B. Brown, E. Frei III, The reversal of methotrexate toxicity by thymidine with maintenance of antitumour effects. *Nature* **253**, 198–200 (1975).

41. T. J. V. Mouwerik, C. A. Pangallo, J. K. V. Willson, P. H. Fischer, Augmentation of methotrexate cytotoxicity in human colon cancer cells achieved through inhibition of thymidine salvage by dipyridamole. *Biochem. Pharmacol.* **36**, 809–814 (1987).

42. G. Urlaub, L. A. Chasin, Isolation of Chinese hamster cell mutants deficient in dihydrofolate reductase activity. *Proc. Natl. Acad. Sci. U.S.A.* **77**, 4216–4220 (1980).

43. S. B. Howell, S. J. Mansfield, R. Taetle, Thymidine and hypoxanthine requirements of normal and malignant human cells for protection against methotrexate cytotoxicity. *Cancer Res.* **41**, 945–950 (1981).

44. Y. Santiago, E. Chan, P.-Q. Liu, S. Orlando, L. Zhang, F. D. Urnov, M. C. Holmes, D. Guschin, A. Waite, J. C. Miller, E. J. Rebar, P. D. Gregory, A. Klug, T. N. Collingwood, Targeted gene knockout in mammalian cells by using engineered zinc-finger nucleases. *Proc. Natl. Acad. Sci. U.S.A.* **105**, 5809–5814 (2008).

45. Y. Zheng, T.-Y. Lin, G. Lee, M. N. Paddock, J. Momb, Z. Cheng, Q. Li, D. L. Fei, B. D. Stein, S. Ramsamooj, G. Zhang, J. Blenis, L. C. Cantley, Mitochondrial one-carbon pathway supports cytosolic folate integrity in cancer cells. *Cell* **175**, 1546–1560.e17 (2018).

46. J. C. Drake, C. J. Allegra, P. G. Johnston, Immunological quantitation of thymidylate synthase-FdUMP-5,10-methylenetetrahydrofolate ternary complex with the monoclonal antibody TS 106. *Anticancer Drugs* **4**, 431–436 (1993).

47. C. W. Carreras, D. V. Santi, The catalytic mechanism and structure of thymidylate synthase. *Annu. Rev. Biochem.* **64**, 721–762 (1995).

48. D. B. Longley, D. P. Harkin, P. G. Johnston, 5-Fluorouracil: Mechanisms of action and clinical strategies. *Nat. Rev. Cancer* **3**, 330–338 (2003).

49. E. De Clercq, A 40-year journey in search of selective antiviral chemotherapy. *Annu. Rev. Pharmacol.* **51**, 1–24 (2011).

50. G. Andrei, R. Snoeck, Advances and perspectives in the management of varicella-zoster virus infections. *Molecules* **26**, 1132 (2021).

51. J. Balzarini, E. De Clercq, A. Verbruggen, D. Ayusawa, K. Shimizu, T. Seno, Thymidylate synthase is the principal target enzyme for the cytostatic activity of (E)-5-(2-bromovinyl)-2′-deoxyuridine against murine mammary carcinoma (FM3A) cells transformed with the herpes simplex virus type 1 or type 2 thymidine kinase gene. *Mol. Pharmacol.* **32**, 410–416 (1987).

52. E. De Clercq, Discovery and development of BVDU (brivudin) as a therapeutic for the treatment of herpes zoster. *Biochem. Pharmacol.* **68**, 2301–2315 (2004).

53. E. De Clercq, In search of a selective antiviral chemotherapy. *Clin. Microbiol. Rev.* **10**, 674–693 (1997).

54. K. Hew, S.-L. Dahlroth, S. Veerappan, L. X. Pan, T. Cornvik, P. Nordlund, Structure of the varicella zoster virus thymidylate synthase establishes functional and structural similarities as the human enzyme and potentiates itself as a target of brivudine. *PLOS ONE* **10**, e0143947 (2015).

55. G. Gáspár, E. De Clercq, J. Neyts, Human herpesvirus 8 gene encodes a functional thymidylate synthase. *J. Virol.* **76**, 10530–10532 (2002).

56. S. Eriksson, B. Kierdaszuk, B. Munch-Petersen, B. Oberg, N. G. Johansson, Comparison of the substrate specificities of human thymidine kinase 1 and 2 and deoxycytidine kinase toward antiviral and cytostatic nucleoside analogs. *Biochem. Biophys. Res. Commun.* **176**, 586–592 (1991).

57. M. Johansson, A. R. V. Rompay, B. Degrève, J. Balzarini, A. Karlsson, Cloning and characterization of the multisubstrate deoxyribonucleoside kinase of *Drosophila* melanogaster. *J. Biol. Chem.* **274**, 23814–23819 (1999).

58. E. Franzolin, C. Rampazzo, M.-J. Pérez-Pérez, A.-I. Hernández, J. Balzarini, V. Bianchi, Bromovinyl-deoxyuridine: A selective substrate for mitochondrial thymidine kinase in cell extracts. *Biochem. Biophys. Res. Commun.* **344**, 30–36 (2006).

59. H. Eagle, Nutrition needs of mammalian cells in tissue culture. *Science* **122**, 501–504 (1955).

60. Z. Fazili, C. M. Pfeiffer, M. Zhang, Comparison of serum folate species analyzed by LC-MS/MS with total folate measured by microbiologic assay and Bio-Rad radioassay. *Clin. Chem.* **53**, 781–784 (2007).

61. C. M. Pfeiffer, M. R. Sternberg, Z. Fazili, D. A. Lacher, M. Zhang, C. L. Johnson, H. C. Hamner, R. L. Bailey, J. I. Rader, S. Yamini, R. J. Berry, E. A. Yetley, Folate status and concentrations of serum folate forms in the US population: National Health and Nutrition Examination Survey 2011–2. *Br. J. Nutr.* **113**, 1965–1977 (2015).

62. P. F. Jacques, J. Selhub, A. G. Bostom, P. W. F. Wilson, I. H. Rosenberg, The effect of folic acid fortification on plasma folate and total homocysteine concentrations. *N. Engl. J. Med.* **340**, 1449–1454 (1999).

63. G. R. Westerhof, J. H. Schornagel, I. Kathmann, A. L. Jackman, A. Rosowsky, R. A. Forsch, J. B. Hynes, F. T. Boyle, G. J. Peters, H. M. Pinedo, Carrier- and receptor-mediated transport of folate antagonists targeting folate-dependent enzymes: Correlates of molecular-structure and biological activity. *Mol. Pharmacol.* **48**, 459–471 (1995).

64. G. S. Ducker, L. Chen, R. J. Morscher, J. M. Ghergurovich, M. Esposito, X. Teng, Y. Kang, J. D. Rabinowitz, Reversal of cytosolic one-carbon flux compensates for loss of the mitochondrial folate pathway. *Cell Metab.* **23**, 1140–1153 (2016).

65. T. Hart, A. H. Y. Tong, K. Chan, J. V. Leeuwen, A. Seetharaman, M. Aregger, M. Chandrashekhar, N. Hustedt, S. Seth, A. Noonan, A. Habsid, O. Sizova, L. Nedyalkova, R. Climie, L. Tworzyanski, K. Lawson, M. A. Sartori, S. Alibeh, D. Tieu, S. Masud, P. Mero, A. Weiss, K. R. Brown, M. Usaj, M. Billmann, M. Rahman, M. Costanzo, C. L. Myers, B. J. Andrews, C. Boone, D. Durocher, J. Moffat, Evaluation and design of genome-wide CRISPR/SpCas9 knockout screens. *G3* **7**, 2719–2727 (2017).

66. Z.-S. Chen, K. Lee, S. Walther, R. B. Raftogianis, M. Kuwano, H. Zeng, G. D. Kruh, Analysis of methotrexate and folate transport by multidrug resistance protein 4 (ABCC4): MRP4 is a component of the methotrexate efflux system. *Cancer Res.* **62**, 3144–3150 (2002).

67. A. A. Demin, K. Hirota, M. Tsuda, M. Adamowicz, R. Hailstone, J. Brazina, W. Gittens, I. Kalasova, Z. Shao, S. Zha, H. Sasanuma, H. Hanzlikova, S. Takeda, K. W. Caldecott, XRCC1 prevents toxic PARP1 trapping during DNA base excision repair. *Mol. Cell* **81**, 3018–3030.e5 (2021).

68. K. N. Mohni, S. R. Wessel, R. Zhao, A. C. Wojciechowski, J. W. Luzwick, H. Layden, B. F. Eichman, P. S. Thompson, K. P. M. Mehta, D. Cortez, HMCES maintains genome integrity by shielding abasic sites in single-strand DNA. *Cell* **176**, 144–153.e13 (2019).

69. L. Chen, Z. Zhang, A. Hoshino, H. D. Zheng, M. Morley, Z. Arany, J. D. Rabinowitz, NADPH production by the oxidative pentose-phosphate pathway supports folate metabolism. *Nat. Metab.* **1**, 404–415 (2019).

70. P. J. Stover, M. S. Field, Trafficking of intracellular folates. *Adv. Nutr.* **2**, 325–331 (2011).

71. M. R. Sullivan, A. M. Darnell, M. F. Reilly, T. Kunchok, L. Joesch-Cohen, D. Rosenberg, A. Ali, M. G. Rees, J. A. Roth, C. A. Lewis, M. G. V. Heiden, Methionine synthase is essential for cancer cell proliferation in physiological folate environments. *Nat. Metab.* **3**, 1500–1511 (2021).

72. J. M. Ghergurovich, X. Xu, J. Z. Wang, L. Yang, R.-P. Ryseck, L. Wang, J. D. Rabinowitz, Methionine synthase supports tumour tetrahydrofolate pools. *Nat. Metab.* **3**, 1512–1520 (2021).

73. J. Kim, Y. Lei, J. Guo, S.-E. Kim, B. J. Wlodarczyk, R. M. Cabrera, Y. L. Lin, T. K. Nilsson, T. Zhang, A. Ren, L. Wang, Z. Yuan, Y.-F. Zheng, H.-Y. Wang, R. H. Finnell, Formate rescues neural tube defects caused by mutations in Slc25a32. *Proc. Natl. Acad. Sci. U.S.A.* **115**, 4690–4695 (2018).

74. D. R. Minton, M. Nam, D. J. McLaughlin, J. Shin, E. C. Bayraktar, S. W. Alvarez, V. O. Sviderskiy, T. Papagiannakopoulos, D. M. Sabatini, K. Birsoy, R. Possemato, Serine catabolism by SHMT2 is required for proper mitochondrial translation initiation and maintenance of formylmethionyl-tRNAs. *Mol. Cell* **69**, 610–621.e5 (2018).

75. A. S. Tibbetts, D. R. Appling, Compartmentalization of mammalian folate-mediated one-carbon metabolism. *Annu. Rev. Nutr.* **30**, 57–81 (2010).

76. K. E. Christensen, H. Patel, U. Kuzmanov, N. R. Mejia, R. E. MacKenzie, Disruption of the Mthfd1 gene reveals a monofunctional 10-formyltetrahydrofolate synthetase in mammalian mitochondria. *J. Biol. Chem.* **280**, 7597–7602 (2005).

77. N. Bonagas, N. M. S. Gustafsson, M. Henriksson, P. Marttila, R. Gustafsson, E. Wiita, S. Borhade, A. C. Green, K. S. A. Vallin, A. Sarno, R. Svensson, C. Göktürk, T. Pham, A.-S. Jemth, O. Loseva, V. Cookson, N. Kiweler, L. Sandberg, A. Rasti, J. E. Unterlass, M. Haraldsson, Y. Andersson, E. R. Scaletti, C. Bengtsson, C. B. J. Paulin, K. Sanjiv, E. Abdurakhmanov, L. Pudelko, B. Kunz, M. Desroses, P. Iliev, K. Färnegårdh, A. Krämer, N. Garg, M. Michel, S. Häggblad, M. Jarvius, C. Kalderén, A. B. Jensen, I. Almlöf, S. Karsten, S. M. Zhang, M. Häggblad, A. Eriksson, J. Liu, B. Glinghammar, N. Nekhotiaeva, F. Klingegård, T. Koolmeister, U. Martens, S. Llona-Minguez, R. Moulson, H. Nordström, V. Parrow, L. Dahllund, B. Sjöberg, I. L. Vargas, D. D. Vo, J. Wannberg, S. Knapp, H. E. Krokan, P. I. Arvidsson, M. Scobie, J. Meiser, P. Stenmark, U. W. Berglund, E. J. Homan, T. Helleday, Pharmacological targeting of MTHFD2 suppresses acute myeloid leukemia by inducing thymidine depletion and replication stress. *Nat. Cancer* **3**, 156–172 (2022).

78. R. Gustafsson, A.-S. Jemth, N. M. S. Gustafsson, K. Färnegårdh, O. Loseva, E. Wiita, N. Bonagas, L. Dahllund, S. Llona-Minguez, M. Häggblad, M. Henriksson, Y. Andersson, E. Homan, T. Helleday, P. Stenmark, Crystal structure of the emerging cancer target MTHFD2 in complex with a substrate-based inhibitor. *Cancer Res.* **77**, 937–948 (2017).

79. A. C. Green, P. Marttila, N. Kiweler, C. Chalkiadaki, E. Wiita, V. Cookson, A. Lesur, K. Eiden, F. Bernardin, K. S. A. Vallin, S. Borhade, M. Long, E. K. Ghahe, J. J. Jiménez-Alonso, A.-S. Jemth, O. Loseva, O. Mortusewicz, M. Meyers, E. Viry, A. I. Johansson, O. Hodek, E. Homan, N. Bonagas, L. Ramos, L. Sandberg, M. Frödin, E. Moussay, A. Slipicevic, E. Letellier, J. Paggetti, C. S. Sørensen, T. Helleday, M. Henriksson, J. Meiser, Formate overflow drives toxic folate trapping in MTHFD1 inhibited cancer cells. *Nat. Metab.* **5**, 642–659 (2023).

80. J. L. Tonkinson, L. L. Habeck, J. E. Toth, L. G. Mendelsohn, J. Bewley, K. A. Shackelford, S. B. Gates, J. Ray, V. J. Chen, The antiproliferative and cell cycle effects of 5,6,7, 8-tetrahydro-N5,N10-carbonylfolic acid, an inhibitor of methylenetetrahydrofolate dehydrogenase, are potentiated by hypoxanthine. *J. Pharmacol. Exp. Ther.* **287**, 315–321 (1998).

81. L. R. Celeste, G. Chai, M. Bielak, W. Minor, L. L. Lovelace, L. Lebioda, Mechanism of N10-formyltetrahydrofolate synthetase derived from complexes with intermediates and inhibitors. *Protein Sci.* **21**, 219–228 (2012).

82. X. Rabasseda, Brivudine: A herpes virostatic with rapid antiviral activity and once-daily dosing. *Drugs Today* **39**, 359–371 (2003).

83. A. Neschadim, J. C. Wang, T. Sato, D. H. Fowler, A. Lavie, J. A. Medin, Cell fate control gene therapy based on engineered variants of human deoxycytidine kinase. *Mol. Ther.* **20**, 1002–1013 (2012).

84. S. Campion, J. Aubrecht, K. Boekelheide, D. W. Brewster, V. S. Vaidya, L. Anderson, D. Burt, E. Dere, K. Hwang, S. Pacheco, J. Saikumar, S. Schomaker, M. Sigman, F. Goodsaid, The current status of biomarkers for predicting toxicity. *Expert Opin. Drug Metab. Toxicol.* **9**, 1391–1408 (2013).

85. J. K. Martin II, J. P. Sheehan, B. P. Bratton, G. M. Moore, A. Mateus, S. H.-J. Li, H. Kim, J. D. Rabinowitz, A. Typas, M. M. Savitski, M. Z. Wilson, Z. Gitai, A dual-mechanism antibiotic kills Gram-negative bacteria and avoids drug resistance. *Cell* **181**, 1518–1532.e14 (2020).

86. P. Ramadurgum, D. R. Woodard, S. Daniel, H. Peng, P. L. Mallipeddi, H. Niederstrasser, M. Mihelakis, V. Q. Chau, P. M. Douglas, B. A. Posner, J. D. Hulleman, Simultaneous control of endogenous and user-defined genetic pathways using unique ecDHFR pharmacological chaperones. *Cell Chem. Biol.* **27**, 622–634.e6 (2020).

87. M. I. Gross, S. D. Demo, J. B. Dennison, L. Chen, T. Chernov-Rogan, B. Goyal, J. R. Janes, G. J. Laidig, E. R. Lewis, J. Li, A. L. MacKinnon, F. Parlati, M. L. M. Rodriguez, P. J. Shwonek, E. B. Sjogren, T. F. Stanton, T. Wang, J. Yang, F. Zhao, M. K. Bennett, Antitumor activity of the glutaminase inhibitor CB-839 in triple-negative breast cancer. *Mol. Cancer Ther.* **13**, 890–901 (2014).

88. R. S. Varughese, W. S.-T. Lam, A. A. B. H. Marican, S. H. Viganeshwari, A. S. Bhave, N. L. Syn, J. Wang, A. L.-A. Wong, A. P. Kumar, P. E. Lobie, S. C. Lee, G. Sethi, B. C. Goh, L. Wang, Biopharmacological considerations for accelerating drug development of deguelin, a rotenoid with potent chemotherapeutic and chemopreventive potential. *Cancer* **125**, 1789–1798 (2019).

89. X. Cai, Y. Xu, A. K. Cheung, R. C. Tomlinson, A. Alcázar-Román, L. Murphy, A. Billich, B. Zhang, Y. Feng, M. Klumpp, J.-M. Rondeau, A. N. Fazal, C. J. Wilson, V. Myer, G. Joberty, T. Bouwmeester, M. A. Labow, P. M. Finan, J. A. Porter, H. L. Ploegh, D. Baird, P. De Camilli, J. A. Tallarico, Q. Huang, PIKfyve, a class III PI kinase, is the target of the small molecular IL-12/IL-23 inhibitor apilimod and a player in Toll-like receptor signaling. *Chem. Biol.* **20**, 912–921 (2013).

90. S. Gayle, S. Landrette, N. Beeharry, C. Conrad, M. Hernandez, P. Beckett, S. M. Ferguson, T. Mandelkern, M. Zheng, T. Xu, J. Rothberg, H. Lichenstein, Identification of apilimod as a first-in-class PIKfyve kinase inhibitor for treatment of B-cell non-Hodgkin lymphoma. *Blood* **129**, 1768–1778 (2017).

91. R. M. Meyers, J. G. Bryan, J. M. McFarland, B. A. Weir, A. E. Sizemore, H. Xu, N. V. Dharia, P. G. Montgomery, G. S. Cowley, S. Pantel, A. Goodale, Y. Lee, L. D. Ali, G. Jiang, R. Lubonja, W. F. Harrington, M. Strickland, T. Wu, D. C. Hawes, V. A. Zhivich, M. R. Wyatt, Z. Kalani, J. J. Chang, M. Okamoto, K. Stegmaier, T. R. Golub, J. S. Boehm, F. Vazquez, D. E. Root, W. C. Hahn, A. Tsherniak, Computational correction of copy number effect improves specificity of CRISPR–Cas9 essentiality screens in cancer cells. *Nat. Genet.* **49**, 1779–1784 (2017).

92. Y. Qiao, J. E. Choi, J. C. Tien, S. A. Simko, T. Rajendiran, J. N. Vo, A. D. Delekta, L. Wang, L. Xiao, N. B. Hodge, P. Desai, S. Mendoza, K. Juckette, A. Xu, T. Soni, F. Su, R. Wang, X. Cao, J. Yu, I. Kryczek, X.-M. Wang, X. Wang, J. Siddiqui, Z. Wang, A. Bernard, E. Fernandez-Salas, N. M. Navone, S. J. Ellison, K. Ding, E.-L. Eskelinen, E. I. Heath, D. J. Klionsky, W. Zou, A. M. Chinnaiyan, Autophagy inhibition by targeting PIKfyve potentiates response to immune checkpoint blockade in prostate cancer. *Nat. Cancer* **2**, 978–993 (2021).

93. P. F. Zaratin, G. Petrone, M. Sbacchi, M. Garnier, C. Fossati, P. Petrillo, S. Ronzoni, G. A. M. Giardina, M. A. Scheideler, Modification of nociception and morphine tolerance by the selective opiate receptor-like orphan receptor antagonist (−)-cis-1-methyl-7-[[4-(2,6-dichlorophenyl)piperidin-1-yl]methyl]-6,7,8,9-tetrahydro-5H-benzocyclohepten-5-ol (SB-612111). *J. Pharmacol. Exp. Ther.* **308**, 454–461 (2004).

94. H. Yamada, H. Nakamoto, Y. Suzuki, T. Ito, K. Aisaka, Pharmacological profiles of a novel opioid receptor-like1 (ORL1) receptor antagonist, JTC-801. *Br. J. Pharmacol.* **135**, 323–332 (2002).

95. M. Ghandi, F. W. Huang, J. Jané-Valbuena, G. V. Kryukov, C. C. Lo, E. R. McDonald, J. Barretina, E. T. Gelfand, C. M. Bielski, H. Li, K. Hu, A. Y. Andreev-Drakhlin, J. Kim, J. M. Hess, B. J. Haas, F. Aguet, B. A. Weir, M. V. Rothberg, B. R. Paolella, M. S. Lawrence, R. Akbani, Y. Lu, H. L. Tiv, P. C. Gokhale, A. de Weck, A. A. Mansour, C. Oh, J. Shih, K. Hadi, Y. Rosen, J. Bistline, K. Venkatesan, A. Reddy, D. Sonkin, M. Liu, J. Lehar, J. M. Korn, D. A. Porter, M. D. Jones, J. Golji, G. Caponigro, J. E. Taylor, C. M. Dunning, A. L. Creech, A. C. Warren, J. M. McFarland, M. Zamanighomi, A. Kauffmann, N. Stransky, M. Imielinski, Y. E. Maruvka, A. D. Cherniack, A. Tsherniak, F. Vazquez, J. D. Jaffe, A. A.

Lane, D. M. Weinstock, C. M. Johannessen, M. P. Morrissey, F. Stegmeier, R. Schlegel, W. C. Hahn, G. Getz, G. B. Mills, J. S. Boehm, T. R. Golub, L. A. Garraway, W. R. Sellers, Next-generation characterization of the Cancer Cell Line Encyclopedia. *Nature* **569**, 503–508 (2019).

96. J. R. Cantor, V. Panayiotou, G. Agnello, G. Georgiou, E. M. Stone, Engineering reduced-immunogenicity enzymes for amino acid depletion therapy in cancer. *Methods Enzymol.* **502**, 291–319 (2012).

97. N. Kanarek, B. Petrova, D. M. Sabatini, Dietary modifications for enhanced cancer therapy. *Nature* **579**, 507–517 (2020).

98. L. Chen, G. S. Ducker, W. Lu, X. Teng, J. D. Rabinowitz, An LC-MS chemical derivatization method for the measurement of five different one-carbon states of cellular tetrahydrofolate. *Anal. Bioanal. Chem.* **409**, 5955–5964 (2017).

99. P. Stover, V. Schirch, Synthesis of (6S)-5-formyltetrahydropteroyl-polyglutamates and interconversion to other reduced pteroylpolyglutamate derivatives. *Anal. Biochem.* **202**, 82–88 (1992).

100. D. S. Wishart, Y. D. Feunang, A. Marcu, A. C. Guo, K. Liang, R. Vázquez-Fresno, T. Sajed, D. Johnson, C. Li, N. Karu, Z. Sayeeda, E. Lo, N. Assempour, M. Berjanskii, S. Singhal, D. Arndt, Y. Liang, H. Badran, J. Grant, A. Serra-Cayuela, Y. Liu, R. Mandal, V. Neveu, A. Pon, C. Knox, M. Wilson, C. Manach, A. Scalbert, HMDB 4.0: The human metabolome database for 2018. *Nucleic Acids Res.* **46**, D608–D617 (2018).

101. T. W. Traut, Physiological concentrations of purines and pyrimidines. *Mol. Cell. Biochem.* **140**, 1–22 (1994).

102. C. Ritz, F. Baty, J. C. Streibig, D. Gerhard, Dose-response analysis using R. *PLOS ONE* **10**, e0146021 (2015).

103. B. Wang, M. Wang, W. Zhang, T. Xiao, C.-H. Chen, A. Wu, F. Wu, N. Traugh, X. Wang, Z. Li, S. Mei, Y. Cui, S. Shi, J. J. Lipp, M. Hinterndorfer, J. Zuber, M. Brown, W. Li, X. S. Liu,

Integrative analysis of pooled CRISPR genetic screens using MAGeCKFlute. *Nat. Protoc.* **14**, 756–780 (2019).

104. S. Sdelci, A. F. Rendeiro, P. Rathert, W. You, J.-M. G. Lin, A. Ringler, G. Hofstätter, H. P. Moll, B. Gürtl, M. Farlik, S. Schick, F. Klepsch, M. Oldach, P. Buphamalai, F. Schischlik, P. Májek, K. Parapatics, C. Schmidl, M. Schuster, T. Penz, D. L. Buckley, O. Hudecz, R. Imre, S.-Y. Wang, H. M. Maric, R. Kralovics, K. L. Bennett, A. C. Müller, K. Mechtler, J. Menche, J. E. Bradner, G. E. Winter, K. Klavins, E. Casanova, C. Bock, J. Zuber, S. Kubicek, MTHFD1 interaction with BRD4 links folate metabolism to transcriptional regulation. *Nat. Genet.* **51**, 990–998 (2019).

105. D. Bozec, A. Sattiraju, A. Bouras, J. G. J. Raj, D. Rivera, Y. Huang, C. J. Alves, R. Tejero, N. M. Tsankova, H. Zou, C. Hadjipanayis, R. H. Friedel, Akaluc bioluminescence offers superior sensitivity to track in vivo glioma expansion. *Neurooncol. Adv.* **2**, vdaa134 (2020).

106. T. Wang, K. Birsoy, N. W. Hughes, K. M. Krupczak, Y. Post, J. J. Wei, E. S. Lander, D. M. Sabatini, Identification and characterization of essential genes in the human genome. *Science* **350**, 1096–1101 (2015).
